# Cumulative transcription factor binding and p300-mediated histone acetylation drive enhancer activation frequency

**DOI:** 10.1038/s41588-026-02703-x

**Published:** 2026-07-31

**Authors:** Valentina Baderna, Guido Barzaghi, Rozemarijn Kleinendorst, Kasit Chatsirisupachai, Laura Moniot-Perron, Colm Doyle, Meike Schopp, Tino Hochepied, Claude Libert, Duncan T. Odom, Judith B. Zaugg, Arnaud R. Krebs

**Affiliations:** 1 Genome Biology Unit, EMBL Heidelberg, Heidelberg, Germany; 2 Faculty of Biosciences, Collaboration for Joint PhD Degree between EMBL and Heidelberg University, Heidelberg, Germany; 3 German Cancer Research Center (DKFZ), Division of Regulatory Genomics and Cancer Evolution, Heidelberg, Germany; 4 VIB Center for Inflammation Research, Ghent, Belgium; 5 Department of Biomedical Molecular Biology, Ghent University, Ghent, Belgium; 6 Structural and Computational Biology Unit, EMBL Heidelberg, Heidelberg, Germany; 7 Present Address: Institut Imagine, Paris, France; 8 Present Address: Division of Precision Medicine, NYU Grossman School of Medicine, New York City, NY USA; 9 Present Address: Division of Medical Bioinformatics, Research Department, Faculty of Medicine Siriraj Hospital, Mahidol University, Bangkok, Thailand; 10 Present Address: Department of Biomedicine, University of Basel, Basel, Switzerland

**Keywords:** Epigenetics, Gene regulation, Epigenomics

## Abstract

In eukaryotes, transcription factors (TFs) must continuously compete with nucleosomes to access their binding sites, leading to cell-to-cell variability in chromatin accessibility at regulatory regions. Although critical to understand enhancer function in transcription, the mechanisms that define how frequently an enhancer is active in a cell population remain unclear. Here we used single-molecule footprinting to quantify the frequency at which chromatin is accessible at enhancers in response to TF perturbations and changes in their chromatin environment. We find that, individually, most TFs open chromatin in a small fraction of cells, and that cumulative TF binding controls enhancer activation frequency. Moreover, testing the functionality of hundreds of enhancers when inserted at an ectopic genomic location indicates that p300 activity is required for their full activation. Our data support a model in which enhancer activation frequency depends on the cumulative function of multiple TFs and is modulated by p300 activity.

## Abstract (web summary)

This study uses single-molecule footprinting to quantify chromatin accessibility at enhancers and promoters in mouse embryonic stem cells and to dissect the contributions of transcription factor binding and chromatin context.

## Main

Gene expression is regulated by the binding of transcription factors (TFs) at *cis*-regulatory elements (CREs), such as enhancers and promoters. Activation of a CRE is a multistep process that is influenced by both *cis*-encoded mechanisms, such as TF binding, and *trans*-acting mechanisms, including post-translational modification (PTM) of histone tails. TFs need to open chromatin before they can stimulate transcription through the recruitment of cofactors and the general transcription machinery^1^. Activation of most CREs requires the collective function of multiple TFs^2–5^.

Several mechanistic models have been proposed to explain how TFs combine their function to open chromatin. One model postulates that a specific class of TFs is specialized in opening chromatin (named ‘pioneer’), while other TFs depend on their prior binding to exert functions such as transcription stimulation^3,6^. Sequential binding and functional dependency across TFs have been observed in the activation of lineage-specifying CREs^3,6,7^. Additionally, only a subset of TFs bind nucleosome-wrapped DNA, while most engage only free DNA^8–11^. An alternative, complementary model proposes that most TFs exert only low levels of chromatin opening activity when binding individually, and that the cumulative function of multiple TFs is required to open chromatin at sufficient levels to activate transcription^2,3,12–14^.

Chromatin accessibility (CA) is only stable for minutes and requires continuous maintenance through the recruitment of ATP-dependent remodelers^15–17^. This constant competition between TFs and nucleosomes leads to heterogeneity in CRE accessibility, where CREs are only open in a fraction of the cells at any given time^18,19^. This cell-to-cell variability in enhancer accessibility will, for instance, condition how often an enhancer can stimulate the transcription of its target gene. Yet, the frequency at which enhancers or promoters are active in cells, as well as the molecular mechanisms involved in the regulation of their CA levels, is largely unknown. What is the contribution of individual TFs to enhancer accessibility frequency? How do multiple TFs combine their function at enhancers?

Each TF binding event occurs in a specific context defined by partner TFs and specific combinations of histone PTMs. The presence of PTMs is associated with activation or repression of transcriptional activity. For example, H3K27ac is commonly used as a marker of active CREs^20^, but its effect on CRE activity remains unclear^21^. The diversity of chromatin contexts encountered by TFs further complicates the quantification of their contribution to enhancer activation. Isolating the effect of TF binding on chromatin opening from the influence of chromatin context requires studying TF function under controlled conditions, ideally at regions where histone marks are minimal.

Measuring the frequency of CA at enhancers requires simultaneously quantifying the fraction of nucleosome-free and nucleosome-occupied DNA within a cell population. Bulk CA assays (that is, ATAC–seq) enrich for nucleosome-free DNA^22^, losing the information about the fraction of DNA molecules occupied by nucleosomes. Single-molecule footprinting (SMF) and other methylation-based footprinting assays address this issue as they profile DNA molecules regardless of their CA state^23^. These assays are performed on nuclei where chromatin-remodeling activities are reduced because cofactors, such as ATP, are lost upon permeabilization, thus generating a snapshot of CA^24^. The resulting data can be interpreted at the level of individual DNA molecules^18,23,25^, revealing the frequency at which a CRE is accessible, occupied by nucleosomes^19,26–28^ or TFs^18,25,29–31^.

Here we used SMF to quantify CA at enhancers and promoters in mouse embryonic stem cells (mESCs) and dissect the contributions of TF binding and chromatin context. We show that CA is highly variable across active CREs, with promoters accessible in most cells and enhancers only in a minority. Perturbation of TF motifs and rapid TF degradation reveal that individual TFs make partial contributions to CA, whereas full enhancer accessibility requires their cumulative binding. When assayed at an ectopic locus, most enhancers only partially recapitulate their native CA, indicating a role for chromatin context. Consistent with this, p300 inhibition reduces CA frequency at enhancers genome wide, supporting a role for p300 activity in this process. Finally, changes in CA frequency directly impact enhancer activity through altered RNA polymerase II (Pol II) recruitment.

## Results

### Frequent nucleosome occupancy at active enhancers

**Fig. 1:**
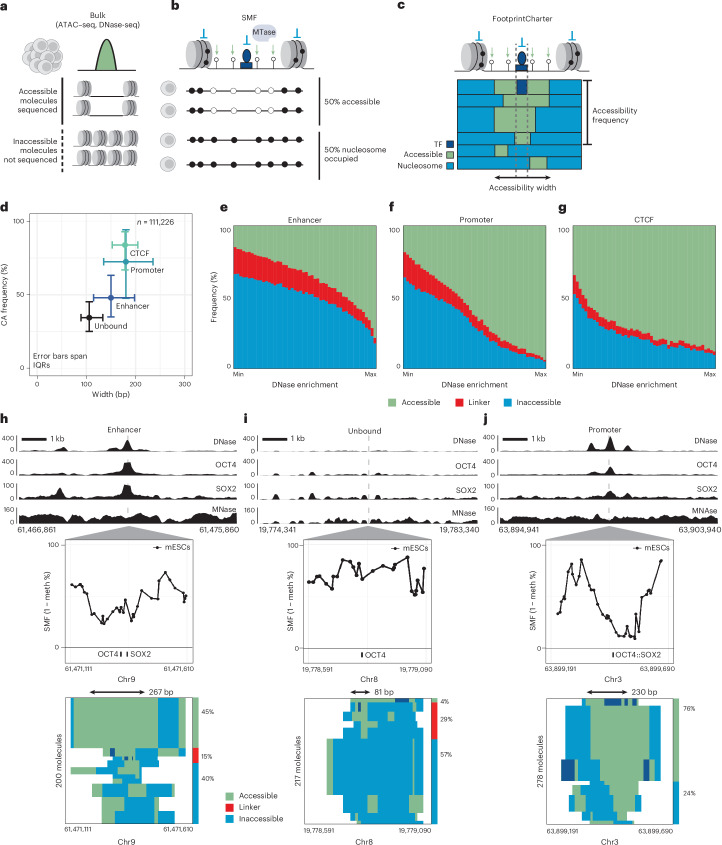
Quantification of CA frequency at CREs. **a**, Mammalian CREs are characterized by different levels of accessibility. Bulk assays, such as DNase-seq and ATAC–seq, are very useful to classify and rank open regulatory elements. However, their output is a relative enrichment of the accessibility signal and is limited in estimating CREs’ underlying frequency of accessibility across a cell population. **b**, SMF uses exogenous methyltransferases to footprint protein–DNA contacts. Profiling DNA methylation by bisulfite sequencing captures and quantifies the intercellular heterogeneity in CA at CREs with single DNA molecule resolution. **c**, FootprintCharter uses unsupervised partitioning to locate and quantify footprints for TFs and nucleosomes in SMF data. Here we use FootprintCharter to quantify the frequency of CA at TF motifs as well as the width of accessible stretches, averaged across molecules. **d**, Different CREs have different CA frequencies and width. Scatterplot showing the distributions of total CA frequency (*y* axis) and average accessibility width across molecules (*x* axis) for bound motifs at CTCF-containing loci, promoters and enhancers, and unbound motifs at intergenic regions. Dots represent median values, while error bars span IQRs, that is, first to third quartiles. **e**–**g**, Different CREs have different frequencies associated with linker DNA, accessibility and inaccessibility. Bar plots showing the distribution of molecular-state frequencies (large accessible stretches in green, linker-length accessible DNA in red and inaccessible in blue) for active enhancers (**e**), promoters (**f**) and CTCF-containing loci (**g**) as a function of DNase enrichment. The quantification of the accessible fraction includes molecules displaying TF footprints. CREs were binned based on the DNase enrichment and the median frequency was calculated for each state and each bin. **h**, Example of active enhancer harboring an OCT4 and a SOX2 motif. At the SOX2 motif, CA is detected in 60% of the cell population that is split into 15% molecules with a width compatible with linker DNA (< 100 bp) and 45% large accessibility stretches (>100 bp). Top, genomic tracks for DNase-seq, ChIP-nexus for OCT4 and SOX2, and MNase-seq around the TF motifs. Middle, the average SMF signal (1 − meth %), as well as the TF-motif annotation. Bottom, the stacks of single molecules sorted by decreasing widths of CA stretches as quantified by FootprintCharter by molecular state (accessible in green, accessible linker DNA in red and inaccessible in blue). The FootprintCharter stack is further annotated with the average width of accessibility across molecules (top arrow-headed segment) and frequencies of accessibility at active molecules (green bar) and linker DNA (red bar). **i**, Example of intergenic locus harboring an inactive and unbound OCT4 motif accessible in 33% of the cell population in almost exclusive association with linker DNA (29%). Same representation as **h**. **j**, Example of active promoter harboring a composite OCT4::SOX2 motif accessible in 76% of the cell population in exclusive association with large accessibility stretches. Same representation as **h**. IQRs, interquartile ranges; meth, methylation.

**Extended Data Fig. 1:**
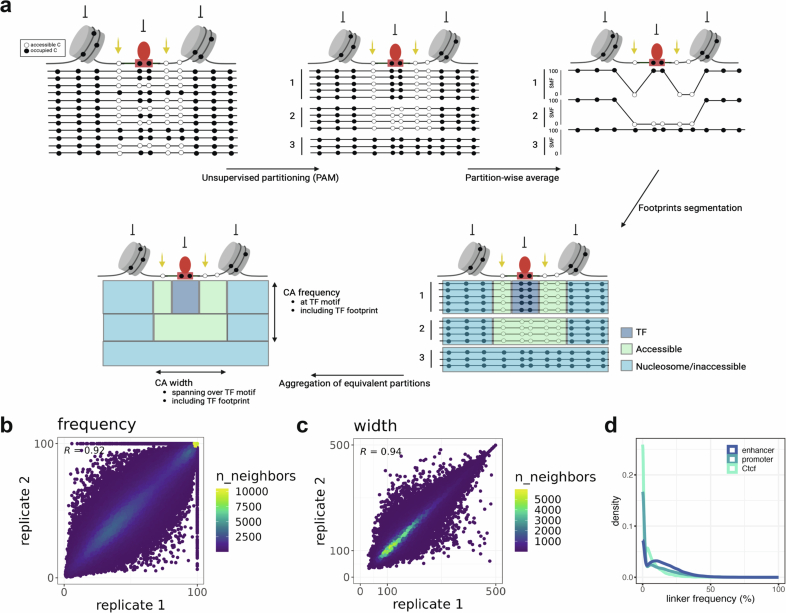
Quality controls for FootprintCharter quantifications. **a**, FootprintCharter performs unsupervised molecular partitioning using the *k*-medoids algorithm. The single-molecule footprinting (SMF) bulk signal is computed separately for each partition and used to detect transcription factors (TFs) and nucleosome footprints by frequency and width. Such footprint detection is performed agnostic of TF motif annotations. Such TF annotations are used to aggregate biologically equivalent partitions to compute the frequency and average width of chromatin accessibility at TF motifs. Schematic in **a** created in BioRender; Kleinendorst, R. https://BioRender.com/bg7pnip (2026). **b**, Scatterplot showing the replicate reproducibility of the chromatin accessibility frequency metric as calculated by FootprintCharter. The color scale indicates the number of neighboring dots, indicating the degree of overplotting. Pearson’s correlation coefficient is annotated. **c**, Scatterplot showing the replicate reproducibility of the chromatin accessibility width metric as calculated by FootprintCharter. The color scale indicates the number of neighboring dots, indicating the degree of overplotting. Pearson’s correlation coefficient is annotated. **d**, Density plot showing the distribution of frequencies of linker-length DNA accessibility across active regulatory elements.

In contrast to bulk assays, SMF captures the cell-to-cell heterogeneity of CA across individual DNA molecules (Fig. 1a,b). To quantify CA frequency at CREs, we developed an unsupervised molecular classifier named FootprintCharter^32^. The method partitions DNA molecules based on their CA patterns, distinguishing TFs and nucleosomes based on the footprint width (Fig. 1c and Extended Data Fig. 1a).

**Extended Data Fig. 2:**
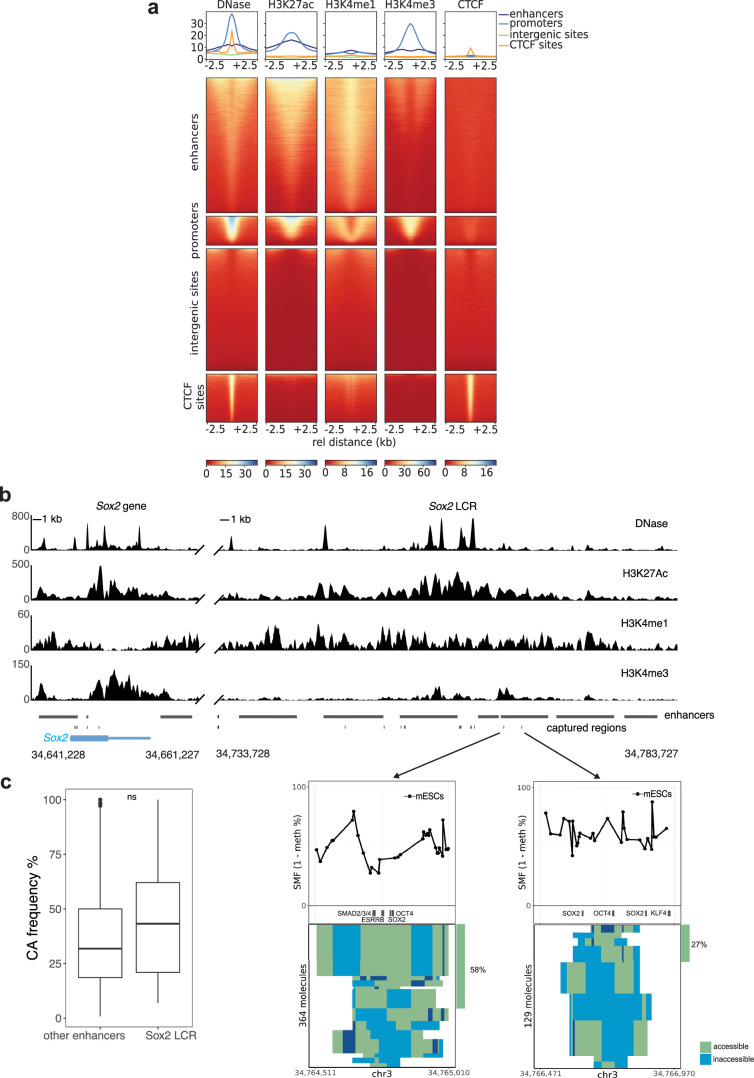
Chromatin features across *cis*-regulatory element categories. **a**, Tornado plots showing the enrichment for DNase, H3K27ac, H3K4me1, H3K4me3 and CTCF binding at the *cis*-regulatory element (CRE) types we annotated with ChromHMM. **b**, Genomic tracks for the *Sox2* gene and locus control region (LCR) reporting. The gray rectangles below the H3K4me3 track represent enhancers annotated using ChromHMM. The gray rectangles below the annotated enhancers represent the regions captured through SMF. The two SMF single loci reported below show example of those capture regions and exemplify our measurement of chromatin accessibility frequency at enhancers part of the SOX2 locus control region. **c**, Chromatin accessibility frequency distribution for all annotated enhancers at the SOX2 locus control region, as compared to the rest of the enhancers we annotate across the mouse genome.

We mapped all motif instances in the genome^33^ and identified bound motifs using reference datasets for the binding of 18 TFs active in mESCs (Supplementary Table 1; Methods). We systematically quantified the width and the frequency of CA around binding motifs of TFs using a previously generated bait-capture SMF dataset that profiles >60% CREs active in mESCs (Fig. 1c and Extended Data Fig. 1b–d)^18^. In SMF data, TF footprints are not observed for all TFs and depend on the presence of cytosines in proximity to their binding sites^18,24^. To treat all TFs equally, we only considered nucleosome footprints and classified molecules with TF footprints as accessible (Methods). We used an existing chromatin-based genome segmentation to annotate transcription factor binding sites (TFBSs) by CRE type (enhancer, promoter or CTCF;^34^ Supplementary Methods and Extended Data Fig. 2a,b).

We compared the frequency of CA around TF motifs at various CRE types (Fig. 1d). TF motifs contained within promoters or CTCF-bound loci showed pervasive CA across cells, with frequencies exceeding 70% over median stretches of ~180 bp (Fig. 1d). In contrast, TF motifs within enhancers were typically accessible in only half the molecules (48% median—blue; Fig. 1d) and over shorter stretches (150 bp median). Unbound TF motifs showed high levels of CA, with a median frequency of 35% molecules (Fig. 1d), over short stretches of accessibility of ~100 bp (Fig. 1d), consistent with nucleosome-linker length^19^. Thus, for subsequent analyses we distinguished short stretches of accessibility that may correspond to nucleosome-linker length, from larger accessibility stretches presumably created by the binding of TFs. This width-based stratification confirmed the heterogeneity at active enhancers (Fig. 1e) and highlighted the contrast with promoters or CTCF-bound regions (Fig. 1f,g). For enhancers, typically <50% of cells had accessible chromatin and a substantial portion arose from linker-length accessibility (14% median; Fig. 1e and Extended Data Fig. 1d). Linker-length accessibility was also detectable at promoters and CTCF-bound sites, albeit at lower frequencies (Fig. 1f,g and Extended Data Fig. 1d).

The heterogeneity in CA width at enhancers was further evident when looking at an individual enhancer bound by SOX2 and OCT4 (Fig. 1h). Here 45% of cells showed accessible motifs associated with continuous stretches of 267 bp. The motifs on the remaining molecules were either covered by nucleosomes (40%) or accessible through linker-length stretches (15%). Intermediate levels of CA were also detectable at well-characterized enhancers such as enhancers from the SOX2 locus-control region (Extended Data Fig. 2b,c). Short stretches of accessibility could also be observed at an example of an OCT4 motif where no binding was detected by ChIP–seq (Fig. 1i), further suggesting that these correspond to the linker across nucleosomes. This is in contrast with the promoter of the *Plch1* gene, where 76% of molecules showed accessibility stretches of >100 bp (Fig. 1j). Together, this suggests that most enhancers have CA in <50% of cells of a population at any given time.

### CA frequency correlates with the number of bound TFs

**Fig. 2:**
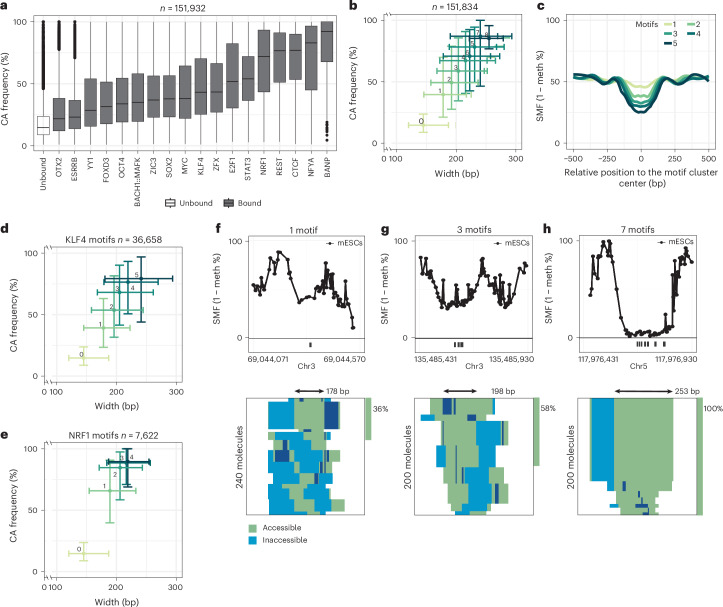
CA frequencies correlate with the number of TFs bound at the CRE. **a**, CA frequencies associated with binding of specific TFs. Box plot representing the distribution of CA frequency at motifs that are unbound (white) or bound by specific TFs (gray), as defined using ChIP–seq data. TFs with <30 quantified motifs are excluded, resulting in a total of 151,932 motifs reported. Boxes indicate medians and span the first to third IQR. The whiskers extend from the hinge to the largest value no further than ±1.5× IQR from the hinge. **b**, Frequency and width of CA scale with the number of TF binding motifs at the CRE. Scatterplot showing the distributions of accessibility frequency (*y* axis) and average width (*x* axis) at active molecules as a function of the number of active motifs at CREs (color scale). Dots represent median values, error bars span IQRs, that is, first to third quartiles. TF cluster sizes with <100 data points are excluded, resulting in a total of 151,834 motifs reported. **c**, Composite profile of SMF signal as a function of the number of active motifs at CREs. This representation shows the SMF signal (1-methylation %) for individual cytosines averaged and smoothed across heterologous CREs. **d**,**e**, Same representation as **b** but focusing on the bound motifs of KLF4 (**d**) and NRF1 (**e**). **f**–**h**, Single-locus example of enhancers with one (**f**), three (**g**) or seven (**h**) TF-bound motifs showing increasing frequencies of CA. Same representation as Fig. 1h.

**Extended Data Fig. 3:**
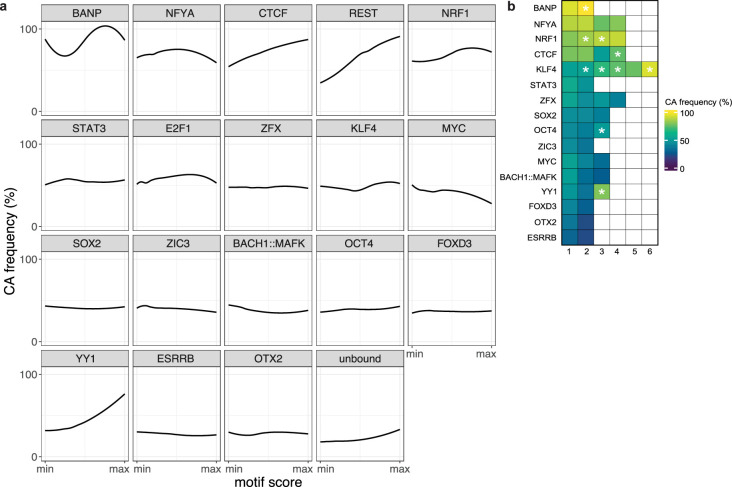
Data quality controls for TF contribution to chromatin accessibility. **a**, Smoothed conditional means displaying the chromatin accessibility frequency at transcription factor motifs, as a function of their increasing motif score. For most transcription factors, chromatin accessibility does not scale with their motif affinity. **b**, Heatmap showing the median values of chromatin accessibility frequencies at active molecules associated with an increasing number of motifs (columns) for various TFs (rows). Cells annotated with stars indicate higher frequencies as compared to CREs with one motif less for the same TF (Wilcoxon test, p < 0.05).

We then asked whether binding of specific TFs could explain the differences in the CA frequencies observed between different CREs. We compared the distribution of CA frequency at the binding sites of various TFs active in mESCs. Only five of the tested TFs were associated with a high CA frequency across their binding sites (Fig. 2a). These include not only TFs that are known to bind individually in the genome (CTCF, REST and BANP), but also the general transcriptional activators NFY and NRF1. In contrast, the binding of most TFs was associated with CA in a minority of the molecules (22–54%; Fig. 2a). These include well-characterized regulators of pluripotency, such as OCT4 and SOX2, that are critical for CA in mESCs^35,36^. We asked whether TF-motif strength influences CA frequency. We observed a scaling relationship for TFs associated with high CA frequency, such as CTCF and REST. However, this relationship did not hold for most other TFs, suggesting that motif strength alone is insufficient to explain CA frequency at target CREs (Extended Data Fig. 3a).

Because individual TFs associate with partial CA, we asked whether loci bound by multiple TFs would display higher CA frequency. We stratified the CA frequency by the number of ChIP-validated TF binding sites at the CRE. We observed that the frequency and the width of CA increased with the number of TF motifs (Fig. 2b,c). This effect is also observed when focusing on individual TFs, such as KLF4 (Fig. 2d). TFs that are associated with a higher frequency of CA such as NRF1 also showed cumulative effects, but reached saturation at a lower number of binding sites (Fig. 2e and Extended Data Fig. 3b). This gradual increase in frequency and width of CA could also be observed when inspecting examples of enhancers with increasing binding motifs (Fig. 2f–h). Taken together, these suggest that individual TF motifs, including those of pioneer TFs, drive only partial chromatin opening, whereas cumulative TF binding may explain CA frequency at CREs.

### Contribution of TFs to CA is partial and cumulative

To test this hypothesis, we measured the consequence of the perturbation of TF binding on the CA frequency at CREs. If CA is the result of the cumulative binding of multiple TFs, we would expect a partial reduction in CA frequency upon deletion of individual motifs, and that this effect would increase if multiple motifs are removed.

**Fig. 3:**
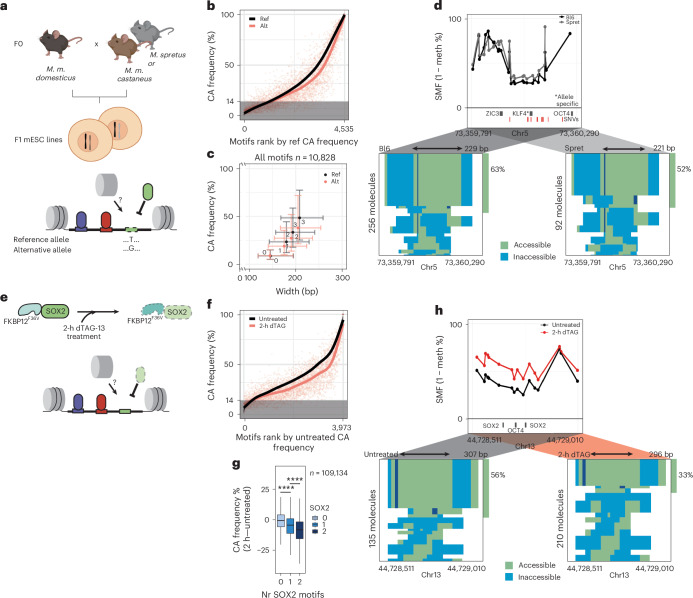
CA frequency requires cumulative function of multiple TFs. **a**, Schematic representation of the experimental strategy used to achieve systematic genetic perturbation of TF binding. We leveraged the genetic variation across evolutionarily distant mouse species and generated hybrid F1 mESCs. The two alleles in the F1 mESCs share and are exposed to the same nuclear environment, eliminating possible *trans* effects. We performed SMF in F1 mESCs derived from two different crosses and measured the allele-specific frequency of CA for TF motifs with binding affinity disrupted by SNVs on the alternative allele (the T on the reference allele is the G on the alternative allele). The disrupted affinity could abolish TF binding and promote nucleosome occupancy. **b**, Most TF disruptions lead to small changes in the frequency of CA at CREs. Scatterplot of CA frequencies in the reference allele (black) and the alternative allele (red) at loci where an SNV disrupts a TF motif. The shaded area indicates the background accessibility observed in closed chromatin. **c**, Loss of individual TFs shifts the frequency of CA at predictable levels. Scatterplot showing the allele-specific frequency of accessibility (*y* axis) and average width of accessibility (*x* axis) at active molecules as a function of the number of bound motifs at CREs (reference and alternative alleles are shown in black and red, respectively). Dots represent median values, error bars span IQRs, that is, first to third quartiles. **d**, Example locus of an enhancer bound by three TFs, among which is KLF4 with a big difference in affinity between the Bl6 and SPRET alleles. This genetic variation is associated with an 11% reduction in CA frequency in the cell population. Top, the average SMF signal (1 − meth %) for the BL6xSPRET F1 line (black dots and line for Bl6, gray dots and line for SPRET), as well as the TF motifs and SNV annotation. Bottom, FootprintCharter stacks for the Bl6 (left) and SPRET (right) alleles. **e**, Schematic representation of the experimental strategy used to test the impact of deleting individual TFs. To this end, we used rapid degradation of SOX2 through the dTAG system. The tag FKBP12-F36V is fused to SOX2. By adding the degrader molecule, dTAG, the chimeric protein is ubiquitinated and degraded by the proteasome. Schematics in **a** and **e** created in BioRender; **a**, Kleinendorst, R. https://BioRender.com/zi46lca (2026); **e**, Kleinendorst, R. https://BioRender.com/hf4sulq (2026). **f**, Loss of SOX2 leads to small changes in the frequency of CA at CREs. Scatterplot of CA frequencies in the untreated cells (black) and the cells treated for 2 h with dTAG against SOX2 (red) at SOX2-bound loci. The shaded area indicates the background accessibility observed in closed chromatin. **g**, A higher number of SOX2 motifs at the CRE increases the frequency of loss of CA upon SOX2 degradation. Box plot showing the distributions of differences in the frequency of CA (*y* axis) upon SOX2 degradation, as a function of the number of Sox2 motifs at CREs (*x* axis, color scale). The number of stars stands for the significance of the two-sided Wilcoxon test performed for each pair of consecutive categories (the exact *P* values from left to right are 2.22 × 10^−16^, 0.00022). Boxes indicate medians and span the first to third IQR. The whiskers extend from the hinge to the largest value no further than ±1.5× IQR from the hinge. **h**, Example locus of an enhancer bound by two copies of SOX2 and one of OCT4. The loss of TF binding at the SOX2 motifs is associated with a 20% reduction in CA frequency in the cell population. Top, the average SMF signal (1 − meth %) for the untreated and treated samples (black dots and line for untreated, red dots and line for 2-h dTAG), as well as the TF motifs. Bottom, FootprintCharter stacks for the untreated (left) and 2-h dTAG (right) samples. Ref, reference; alt, alternative.

**Extended Data Fig. 4:**
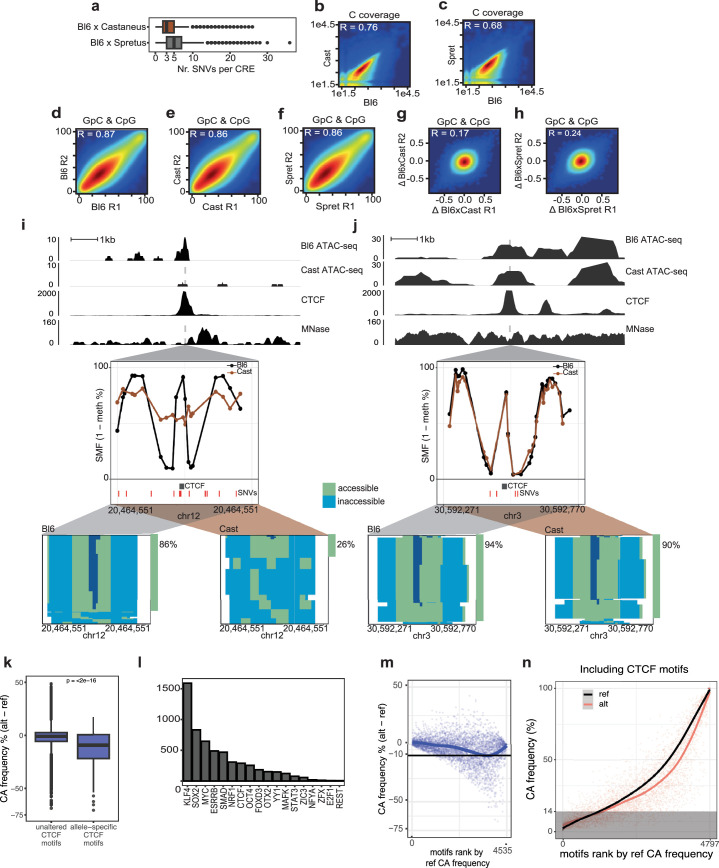
Quality controls for SMF datasets produced for mESC F1 lines. **a**, Box plots showing the distributions of number of single nucleotide variations (SNVs) per CRE in the Bl6xCast (brown) and Bl6xSpret (gray) F1s. **b**,**c**, Distribution of cytosine coverage across alleles for the Bl6xCast (**b**) and Bl6xSpret (**c**) F1s. The Pearson correlation coefficient R is annotated. **d**–**f**, Distributions of methylation rates (%) for GpCs and CpGs across biological replicates for the Bl6 (**d**), Castaneus (**e**) and Spretus (**f**) alleles. The Pearson correlation coefficient R is annotated. **g**,**h**, Distributions of methylation rate deltas across biological replicates for the Bl6xCast (**g**) and Bl6xSpret (**h**) F1s. The Pearson correlation coefficient R is annotated. **i**, Example CTCF motif with allele-specific affinity between the Bl6 and Castaneus alleles associated with 60% reduction in chromatin accessibility frequency. **j**, Example CTCF motif with no difference in affinity between the Bl6 and Castaneus alleles and no change in chromatin accessibility frequency. **k**, Box plot of chromatin accessibility (CA) frequencies between alleles (alt–ref) at unchanged and allele-specific CTCF motifs across the mouse genome. The Wilcoxon test p-value is annotated. **l**, Bar plot showing the number of TF motifs with allele-specific affinities across F1s. **m**, Scatterplot of chromatin accessibility (CA) frequencies between alleles (alt–ref), ranked by accessibility frequencies on the reference allele. **n**, Scatterplot of chromatin accessibility (CA) frequencies in the reference allele (black) and the alternative allele (red) at loci where an SNV disrupts a TF motif. The shaded area indicates the background accessibility observed in closed chromatin. Similar to Fig. 3b, but including CTCF motifs.

We leveraged the natural genetic variation across different mouse species and studied the impact of single-nucleotide variations (SNVs) that reduce TF-motif affinity on the frequency of CA. We performed SMF in two F1 lines derived from crosses between *Mus musculus domesticus* and either *Mus musculus castaneus* (Bl6xCAST) or *Mus spretus* (Bl6xSPRET; Fig. 3a). These lines have a median of three to five SNVs per CRE, respectively (Extended Data Fig. 4a). CA is measured in the same nuclear environment for both alleles, thus controlling for possible *trans* effects that may occur when comparing species.

We reproducibly quantified the CA frequencies on each allele (Extended Data Fig. 4b–h). We focused on SNVs that led to strong reductions in predicted motif affinity (Methods). To increase statistical power, we jointly analyzed the SNVs occurring between the Bl6 and the CAST or SPRET genotypes. We benchmarked our approach by measuring allele-specific occupancy changes at CTCF sites, where single motifs drive CA^9^. We observed strong allelic changes in CA frequency at loci where SNVs strongly reduce the CTCF motif affinity (Extended Data Fig. 4i), while CA is unaltered at intact motifs (Extended Data Fig. 4j,k).

**Extended Data Fig. 5:**
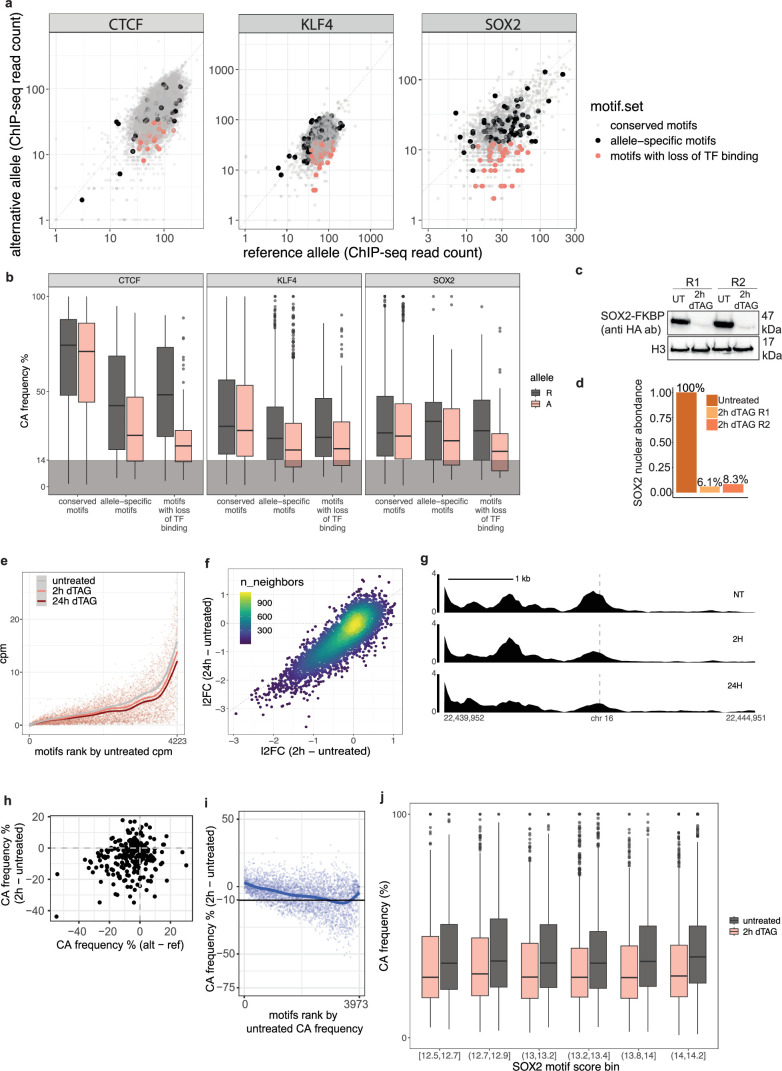
ChIP–seq validation for F1 lines SMF and quality controls for SMF datasets produced for SOX2 dTAG lines. **a**, Scatterplot showing the ChIP–seq read counts at the reference (x-axis) and alternative (y-axis) allele for the Bl6xSpretus F1 line for CTCF, KLF4 and SOX2. The motifs with conserved sequences between the species are plotted in gray, the ones with allele-specific sequence are plotted in black. The motifs with allele-specific sequence and loss of TF binding as measured by ChIP–seq are plotted in red. **b**, Box plots showing the reduction in chromatin accessibility frequency across alleles (reference in black, alternative in pink) as measured by SMF for CTCF, KLF4 and SOX2, categorized as in **a**. The shaded area indicates the background accessibility observed in closed chromatin. **c**,**d**, Western blot reporting the loss in SOX2 protein abundance upon 2 h dTAG treatment (**c**), quantified in **d**. **e**, Scatterplot of chromatin accessibility frequencies as measured by ATAC–seq for untreated cells (gray) and the cells treated for 2 h (red) or 24 h (brown) with dTAG against SOX2 at SOX2-bound loci. **f**, Scatterplot reporting the chromatin accessibility log_2_ fold change after 2 h (x-axis) or 24 h (y-axis) of dTAG treatment, as measured by ATAC–seq. **g**, Single-locus centered around a SOX2 motif exemplifying that the reduction in chromatin accessibility obtained after a 24 h SOX2 dTAG treatment is reached already with a 2 h treatment. **h**, Scatterplot comparing the chromatin accessibility (CA) frequencies in the dTAG experiment (2 h dTAG—untreated) and in the F1 mouse embryonic stem cells (mESCs) (alt–ref). **i**, Scatterplot of chromatin accessibility (CA) frequencies between alleles (2 h dTAG—untreated) at accessible molecules, ranked by accessibility frequencies in the untreated condition. **j**, Box plot showing that reduction in chromatin accessibility frequency as measured by SMF at SOX2-bound loci, as a function of SOX2 motif strength. Source data

We then excluded CTCF and focused on the remaining 4,535 motifs with differential affinities across alleles, leading to >100 individual perturbations for most of the 18 tested TFs (Extended Data Fig. 4l). We ranked TF motifs by their CA frequency on the reference allele and compared it to the alternative allele. We observed that most motif disruptions led to a moderate reduction in CA (<10% median), while complete loss of CA was rare (Fig. 3b and Extended Data Fig. 4m). Including the CTCF-bound enhancers in this analysis did not change the general trend observed and the conclusion of this analysis (Extended Data Fig. 4n). Moreover, we confirmed the loss of TF occupancy at SNV-containing motifs by ChIP–seq for CTCF, KLF4 and SOX2 (Extended Data Fig. 5a,b). We then stratified CREs by the number of TFs and quantified how disruption of individual TF motifs affected the width and frequency of CA (Fig. 3c). Regardless of the number of TFs binding the CRE, we observed a consistent drop in CA frequency. Upon loss of a single motif, CREs with *n* motifs showed distributions of CA frequencies for the alternative allele that approached the distributions of CREs with *n* − 1 motifs on the reference allele. For instance, CREs with three motifs had a CA frequency of 50% on the reference allele, and 39% on the alternative allele when one TF motif was disrupted by an SNV (Fig. 3c). This frequency was close to that of CREs bound by two TFs on the reference allele (35%). These moderate shifts in the frequency of accessible molecules were also observed at individual loci, as illustrated by a KLF4-bound enhancer that showed an 11% reduction in CA upon motif disruption (Fig. 3d). These observations suggest that loss of individual motifs leads to partial reductions in CA, with each motif contributing cumulatively and rarely more than 10% of the total CA.

To confirm these observations, we quantified the changes in CA frequency at SOX2-bound enhancers using a degron system (Fig. 3e and Extended Data Fig. 5c,d). After 2-h treatment, >90% of SOX2 is degraded and most of the CA changes at SOX2 binding sites detected in a 24-h time-course experiment already occurred (Extended Data Fig. 5c–g). Changes in CA frequency upon SOX2 degradation were in agreement with those observed across alleles in F1s (Extended Data Fig. 5h). Despite the almost complete removal of SOX2, we observed only a partial reduction in the frequency of CA, where enhancer occupancy by nucleosomes rarely increased by more than 10% molecules (Fig. 3f and Extended Data Fig. 5i). These effects are most visible for loci with intermediate CA, while highly accessible regions are less affected (Fig. 3f). This may be explained by the existence of redundant mechanisms mediating CA at these highly accessible loci. We observed similar amplitudes across various motif strength, indicating that, within this range, motifs of all strengths contribute similarly to CA (Extended Data Fig. 5j). We then asked whether multiple binding sites for the same TF would lead to cumulative effects. Indeed, enhancers containing multiple SOX2 binding sites showed a greater loss of CA (Fig. 3g). Partial CA loss can be observed at an individual enhancer bound with two SOX2 motifs that lose 20% CA upon SOX2 depletion (Fig. 3h). Together, these data support a model in which CA frequency at most enhancers is not governed by individual TFs but by the combined activity of multiple TFs.

### Measuring CA of hundreds of enhancers in parallel at an ectopic context

**Fig. 4:**
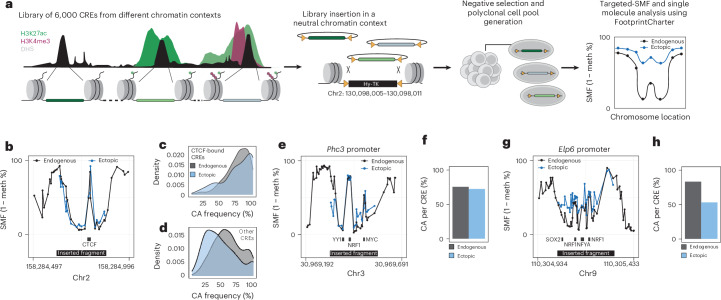
Testing the contribution of the chromatin context to the CA frequency at CREs. **a**, Schematic representation of the experimental strategy used to study the effect of the chromatin environment on CRE accessibility. A library of 6,000 DNA fragments representing entire CREs is synthesized and cloned into a plasmid flanked by asymmetric tandem lox sites for RMCE. The fragments are inserted as a pool at the landing site. Negative cell selection will enrich for cells that correctly exchanged the Hy-TK cassette by recombination, generating a polyclonal cell population where each cell contains only one insert. The frequency of CA of the inserted fragments is measured by targeted SMF and compared to that measured at the identical genetic sequence at its endogenous locus, as depicted in the cartoon. **b**, CTCF binding sites are sufficient to establish CA at the ectopic locus. Average SMF signal (1-meth %) of a CTCF-containing fragment (black rectangle) at the endogenous (black dots and line) and ectopic locus (blue dots and line). **c**,**d**, CA at the ectopic locus is shifted to lower frequencies, with the exception of CTCF-bound CREs. Density plots of the distribution of CA frequencies at the endogenous (gray) and ectopic (blue) loci for the CTCF-bound fragments contained in the library (*n* = 223; **c**) and all the non-CTCF-containing fragments (*n* = 593; **d**). **e**–**h**, Example loci that fully (**e**) or partially (**g**) establish CA at the ectopic locus. Same representation as **b**. For each locus, bar plots showing the quantification of CA frequency per CRE at the endogenous (black) and ectopic locus (blue), as measured by FootprintCharter (**f** and **h**). Hy-TK, hygromycin-thymidine kinase.

**Extended Data Fig. 6:**
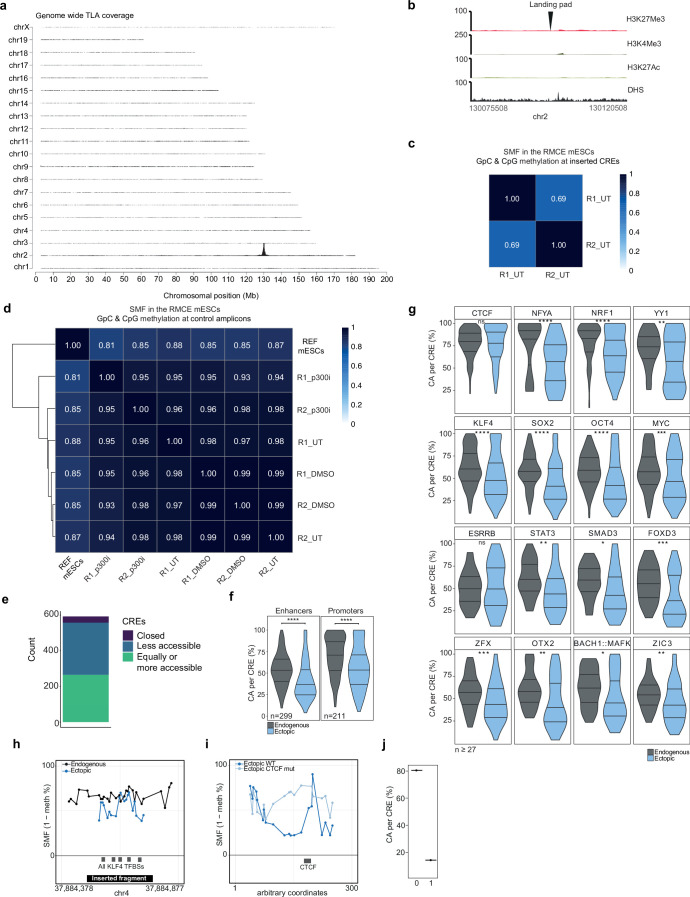
Data quality control and characterization of *cis*-regulatory elements accessibility at the neutral ectopic locus. **a**, Targeted locus amplification (TLA) signal across the mouse genome. The chromosomes and their positions are shown. The landing pad is inserted on chr2: 130,098,005–130,098,011. **b**, Genome browser tracks displaying levels of H3K27me3, H3K4me3 and H3K27ac and chromatin accessibility at the landing pad at the neutral ectopic locus (chr2: 130,075,508–130,120,508, 45 kb window of observation). **c**, Pearson correlation heatmap of the methylation rates of cytosines in different contexts (that is, CpG and GpC) across biological replicates (for a total of 322 CREs and 5,943 cytosines). Methylation rates were measured by SMF at the genomic regions inserted at the ectopic site in untreated steady-state conditions (UT); only cytosines with at least 20× coverage were retained. The dendrogram (left) shows hierarchical clustering of methylation signal, using complete-linkage method. The sample order reflects their similarity in methylation signal, with branch lengths indicating the relative distance between clusters. The Pearson correlation coefficient R is annotated. **d**, Pearson correlation heatmap of the methylation rates of cytosines in different contexts (that is, CpG and GpC) across biological replicates in untreated (UT), DMSO or p300 inhibition (p300i) conditions. Methylation rates were measured by SMF at the 4 endogenous control loci in the DNMT TKO (reference mESCs) and in the RMCE TC-1 DNMT TKO cells. Same representation as **c**. **e**, Stacked bar plot showing the number of DNA fragments that are less or equally accessible at the ectopic locus compared to their endogenous counterpart. Categories were defined as follows: ‘closed’ for CREs with chromatin accessibility ≤ 16, ‘less accessible’ for CREs showing a decrease in chromatin accessibility ≤ −10, ‘more accessible’ for CREs with an increase ≥ 10, and ‘equally accessible’ for CREs with changes between −10 and 10. **f**, Violin plots showing the frequency of chromatin accessibility at enhancers (left) and promoters (right) when measured at the endogenous loci (black) and at the ectopic locus (blue). Asterisks indicate the significance of the two-sided paired Wilcoxon signed-rank test with Benjamini–Hochberg correction for multiple comparisons (NS: adjusted p > 0.05; *: adjusted p ≤ 0.05; **: adjusted p ≤ 0.01; ***: adjusted p ≤ 0.001; ****: adjusted p ≤ 0.0001; the exact adjusted p-values from left to right are 3.00 × 10^−14^, 1.44 × 10^−12^). Horizontal lines in the violins indicate the median and the first and third quartiles. **g**, Violin plots showing the frequency of chromatin accessibility at CREs when measured at the endogenous loci (black) and at the ectopic locus (blue) as a function of the presence of specific TF motifs within the DNA fragments, as defined by ChIP–seq and motif annotation (Methods: TFBS annotation). Asterisks indicate the significance of the two-sided paired Wilcoxon signed-rank test with Benjamini–Hochberg correction for multiple comparisons (NS: adjusted p > 0.05; *: adjusted p ≤ 0.05; **: adjusted p ≤ 0.01; ***: adjusted p ≤ 0.001; ****: adjusted p ≤ 0.0001). The exact adjusted p-values and number of paired observations are: CTCF, n = 206, adjusted p = 0.935; NFYA, n = 27, adjusted p = 8.90 × 10^−5^; NRF1, n = 76, adjusted p = 8.90 × 10^−5^; YY1, n = 36, adjusted p = 1.19 × 10^−3^; KLF4, n = 370, adjusted p = 8.96 × 10^−12^; SOX2, n = 98, adjusted p = 2.17 × 10^−5^; OCT4, n = 152, adjusted p = 2.86 × 10^−5^; MYC, n = 111, adjusted p = 4.72 × 10^−4^; ESRRB, n = 38, adjusted p = 0.943; STAT3, n = 36, adjusted p = 1.39 × 10^−3^; SMAD3, n = 34, adjusted p = 1.24 × 10^−2^; FOXD3, n = 33, adjusted p = 2.29 × 10^−4^; ZFX, n = 77, adjusted p = 2.29 × 10^−4^; OTX2, n = 35, adjusted p = 2.31 × 10^−3^; BACH1::MAFK, n = 45, adjusted p = 2.88 × 10^−2^; ZIC3, n = 56, adjusted p = 2.31 × 10^−3^. Horizontal lines in the violins indicate the median and the first and third quartiles. **h**, Single-locus example of the *Mitd1* enhancer containing 5 KLF4 TFBSs that was chosen for individual and combinatorial mutant design. Average SMF signal (1-methylation %) of the fragment (black rectangle) at the endogenous (black dots and line) and ectopic locus (blue dots and line). **i**,**j**, Mutation of a CTCF binding site resulted in a complete loss of chromatin accessibility and nucleosome occupancy. Average SMF signal (1 − methylation %) of the wild-type (blue dots and line) and mutated CTCF-containing fragment (light blue dots and line) at the ectopic locus (**i**). Box plot showing the quantification of chromatin accessibility frequency per CRE as measured by FootprintCharter (**j**).

Each TF binding event occurs in a unique genomic environment characterized by specific partner TFs, as well as defined levels of chromatin modifications. Understanding how the chromatin environment impacts TF binding and CA requires a system to study TF function in a defined chromatin context. To that end, we developed a Multiplexed Chromatin-Integrated Reporter Assay (MCHIRA) to measure the ability of hundreds of CREs to establish CA at a defined ectopic genomic locus (Fig. 4a). Libraries containing hundreds of CREs were synthesized and cloned into a receiving plasmid flanked by inverted lox sites, which enable its genomic insertion through recombinase-mediated cassette exchange (RMCE). The library was inserted at a landing pad located in a neutral chromatin environment that is devoid of both activating and repressive chromatin modifications in mESCs (Extended Data Fig. 6a,b)^9,37–40^. Successful insertion events were enriched by negative selection, avoiding the potential influence of the active transcription of a positive selection marker on the CA of the tested constructs. CA of the inserted fragments was profiled by targeted SMF, unambiguously distinguishing the ectopic site from its endogenous counterpart. This led to reproducible measures of CA for hundreds of individual fragments of our library (Extended Data Fig. 6c). For each experiment, we profiled CA at four endogenous loci to detect potential batch effects in SMF (Extended Data Fig. 6d).

The library was designed to target enhancers and promoters bound by a variety of TFs in diverse chromatin contexts, consisting of 250 bp fragments spanning the entire stretch of CA at the CREs. We obtained sufficient coverage for 831 CREs (see Methods and Supplementary Table 2 for library composition). As a positive control, we included CTCF-bound loci that are able to autonomously open chromatin^9^. We indeed detected high CA at ectopic loci containing CTCF motifs (Fig. 4b,c), demonstrating the ability of MCHIRA to accurately quantify CA frequency in parallel for multiple sequences inserted at the same ectopic locus.

### Regulatory genetic information only partially determines the CA frequency

We then systematically compared the CA frequency of ectopic CREs with that of their genetically identical endogenous counterparts (excluding CTCF-containing CREs). Overall, 94% ectopic sites had CA above background (that is, accessibility observed in closed chromatin), with 47% showing comparable CA at both loci and 53% showing reduced CA at the ectopic locus (Fig. 4d and Extended Data Fig. 6e). For instance, while CA and TF footprints are recapitulated to near identical levels at the *Phc3* promoter (Fig. 4e,f), they are only partial at the *Elp6* promoter (Fig. 4g,h). We detected a substantial reduction of CA for both enhancer and promoter sequences (Extended Data Fig. 6f). These results show that a 250 bp CRE is typically sufficient to open chromatin, but most show lower CA than at the endogenous site.

**Fig. 5:**
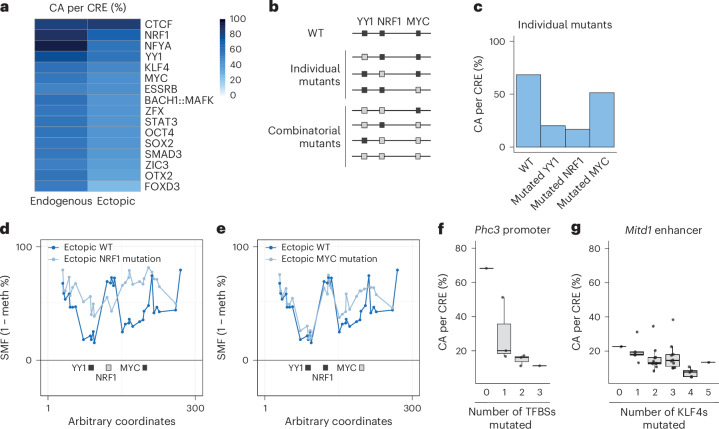
Identification of the genetic determinants of CA. **a**, TFs bind to their motifs and open chromatin at the ectopic locus, but generally to a lower extent than at endogenous motifs. Heatmap showing median CA values at endogenous and ectopic loci as a function of the presence of specific TF motifs in DNA fragments, as defined by ChIP–seq and motif annotation (Supplementary Methods). **b**, Schematic representation of the WT, individual mutants and combinatorial mutants generated at the *Phc3* promoter. Gray squares indicate intact TF motifs (YY1, NRF1, MYC), whereas light gray squares highlight the mutated motifs. **c**, Mutation of individual TF motifs results in partial loss of CA. Bar plot showing the quantification of CA frequency per CRE, as measured by FootprintCharter, for the WT and individual mutant sequences at the *Phc3* promoter. **d**,**e**, Mutation of the NRF1 (**d**) and MYC (**e**) binding sites leads to reduced CA, with each mutant displaying a different degree of accessibility loss. The average SMF signal (1 − meth %) for the WT fragment (blue dots and line) and the corresponding mutant fragments (light-blue dots and line) at the ectopic locus, with the NRF1 mutation (left) and the MYC mutation (right), are shown. The mutated TFBS is highlighted in light gray. **f**,**g**, Progressive loss of CA with increasing numbers of mutated TFBSs. Box plots show CA per CRE as a function of the number of mutated TFBSs. The **f** depicts the *Phc3* promoter, which contains binding sites for YY1, NRF1 and MYC, whereas **g** shows the *Mitd1* enhancer, with five distinct KLF4 TFBSs. For the *Phc3* promoter, *n* = 1, 3, 3, 1 distinct CRE sequences for 0, 1, 2 and 3 mutated TFBSs, respectively. For the *Mitd1* enhancer, *n* = 1, 5, 10, 10, 5, 1 distinct CRE sequences for 0, 1, 2, 3, 4 and 5 mutated KLF4 sites, respectively. Each CRE sequence was measured in two independent biological replicates. Boxes indicate medians and span the first to third IQR. The whiskers extend from the hinge to the largest value no further than ±1.5× IQR from the hinge.

To test whether specific TF motifs confer autonomous CA at the ectopic locus, we compared CA at endogenous and ectopic sites, stratified by motifs (Fig. 5a and Extended Data Fig. 6g). With the exception of CTCF, the CA frequency is consistently lower at the ectopic locus for all tested motifs (Fig. 5a). For example, fragments containing NFY and NRF1 motifs reached 56% and 67% CA on average at the ectopic site compared to >80% at endogenous loci (Fig. 5a and Extended Data Fig. 6g). In conclusion, the presence of most TF motifs is insufficient to fully recapitulate the endogenous CA. This includes TFs associated with high endogenous CA frequencies such as NFY and NRF1.

To directly test how individual TFs combine their functions to open chromatin at specific loci, we generated a library containing all combinations of TF-motif deletions for three CREs a CTCF-bound region as a positive control (Fig. 4b), an enhancer with five KLF4 motifs (Extended Data Fig. 6h) and a promoter with YY1, NRF1 and MYC motifs (Figs. 4e and 5b). As expected, disruption of the CTCF motif led to a complete loss of CA (Extended Data Fig. 6i,j). At the *Phc3* promoter, mutations of the binding sites of YY1, NRF1 and MYC resulted in a partial loss of CA (Fig. 5c). The amplitude of the loss was different for each TF, with 51.5% and 48.2% for NRF1 and YY1, respectively, and 17% when mutating the MYC motif (Fig. 5c). These mutations led to local changes in the distribution of CA depending on the position of the motif mutated (Fig. 5d,e). Combinatorial motif mutations led to increased reduction in CA compared to individual mutations (Fig. 5f). This trend was confirmed when looking at the KLF4-bound enhancer, where increasing the number of mutated binding sites decreased CA (Fig. 5g). This confirms that these TFs partially contribute to CA individually, and their combined function increases CA frequency at CREs. Moreover, CA frequency is only partially encoded in the genetic sequence, and additional mechanisms may promote higher accessibility frequency of CREs at endogenous loci.

### p300-mediated acetylation increases the CA frequency

**Fig. 6:**
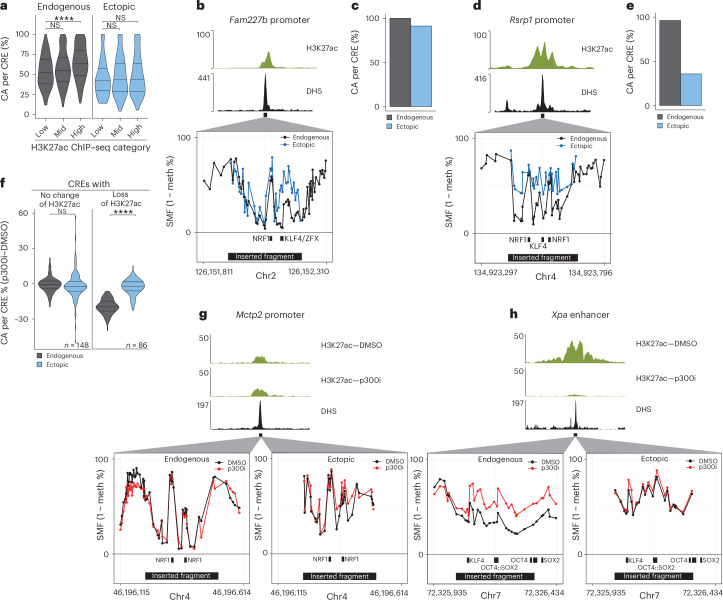
H3K27ac enhances the CA frequency at enhancers. **a**, CA frequency scales with H3K27ac levels at the endogenous loci, but not at the ectopic site. Violin plots showing the frequency of CA at CREs when measured at the endogenous (black) and ectopic (blue) loci as a function of H3K27ac ChIP–seq enrichment as measured at the endogenous loci. The number of asterisks indicates the significance of the two-sided Wilcoxon rank-sum test with Benjamini–Hochberg correction for multiple comparisons. For ectopic CREs, *n* = 597; the exact adjusted *P* values from left to right are as follows: *P*_adj_ = 0.684, 0.684. For endogenous CREs, *n* = 597; NS, ****P_adj_ ≤ 0.0001; the exact adjusted *P* values from left to right are as follows: *P*_adj_ = 0.208, 8.5 × 10^−5^. Horizontal lines in the violins indicate the median and the first and third quartiles. **b**,**c**, Single-locus example of the *Fam227b* promoter that faithfully recapitulates CA at the ectopic locus. Top, genome browser tracks of endogenous H3K27ac, and DHS. Bottom, the average SMF signals indicating the endogenous and ectopic CA frequencies. A quantification of the frequency of CA for the same fragment at the endogenous (black) and ectopic locus (blue), as measured by FootprintCharter (**c**). **d**,**e**, Single-locus example of the *Rsrp1* promoter that partially recapitulates CA at the ectopic locus. Same representation as **b** and **c**. **f**, p300i affects the frequency of CA at endogenous CREs, but not at the ectopic locus. CREs were categorized based on the statistically significant change in H3K27ac signal at the endogenous loci upon p300 inhibition. Violin plots showing the difference in the frequency of CA (CA p300i–CA DMSO) at the endogenous (black) and ectopic (blue) loci upon p300i. The number of asterisks indicates the significance of the two-sided paired Wilcoxon signed-rank test with Benjamini–Hochberg correction for multiple comparisons (NS, *P*_adj_ > 0.05; *P_adj_ ≤ 0.05; **P_adj_ ≤ 0.01; ***P_adj_ ≤ 0.001; ****P_adj_ ≤ 0.0001; the exact adjusted *P* values from left to right are as follows: *P*_adj_ = 0.148, 1.69 × 10^−25^). Horizontal lines in the violins indicate the median and the first and third quartiles. **g**, Single-locus example of the changes in CA upon p300i at a promoter with no H3K27ac loss upon p300i when measured at the endogenous (bottom-left) or ectopic (bottom-right) locus. Top, genome browser tracks of H3K27ac in DMSO and p300i condition, and DHS. Bottom, average SMF signals (1 − meth %) in control (DMSO—black line and dots) and cells treated with a p300 inhibitor (p300i—red dots and line). **h**, Single-locus example of the changes in CA upon p300i at an enhancer regulated by p300 when measured at the endogenous (bottom-left) or ectopic (bottom-right) locus. Same representation as **g**.

**Extended Data Fig. 7:**
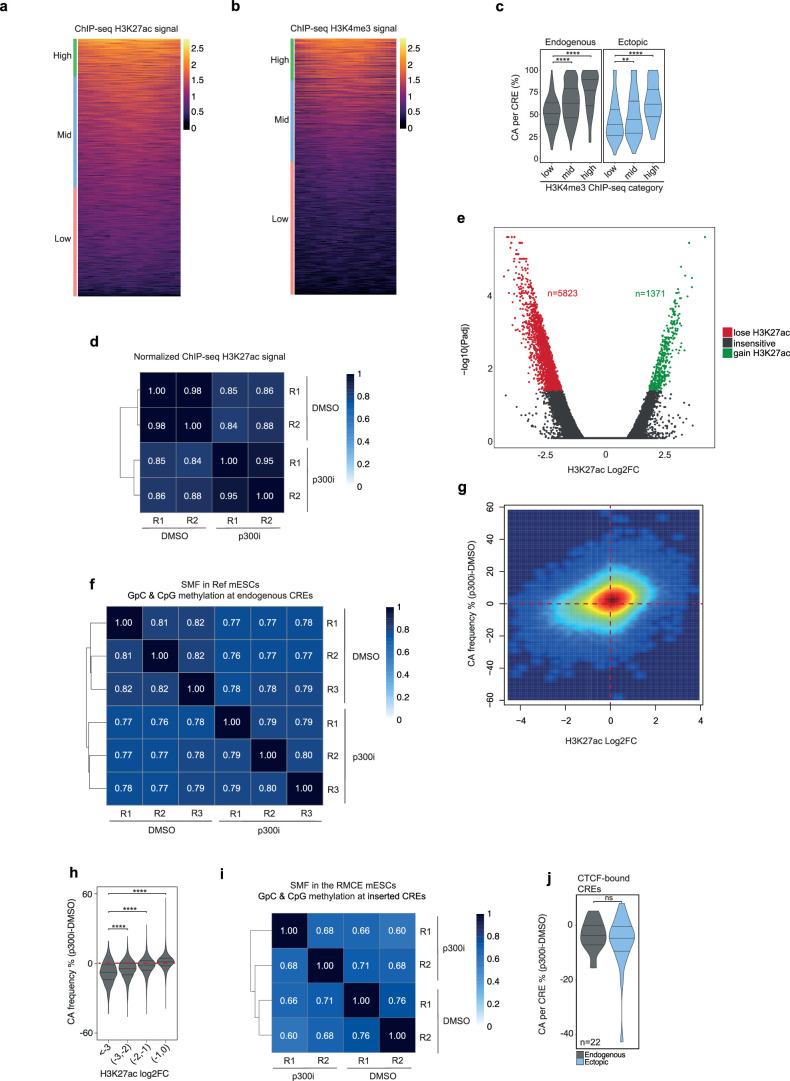
Effect of p300 inhibition on H3K27ac and chromatin accessibility. **a**, H3K27ac ChIP signal, normalized per ChIP input and spike-in ranked from high to low. The H3K27ac signal is calculated in 2 kb windows covering transcription factor binding sites (TFBSs) in the genomic regions enriched during the SMF bait capture step (Methods: TFBS annotation). Shown is the log_10_ of the H3K27ac coverage. Classes were defined based on the enrichment pattern, as: low (<100), mid (between 100 and 500) and high (>500). **b**, H3K4me3 ChIP signal, normalized per ChIP input and spike-in ranked from high to low. The H3K4me3 signal is calculated in 2 kb windows covering TFBSs in the genomic regions enriched during the SMF bait capture step (see Methods for TFBS annotation). Shown is the log_10_ of the H3K4me3 coverage. Classes were defined based on the enrichment pattern, as: low (<50), mid (between 50 and 300) and high (>300). **c**, Violin plots showing the frequency of chromatin accessibility at CREs when measured at the endogenous (black) and ectopic (blue) loci as a function of H3K4me3 ChIP–seq enrichment as measured at the endogenous loci. Asterisks indicate the significance of the two-sided Wilcoxon rank-sum test with Benjamini–Hochberg correction for multiple comparisons (n=597; NS: adjusted p > 0.05; **: adjusted p ≤ 0.01; ****: adjusted p ≤ 0.0001). For ectopic CREs, from left to right, the exact adjusted p-values are 0.009 and 3.2 × 10^−5^. For endogenous CREs, from left to right, the exact adjusted p-values are 4.58 × 10^−11^ and 3.86 × 10^−5^. Horizontal lines in the violins indicate the median and the first and third quartiles. **d**, Pearson correlation heatmap of H3K27ac ChIP–seq signal across biological replicates in DMSO or p300i treatment, normalized per input and spike-in (Supplementary Methods: ChIP-seq upon p300 inhibition pre-processing and analysis). H3K27ac signal is calculated in 2 kb windows covering the genomic regions enriched during the SMF bait capture step. The dendrogram (left) shows hierarchical clustering of H3K27ac signal, using complete-linkage method. The sample order reflects their similarity in H3K27ac signal binding, with branch lengths indicating the relative distance between clusters. The Pearson correlation coefficient R is annotated. **e**, Volcano plot showing the H3K27ac log_2_ fold change (log_2_FC, x-axis) and −log_10_ adjusted p-value (y-axis) probing the changes of H3K27ac enrichment upon p300 inhibition at endogenous loci. The log_2_ fold change of H3K27ac was calculated using the DESeq2 package, providing H3K27ac counts in DMSO or p300i treatment replicates, after normalizing per input and spike-in (Supplementary Methods: ChIP-seq upon p300 inhibition pre-processing and analysis). **f**, Pearson correlation heatmap of the methylation rates of cytosines in different contexts (that is, CpG and GpC) across biological replicates in DMSO or p300i treatment. Methylation rates were measured by SMF at the genomic regions enriched during the SMF bait capture step, in DNMT TKO mESCs (ref). Same representation as **d**. **g**, Scatterplot showing the distribution of chromatin accessibility (p300i-DMSO, y-axis) frequencies measured at endogenous loci as a function of the H3K27ac log_2_ fold change (log_2_FC, x-axis) upon p300 inhibition at the same loci. **h**, Loss of chromatin accessibility scales with the loss of H3K27ac at endogenous loci. Violin box plots showing the difference in the frequency of chromatin accessibility (CA p300i–CA DMSO) at endogenous loci as a function of H3K27ac log_2_ fold changes. Asterisks indicate the significance of two-sided Wilcoxon rank-sum tests comparing each H3K27ac log_2_FC class to the <−3 class, with Benjamini–Hochberg correction for multiple comparisons (n = 73,529; NS: adjusted p > 0.05; ****: adjusted p ≤ 0.0001). From left to right, the exact adjusted p-values for the three comparisons are 1.48 × 10^−5^, 4.43 × 10^−25^ and 4.40 × 10^−61^. Horizontal lines in the violins indicate the median and the first and third quartiles. **i**, Pearson correlation heatmap of the methylation rates of cytosines in different contexts (that is, CpG and GpC) across biological replicates in DMSO or p300i treatment. Methylation rates were measured by SMF at the genomic regions inserted at the ectopic site. Same representation as **d**. **j**, CTCF is not affected by p300 inhibition. Violin box plots showing the difference in the frequency of chromatin accessibility (CA p300i–CA DMSO) at the endogenous (black) and ectopic loci (blue) upon p300 inhibition. Asterisks indicate the significance of the two-sided paired Wilcoxon signed-rank test with Benjamini–Hochberg correction for multiple comparisons (NS: adjusted p > 0.05; the exact adjusted p-value is 0.252). Horizontal lines in the violins indicate the median and the first and third quartiles.

CRE fragments tend to adopt the chromatin state of their insertion site^41,42^. We therefore hypothesized that reduced CA at the ectopic site reflects differences in chromatin context. At endogenous loci, CA frequency scaled with levels of active marks (Fig. 6a, left, and Extended Data Fig. 7a–c). In contrast, when relating marks at the endogenous locus to CA at the ectopic site, this correlation persisted for H3K4me3 (Extended Data Fig. 7c, right), but not for H3K27ac (Fig. 6a, right). This difference is evident when comparing two NRF1-bound promoters with distinct endogenous H3K27ac levels (Fig. 6b–e). The low-acetylated *Fam227b* promoter recapitulates CA (Fig. 6b,c), while the highly acetylated *Rsrp1* promoter only partially does so (Fig. 6d,e). This suggests that recruitment of p300, the histone acetyltransferase depositing H3K27ac, may enhance CA at endogenous loci and that its effect is lost at the ectopic locus.

To test this model, we reduced H3K27ac levels by chemically inhibiting p300 (ref. ^43^; p300i), which binds a subset of enhancers in mESCs^44^ and identified 5,823 CREs losing H3K27ac (Extended Data Fig. 7d,e). Upon p300i, loss in CA scales with the reduction of acetylation (Extended Data Fig. 7f–h), suggesting that p300-mediated acetylation may increase the CA frequency at these loci. Our model further predicts that reducing p300 activity will selectively decrease accessibility at endogenous loci, whereas their ectopic counterparts in a chromatin-neutral environment will remain unchanged. We repeated the p300i on the cell lines containing hundreds of p300i-responsive and control CREs (Methods, ‘Library design and cloning’), and measured changes in CA frequency (Extended Data Fig. 7i). CREs that are insensitive to p300i have consistent CA frequency regardless of their genomic location (Fig. 6f, left, and Extended Data Fig. 7j). For example, the *Mctp2* promoter, which has low endogenous H3K27ac levels, is unaffected by p300i at either locus (Fig. 6g). In contrast, CREs that are p300i sensitive endogenously no longer lose CA when inserted at the ectopic site (Fig. 6f, right). For instance, the *Xpa* enhancer, which has high endogenous H3K27ac levels, loses ~20% accessibility upon p300i at the endogenous locus but remains unchanged at the ectopic locus (Fig. 6h). These results confirm that p300-mediated acetylation increases CA frequency at endogenous CREs, whereas ectopic inserts primarily rely on TF binding to open chromatin. Together, our data indicate that CA frequency is set by the combined activity of multiple TFs and further enhanced by p300 activity.

### CA frequency defines Pol II recruitment levels at enhancers

**Fig. 7:**
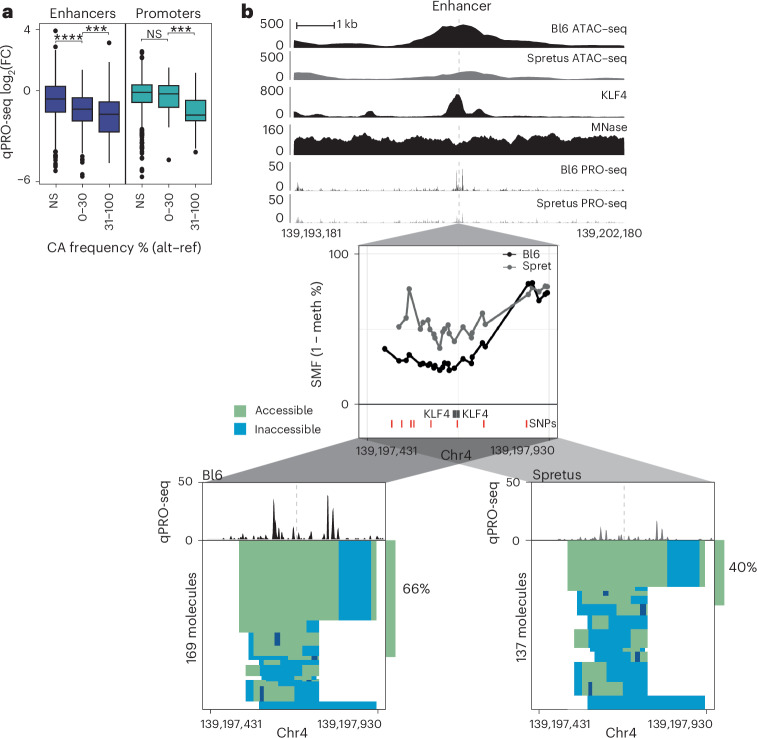
RNA Pol II activity of enhancers depends on the CA frequency. **a**, Box plots showing the distributions of qPRO-seq log_2_(FC) as a function of CA frequency reductions for TF motifs with allele-specific affinities at enhancers (left) or promoters (right). The significance of CA changes was tested over three biological replicates using Fisher’s exact test. The number of stars indicates the significance of the two-sided Wilcoxon test performed for each pair of consecutive categories (the exact *P* values from left to right are as follows: *P* = 7.1 × 10^−8^, 0.00019, 0.52, 0.00034). Boxes indicate medians and span the first to third IQR. The whiskers extend from the hinge to the largest value no further than ±1.5× IQR from the hinge. **b**, Example enhancer bound by two active KLF4 motifs, one of which has a strong difference in affinity between the Bl6 and Spret alleles. This genetic variation is associated with a 26% reduction in CA frequency in the cell population, leading to a reduction in qPRO-seq signal at the enhancer. Top, genomic tracks for BL6xSpret F1 mESCs ATAC–seq (Bl6 in black, Spretus in gray), mESCs ChIP-nexus for KLF4 and MNase-seq and BL6xSpret F1 mESCs qPRO-seq (Bl6 in black, Spretus in gray) around the TF motifs. Middle, the average SMF signal (1 − meth %) for the BL6xSpret F1 line (Bl6 in black, Spretus in gray), as well as the TF motif and SNVs annotation. Bottom, FootprintCharter stacks for the Bl6 (left) and Spretus (right) alleles, along with qPRO-seq signal at the locus. FC, fold change.

CA is an indirect proxy for enhancer activity, as many open regions are not transcriptionally active. We therefore examined how differences in CA frequency relate to transcriptional activation potential. As a proxy for enhancer activity, we measured recruitment of transcriptionally active Pol II using precision nuclear run-on sequencing (qPRO-seq)^30,45^ in both F1 lines. We compared allele-specific Pol II changes with differences in CA frequency upon TF-motif deletions. Decreases in CA frequency led to reduced Pol II levels at enhancers and promoters, with enhancer activity scaling with the magnitude of CA changes (Fig. 7a). Notably, even modest allele-specific differences in CA frequency, such as those caused by the loss of individual TF motifs, were associated with significant changes in enhancer activity (Fig. 7a,b). Together, these results indicate that CA frequency determines how often enhancers are active through recruitment of transcriptionally active Pol II.

## Discussion

Here we use SMF to dissect mechanisms controlling the frequency of CA at enhancers in cell populations. We find that CA at CREs arises from the combined activity of multiple TFs, rather than specialized individual factors. We show that CA at CREs is only partially encoded in the DNA sequence, with activating chromatin marks such as p300-mediated acetylation further increasing CA frequency.

Removing individual TFs causes a partial loss of CA^36^, in line with the fact that many TF motifs are insufficient to open chromatin in isolation^9^. In a collective, each individual TF promotes CA in ~10% cells, consistent with recent reports using KLF1-containing constructs^31^. These observations support a model in which TFs act additively to maintain CA, complementing the ‘pioneer’ model in which specific TFs initiate chromatin opening. In this sequential model, recruitment of a broader set of TFs may be facilitated by destabilized nucleosomes^46,47^. These subnucleosomal particles are in principle detectable by SMF. However, their unambiguous distinction from canonical nucleosomes requires long-read data^46,47^.

TF residence times in living cells have been measured to be on the order of seconds using single-particle tracking^48,49^ and sometimes last up to a minute for KLF1 (ref. ^31^). ATP-dependent remodeling complexes need to be constantly recruited to maintain chromatin open, as nucleosomes re-occupy DNA within minutes upon their inhibition^15,16^. Increasing the number of TF binding sites may be a mechanism to prolong the time at which CREs are occupied by TFs, and thus remain accessible. This cumulative function of short-lived TF occupancy events could maintain CA for longer periods of time. This is conceptually similar to the TF-exchange model that has been proposed to explain how transcriptional bursts exceed the residence time of TFs^50^.

Our data suggest that TF binding can occur in the absence of p300-mediated acetylation, consistent with recent findings using synthetic constructs^25^. Yet, we find that p300 activity enhances CA, consistent with reports that H3K27ac reduces the TF concentration required for SOX9 to function^51^. Our data provide a potential molecular mechanism to explain the effects of the chromatin environment on enhancer activities^52–54^. The precise mechanisms by which acetylation enhances CA remain to be identified. This could, for instance, occur through enhanced affinity of the TFs^55^, or the recruitment of bromodomain-containing chromatin-modifying enzymes^56^.

## Methods

### Ethics statement

No experiments involving human participants, human specimens, human-derived biological materials or nonpublic human data were performed in this study. Publicly available datasets were accessed and analyzed in accordance with the usage terms of the original repositories and are cited in the relevant sections.

The F1 hybrid cells Bl6xCast analyzed in this study were generated under animal protocols approved by the institutional animal welfare officer (Tierschutzbeauftragter; approval DKFZ-366). Animal work was carried out in accordance with applicable governmental and institutional animal welfare guidelines. Mice were housed at the DKFZ animal facility under controlled environmental conditions with ad libitum access to standard chow, water and environmental enrichment.

### Cell lines

A total of 159 WT mESCs^57^ and 159 DNA methyltransferase triple-knockout (DNMT TKO)^58^ were used to generate the reference SMF datasets^18^. Bl6xSpretus F1 hybrid cells were obtained from ref. ^59^. F1 hybrid cells Bl6xCast were derived at the DKFZ mouse facility (Odom lab). TC-1 mESCs carrying the RMCE landing pad were derived from the 129S6/SvEvTac genetic background and were originally obtained from A. Dean at the National Institutes of Health. All three cell lines were targeted for CRISPR-KO of the three DNMTs to generate Bl6xCast DNMT TKO and Bl6xSpret DNMT TKO and TC-1 TKO. SOX2-degron mESC line was previously derived^60^. See Supplementary Table 3 for a list of the experiments performed in this study and the corresponding cell line.

### Cell culture

mESCs were cultured on 0.2% gelatin-coated plates in ES medium (Dulbecco’s Modified Eagle Medium, supplemented with 15% FBS, 20 µg ml^−1^ leukemia inhibitory factor, 50 µM 2-mercaptoethanol, 2 mM L-glutamine and 1× nonessential amino acids) at 37 °C and 5% CO_2_. Medium was changed daily and cells were split every second day. p300 inhibition was performed by adding 3 μM A-485 (Selleck Chemicals, S8740) to the ES medium for 24 h. For conditional degradation, cells were treated for 2 h with 500 nM of dTAG (Sigma-Aldrich, SML2601-5MG).

### Generation of DNMT KO lines

*Dnmt1*/*Dnmt3a*/*Dnmt3b* TKO cells were generated in the TC-1 and F1 hybrid (Bl6xCast and Bl6xSpretus) cell lines using gRNAs that were previously described^58^. The three gRNAs targeting each DNMT gene were cloned in pX459 (Addgene, 48139) and transfected into the TC-1 and F1 hybrid (Bl6xCast and Bl6xSpretus) cell lines (DNMT1 forward, 5′-GCAGGAGGGACACAGTCATT-3′; DNMT1 reverse, 5′-AGGACTGCAACGTGCTTCTT-3′; DNMT3A forward, 5′-TTGGCACCTCCCAGGGTTC-3′; DNMT3A reverse, 5′-TCGGACAGTGAGTGGTGAGG-3′; DNMT3B forward, 5′-CCCCCAGAGTCCAGTTCTTA-3′; and DNMT3B reverse, 5′-GGAAGGACCGGGCCTGAG-3′). After puromycin selection, the pool was checked by PCR-restriction fragment length polymorphism and HpyCH4V digest for *DNMT3a*/*DNMT3b* double knockout. Subsequently, 96 colonies were picked and screened by PCR-restriction fragment length polymorphism and HpyCH4V individually. DNA triple-mutant clones were further confirmed by amplicon sequencing (primer sets DNMT targets). For the TKO clone used in this study, complete loss of DNA methylation was confirmed by DNA digestion with the DNA methylation-sensitive restriction enzyme HpaII.

### SMF

SMF was performed as previously described^18,24^. In short, cultured cells were collected using trypsin and washed twice with 1× PBS. Cells were counted and 250,000 cells were used per reaction. Cell pellets were resuspended in ice-cold lysis buffer, incubated on ice for 10 min and spun at 1,000*g* at 4 °C for 5 min. Nuclei were resuspended in 1× M.GpC buffer (NEB, M0227L). For the GpC methyltransferase treatment, freshly made GpC methyltransferase mix (1× M.GpC buffer, 300 mM sucrose, 64 μM SAM (NEB, B9003S)) and M.CviPI (NEB, M0227L) were added and incubated at 37 °C for 7.5 min. Prewarmed stop solution and proteinase K were added and incubated overnight at 55 °C. The next day, DNA was extracted using phenol-chloroform and treated with RNase A at 37 °C for 30 min. For whole-genome bisulfite sequencing library with targeted enrichment, DNA was fragmented into 300 bp fragments through sonication using Covaris model S2. For library preparation and targeted enrichment of CREs, the SureSelect XT Mouse Methyl-Seq Kit enrichment system for Illumina Multiplexed Sequencing Library protocol (Agilent Technologies, version E0, April 2018) was used as described in ref. ^24^. Bisulfite conversion was performed using the ZYMO EZ DNA Methylation-Gold Kit (Zymo, D5005) according to the manufacturer’s protocol. The bisulfite-converted library was PCR amplified and indexed using the SureSelect XT Mouse Methyl-Seq Kit (Agilent Technologies, G9651A). Prepared libraries were run on an Illumina sequencing platform using a NextSeq High 150 bp paired-end mode. For targeted SMF at the ectopic site, the extracted DNA was bisulfite converted using the Qiagen Epitect bisulfite kit as described in ref. ^24^. Bisulfite-converted DNA was amplified using KAPA HiFi Uracil+ (Roche; (95 °C, 4 min) × 1; (98 °C, 20 s; 60 °C, 15 s; 72 °C, 20 s) × 35; (75 °C, 5 min) × 1; 4 °C, hold) and purified using AMPure XP beads (0.75×, Beckman Coulter). The sequencing library was prepared using NEBNext DNA Ultra II library preparation kit and sequenced with a MiSeq 250 bp paired-end run, as described in ref. ^24^.

### Library design and cloning

To construct the DNA libraries, we selected 6,000 candidate CREs bound by TFs of interest, including pluripotency and ubiquitous TFs. Binding of these TFs to the selected CREs was confirmed using published ChIP–seq data (see Supplementary Table 1 for TFs of interest and ChIP–seq datasets and see Supplementary Table 2 for library composition). Additionally, the chosen CREs were required to contain a sufficient density of CpG and GpC dinucleotides to ensure adequate SMF resolution, while avoiding an over-representation of CpG islands. Of the 6,000 CREs, we inserted 2,199, and with sufficient coverage for FootprintCharter analysis, we retrieved 831. For enhancer-to-gene attribution, we assigned each enhancer to the nearest gene.

To construct the mutant DNA libraries, we started by generating a mutant version for each TFBS present in the wild-type (WT) CRE, whose binding was confirmed by ChIP–seq (Methods, ‘TFBS annotation’). We generated a corresponding mutant version designed to disrupt TF binding while preserving overall sequence properties. Most TFBSs were mutated by shuffling their internal sequence while maintaining local nucleotide composition, including existing CG/GC blocks, to ensure accurate SMF measurements.

Some motifs, such as those bound by KLF4, are extremely GC rich, making simple shuffling insufficient to abolish TF binding. In these cases, we replaced the TFBS with a fully randomized sequence of matching length that retained a CG/GC block. Among the randomized candidates, we selected the version with the lowest predicted affinity for the cognate TF, as estimated using position weight matrix (PWM)-based motif scoring.

Once a mutant TFBS had been generated for each position, we assembled all single and combinatorial mutant CRE variants by systematically substituting WT TFBSs with their corresponding mutants in every possible configuration. Each mutated CRE was then scanned for motif occurrences and compared to the WT sequence. We applied the following two quality-control criteria: (1) the targeted TFBS had to be fully abolished in the mutant sequence and (2) the mutant sequence could not acquire new high-affinity sites for a curated set of TFs (that is, CTCF, NRF1, REST, YY1, NFYA, OCT4, SOX2, KLF4). Each sequence was flanked at the 5′ and 3′ ends by unique bisulfite-resistant barcodes to enable the exclusion of PCR-derived chimeras.

To construct the DNA library used in the p300 inhibition experiment, we leveraged our ChIP–seq dataset generated under p300 inhibition to select 100 CREs that were sensitive and 100 that were not sensitive to the inhibition, for which endogenous SMF data were already available. We obtained 127 CREs with sufficient coverage for FootprintCharter analysis from the original library, and an additional 160 from the newly constructed ad hoc library; however, in the final analysis, we retained 234 CREs that overlap with the endogenous H3K27ac ChIP data.

The selected 250 bp DNA fragments (240 bp for mutant sequences, due to design constraints) were synthesized as individual ssDNA oligo pools by Twist Bioscience. The oligo pool was PCR amplified following the manufacturer’s instructions. The library was cloned using in-fusion seamless cloning (Takara Bio) into a receiving plasmid containing asymmetric tandem lox sites, for targeted insertion into the genome through RMCE, and priming regions for targeted SMF.

### Library insertion

We used previously described TC-1 ES cells that have a landing pad for targeted insertion using RMCE^37^. We used targeted locus amplification coupled with next-generation sequencing (Cergentis) to genotype the precise location of the landing pad. We mapped the landing pad on mouse chromosome (chr)2: 130,098,005–130,098,011 (Extended Data Fig. 6a,b) within a gene-poor region with no active transcription, no active chromatin marks and no repressive chromatin marks. For targeted insertion of the DNA libraries into the mouse genome, RMCE was used, as previously described^38^. Briefly, TC-1 DNMT TKO ES cells were selected under hygromycin (250 μg ml^−1^; Roche) for 14 days. Afterward, 50 million cells were nucleofected (Amaxa 4D-Nucleofector Core Unit, Lonza) with 300 µg DNA libraries containing plasmid and 180 µg pIC-CRE plasmid. After 2 days, cells were selected with 3 µM ganciclovir (Selleck Chemicals, S1878) for 10 days. For the mutant DNA libraries and for the DNA library used in the p300 inhibition experiment, 24 million cells were nucleofected with 75 µg DNA libraries containing plasmid and 45 µg pIC-CRE plasmid.

### Definition of SMF methylation calling windows

A total of 3,287,189 genomic tiles were designed to cover the genomic regions enriched during the SMF bait-capture step. Each genomic tile spanned 80 bp and overlapped the successive tile by 40 bp. Iterating over large numbers of methylation calling windows, even when done in parallel, results in prohibitive computation times. Therefore, we designed large methylation-calling windows that encompass multiple genomic tiles. To this end, the function ‘Create_MethylationCallingWindows’ from the ‘SingleMoleculeFootprinting’ package was used with parameters ‘fix.window.size’ set to TRUE and ‘max.window.size’ set to 50.

### Single-molecule methylation call

Single-molecule methylation calls were performed for cytosines covered by 20 reads or more using the function ‘CallContextMethylation’ from the development version of the ‘SingleMoleculeFootprinting’ Bioconductor package. Cytosines disrupted by SNVs in F1 SMF data were discarded for both alleles using the function ‘MaskSNPs’.

### Detection and quantification of footprints

We used FootprintCharter to quantify footprints^32^. For each genomic tile, the molecules covering the locus were extracted. Their binary methylation values were smoothed by computing a sliding average at every position with a window of 40 bp wide. A matrix of Euclidean distances across molecules was computed using the ‘parDist’ function of the ‘parallelDist’ R package. Unsupervised clustering was performed on the distance matrix using the *k*-medoids algorithm implemented in the ‘pam’ function of the ‘cluster’ R package. Up to 12 partitions were computed per locus, each populated by at least five molecules. Clustering was performed by pooling reads from paired samples (that is, Bl6 and Cast alleles, Bl6 and Spret alleles, untreated and 2-h SOX2 dTAG treated samples). This approach allowed direct quantification of the frequency changes of molecular states.

The bulk methylation signal for each partition was calculated and used to detect footprints as stretches of cytosines found unmethylated in at least 50% of molecules. Stretches between 12 bp and 75 bp in length were identified as TF footprints, while stretches longer than 120 bp were identified as nucleosome footprints. Footprints identified in <5 molecules were discarded. Footprints without at least one methylated cytosine on each side were discarded.

For ectopic amplicon SMF, single-molecule clustering was performed using a modified FootprintCharter pipeline in which the original requirement for fully continuous cytosine coverage was reduced in stringency—reads were retained provided their fraction of missing cytosines did not exceed a soft threshold (5%) and remained below a hard cutoff (30%). This adjustment preserves amplicon-specific variability while maintaining footprinting accuracy; all downstream smoothing, *k*-medoids clustering and footprint detection followed the original algorithm.

### Calculation of CA frequency and average width

For each TF motif, we calculated the percentage of molecules with continuous accessible stretches (that is, the absence of footprints). We differentiated accessible stretches longer than 100 bp to define active molecules. Widths of accessible stretches were averaged across molecules covering the same locus. For the analysis of the RMCE experiment and for comparing ectopic and endogenous conditions, CA for each CRE was calculated as the average CA measured at the motifs present within that CRE.

### TFBS annotation

TFBSs were annotated by scanning the reference mouse genome with PWMs for vertebrate TFs obtained from the JASPAR database^61^, queried through the JASPAR 2016 Bioconductor package^33^. PWM scanning was performed using the TFBSTools Bioconductor package^62^. Sequence matches with a score ≥10 were retained for validation. Publicly available ChIP–seq datasets were used to filter for bound motifs for the following 15 TFs: BANP, CTCF, E2F1, ESRRB, FOXD3, MAFK, MYC, NFYA, NRF1, OTX2, REST, STAT3, YY1, ZIC3 and ZFX. TFBS predictions for OCT4, SOX2 and KLF4 were obtained from ref. ^63^. For compatibility with the rest of the TFs, the relevant JASPAR PWMs were used to rescore the motif hits and validate them using the relevant ChIP-nexus datasets reported in ref. ^63^. When PWM scanning resulted in overlapping motif annotations, only the motif with the highest PWM match score was retained for further analyses.

### Number of TF motifs at CREs

The number of TF motifs at CREs was defined using the ‘Arrange_TFBSs_clusters’ function from the ‘SingleMoleculeFootprinting’ Bioconductor package. A list of bound and measured motifs was passed to the TFBSs argument. The arguments ‘max_cluster_width’ and ‘max_cluster_size’ were set to 300 and 10, respectively.

### TFBS loss of function annotation

For each TFBS, the relative change in PWM match score across alleles was computed as (A–R)/R. TFBSs were considered loss of function at the alternative allele if their relative change was lower than −0.5. Gain of function motifs (relative change > 1.5) were discarded due to the lack of ChIP–seq/ChIP-nexus data for validation. All the remaining TF motifs were considered unperturbed across alleles. In some instances, the Bl6xCast and Bl6xSpret F1s presented the very same genetic variation. To avoid such redundancies, we retained only the TFBS from the F1 cross with the highest SMF coverage.

### SMF: single-locus plots

SMF bulk plots were generated using the function ‘PlotAvgSMF’ from the ‘SingleMoleculeFootprinting’ package. FootprintCharter heatmaps were generated using custom functions. Molecules were sorted for plotting using the partitioning results from FootprintCharter.

### Data visualization and schematic illustrations

Scatterplots, box plots, violin plots, density plots, volcano plots and genomic tracks displayed throughout the manuscript were generated using the R package ggplot2. Heatmaps were generated using the ‘ComplexHeatmap’ package^64^. Schematic illustrations displayed throughout the manuscript were generated using BioRender.

### Statistics, data analysis and high-performance computing

Testing for statistically significant differences in the distributions of CA frequency values, or deltas in PRO-seq log_2_ fold changes, was carried out using the R implementation of the Wilcoxon signed-rank test.

Correlation coefficients were computed using the R function ‘cor’ with argument ‘method’ set to ‘pearson’.

All the scripting and data analysis for this project were performed using R (v4.2.2)^65^ and RStudio Pro 2023.12.0 + 369.pro3 (ref. ^66^). The preprocessing pipeline for SMF data was scripted using Nextflow (v22.10.6)^67^. All high-performance computing was performed using the SLURM workload manager^68^ either through direct bash scripting or through the R package ‘rslurm’^69^.

### Reporting summary

Further information on research design is available in the Nature Portfolio Reporting Summary linked to this article.

## Online content

Any methods, additional references, Nature Portfolio reporting summaries, source data, extended data, supplementary information, acknowledgements, peer review information; details of author contributions and competing interests; and statements of data and code availability are available at 10.1038/s41588-026-02703-x.

## Supplementary information

Supplementary Information Supplementary Methods and Supplementary Tables 2 and 3.

Reporting Summary

Peer Review File

Supplementary Table 1 Reference datasets used in this study for binding sites of 18 TFs active in mESCs.

## Source data

Source Data Extended Data Fig. 5 Unprocessed western blots for Extended Data Fig. 5c.

## Data Availability

Generated sequencing data reported in this paper have been deposited on ArrayExpress—F1 SMF, E-MTAB-14460; SOX2 dTAG SMF, E-MTAB-15014; F1 PRO-seq, E-MTAB-14459; F1 ChIP–seq, E-MTAB-16466; SMF upon p300i, E-MTAB-17191; H3K27ac ChIP–seq upon p300i, E-MTAB-14916; MCHIRA, E-MTAB-16658, E-MTAB-16659, E-MTAB-16657. Source data are provided with this paper.

## Code availability

FootprintCharter is distributed with the Bioconductor package ‘SingleMoleculeFootprinting’ at https://bioconductor.org/packages/SingleMoleculeFootprinting/.

The code used to produce the figures in this manuscript is available at https://github.com/Krebslabrep/TF-chromatin.git and

10.5281/zenodo.20649044 (ref. ^70^).
