# Supplementary material for: Cumulative transcription factor binding and p300-mediated histone acetylation drive enhancer activation frequency
Source: Nat Genet. 2026 Jul 31;58(8):1976–87. doi: 10.1038/s41588-026-02703-x (PMC13447113; doi:10.1038/s41588-026-02703-x)
Supplement: Supplementary file 1 [file 41588_2026_2703_MOESM1_ESM.pdf]

nature genetics

Supplementary information

https://doi.org/10.1038/s41588-026-02703-x

Cumulative transcription factor binding and p300-mediated histone acetylation drive enhancer activation frequency

In the format provided by the authors and unedited

Table of Contents

Supplementary Methods 2

Western blotting 2

Cross-link ChIP-seq 2

PRO-seq 3

PRO-seq data pre-processing 4

Processing of publicly available ChIP-seq and ChIP-nexus datasets 4

ChIP-seq upon p300 inhibition pre-processing and analysis 4

F1 ChIP-seq data pre-processing 5

F1 ATAC-seq data pre-processing 5

SOX2 dTAG ATAC-seq data pre-processing 5

Bait capture SMF data pre-processing 5

Targeted SMF at the ectopic locus data pre-processing 6

Annotation of enhancers and promoters 6

PRO-seq statistical testing 6

References 6

Supplementary tables 9

Supplementary Table 2. 9

Supplementary Table 3. 9

Supplementary Methods

Western blotting

Cells were harvested, washed twice in ice cold 1X PBS and resuspended in cold Lysis Buffer A (10 mM HEPES, 5mM MgCl2, 0.25M Sucrose, 0.1 % NP-40). Cytoplasmic membranes were lysed by incubating 20 min on ice and pipetting up and down several times. Nuclei were spun down at 9800g during 10 min at 4ºC and then lysed during 10 min on ice in Lysis Buffer B (2.5 mM HEPES, 1.5 mM MgCl2, 0.1 mM EDTA, 20 % Glycerol). Nuclear extracts were recovered by vortexing samples at least 20 sec at max power and by spinning at 15000g 15 min at 4ºC. Protein concentration was determined using Qubit protein quantification kit. 20μg of protein were loaded in a 10% SDS PAGE gel. Proteins were then transferred to a nitrocellulose membrane. Membranes were incubated overnight at 4ºC with antibodies against HA (1:1000, Biolegend, #901501) for Sox2 and against H3 as a loading control (1:2000, Cell signaling, #4499T).

Cross-link ChIP-seq

The protocol was adapted from[1], with minor modifications. 10 million mESCs (i.e., DNMT TKO cells for H3K27ac and Bl6xSpret F1 hybrid TKO cells for TFs) were harvested and cross-linked in 3 ml pre-tempered (25°C) ES medium containing 1% formaldehyde and supplemented with sodium butyrate (5mM final concentration) for 10 min at room temperature with rotation. The cross-linking reaction was quenched by adding glycine (125mM final concentration) and incubated for 10 min at room temperature. Cells were washed twice with ice-cold PBS containing 10% FBS and centrifuged at 200g for 5 min at 4°C. At this point, cell pellets can be snap frozen and stored at –80 °C for several months. Pellets were resuspended in 300 μl Sonication Buffer (50 mM Tris-HCl pH 8.0; 0.5% SDS) and chromatin was sheared using the Bioruptor Pico (Diagenode) for 15 cycles (30” ON/30” OFF). Sonicated lysates were diluted 1:6 in lysis buffer (10 mM Tris-HCl, pH 8.0; 100 mM NaCl; 1% Triton X-100; 1 mM EDTA; 0.5 mM EGTA; 0.1% sodium deoxycholate; and 0.5% N-lauroylsarcosine). After centrifugation at full speed for 10 min at 4 °C, the soluble fraction was collected. A 2.5% portion of the supernatant was kept as input, and the fragmentation pattern (~150–500 bp) was analyzed by agarose gel electrophoresis. At this point, sonicated lysates may be aliquoted and stored at –80 °C with a final concentration of 10% glycerol. For each immunoprecipitation (IP), 30 μl of Protein G Dynabeads (Invitrogen) were washed twice with 1 ml of PBS-T (PBS + 0.01% Tween-20) and incubated with 3 μg antibody against H3K27ac (ab4729, Abcam), 5 μg antibody against KLF4 (AF3158, R&D Systems), CTCF (07-729, Merck Millipore) or Sox2 (AF2018, R&D Systems) for 1 h at room temperature with rotation. Coated beads were washed once in cold PBS-T and twice in lysis buffer, then resuspended in 30 μl of lysis buffer per IP and added to chromatin (15 μg for H3K27ac; 10 μg for TFs). At this point, for the H3K27ac ChIP, 1 μg of exogenous chromatin (from Drosophila Schneider 2 (S2) cells, prepared with the same protocol and stored at –80 °C with a final concentration of 10% glycerol) was added to each reaction as spike-in.

After overnight incubation at 4 °C with rotation, bead-immunocomplexes were washed twice (5 min per wash), using the following buffers: RIPA, RIPA supplemented with 360 mM NaCl, and LiCl buffer (10 mM Tris-HCl, pH 8.0; 250 mM LiCl; 0.5% NP-40; 0.5% sodium deoxycholate; 1 mM EDTA). The complexes were then briefly rinsed with TE buffer and eluted in ChIP SDS elution buffer (10 mM Tris-HCl, pH 8.0; 300 mM NaCl; 5 mM EDTA; 0.5% SDS). RNA and protein digestion were performed by adding 2 μl of RNase A (10 mg/ml stock) and incubating for 30 min at 37 °C, followed by the addition of 1.5 μl of Proteinase K

(20 mg/ml stock) and incubation at 55 °C for 1 h. Cross-links were reversed by overnight incubation at 65 °C. DNA was then purified using 1.4X SPRI-select beads. Sequencing libraries were prepared using the NEBNext Ultra II DNA Library Preparation Kit (New England Biolabs) and sequenced on Aviti Cloudbreak Low 2x150 (250 M clusters/run) for H3K27ac and Aviti Cloudbreak High 2x75 (1000 M clusters/run) for the TFs.

PRO-seq

Precision run-on sequencing (PRO-seq) was performed as previously described [2,3] with slight modifications. 5 million TKO F1 mESCs were used per sample with a spike-in of 0.05 million (1%) *Drosophila* S2 cells. Two replicates per cell line were generated, with each replicate prepared simultaneously for all cell lines and conditions. Cells were harvested using Trypsin-EDTA on ice and washed twice with 10 mL ice-cold PBS, followed by centrifugation at 1000x g for 4 min at 4°C. Permeabilization was performed in 10 mL of permeabilization buffer (10 mM Tris-HCl pH 8.0, 250 mM sucrose, 10 mM KCl, 5 mM MgCl2, 1 mM EGTA, 0.1% Igepal, 0.05% Tween-20, 0.5 mM DTT, 10% (vol/vol) glycerol, 1 tablet of protease inhibitor per 50 mL, 4 units/ml SUPERaseIN inhibitor) on ice for 10 min. The cell pellets were then washed twice with 10 mL of ice-cold cell wash buffer (10 mM Tris-HCl pH 8.0, 250 mM sucrose, 10 mM KCl, 5 mM MgCl2, 1 mM EGTA, 0.5 mM DTT, 10% (vol/vol) glycerol, 1 tablet of protease inhibitor per 50 mL, 4 units/mL SUPERaseIN inhibitor) with centrifugation at 1000x g for 4 min at 4°C. Cells were resuspended in 50 µL freeze buffer (50 mM Tris-HCl, pH 8.0, 40% (vol/vol) glycerol, 5 mM MgCl2, 1.1 mM EDTA, 0.5 mM DTT and 4 units/mL SUPERaseIN inhibitor), snap-frozen, and stored at -80°C until further use. The nuclear run-on reaction was performed as a 2-biotin run-on. For this, 50 µL of the 2x Nuclear-run on master mix (10 mM Tris-Cl pH 8.0, 5 mM MgCl2, 1 mM DTT, 300 mM KCl, 40 µM Biotin-11-UTP, 40 µM Biotin-11-CTP, 40 µM ATP, 40 µM GTP, 1% Sarkosyl, 1 µL SUPERaseIN inhibitor) was prepared for each sample. The reaction mix was pre-heated at 37°C and 50 µL of the pre-calculated number of cells were added to each reaction vial, mixed thoroughly and incubated at 37°C for 5 min with shaking (750 rpm). To stop the reaction, 350 µL of the RL Buffer from the Norgen RNA extraction kit were added and vortexed. 240 µL of 100% ethanol was added to the mixture and vortexed again. RNA extraction was performed according to the kit's manual. The final RNA was eluted twice with 50 µL H2O and pooled to a final volume of 100 µL. For the base hydrolysis, the RNA was denatured for 30 seconds at 65°C and snap-cooled on ice. 25 µL of ice-cold 1N NaOH were added and incubated on ice for 10 min. RNA was precipitated by adding 125 µL Tris-HCl (pH 6.8), 5 µL NaCl, 1 µL GlycoBlue and 650 µL 100% EtOH and centrifugation at 20,000x g at 4°C for 20 min. The RNA pellet was washed with 70% EtOH, air-dried and resuspended in 6 µL H2O. For the 3' RNA adaptor ligation, 1 µL of REV3 3'RNA adaptor dilution (10 µM), heat-denatured at 65°C for 20 seconds and placed on ice, was added to the 6 µL resuspended RNA. 13 µL of the RNA ligation mix using T4 RNA ligase I was added and incubated at 25°C for 1 hour. Biotin RNA enrichment was performed by adding 55 µL binding buffer (10 mM Tris-HCL pH 7.4, 300 mM NaCl, 0.1% Tergitol, 1 mM EDTA) to each ligation reaction, followed by 25 µL of pre-washed streptavidin beads. Reactions were incubated on a rotator set to 8 rpm at room temperature for 20 min. Beads were washed once with ice-cold high-salt buffer (50 mM Tris-HCL pH 7.4, 2M NaCl, 0.5% Tergitol, 1mM EDTA), transferred to new tubes, and washed once with low-salt buffer (5 mM Tris-HCL pH 7.4, 0.1% Tergitol, 1mM EDTA). On-bead 5' hydroxyl repair was performed by resuspending the beads in 19 µL of the PNK mix and incubated at 37°C for 30 min with soft shaking (350 rpm). For the 5' cap repair reaction, the beads were resuspended in the enzyme mix containing RppH and ThermoPol Reaction Buffer and incubated at 37°C for 1 hour with soft shaking (350 rpm). On-bead 5' RNA adaptor ligation was performed by

resuspending the beads in 7 µL of REV5 5'RNA adaptor dilution (6 µL of H2O + 1 µL of 10 µM REV5), heat-denatured at 65°C for 30 seconds and placed on ice. 12 µL of the RNA ligation mix using T4 RNA ligase was added and incubated at 25°C for 1 hour. Beads were washed once with ice-cold high-salt buffer, transferred to new tubes, and washed once with low-salt buffer. The RNA was cleaned-up using Trizol and chloroform. The RNA was reverse transcribed using the Maxima H minus RT enzyme and the RP1 reverse-transcription primer. The correct number of PCR cycles was determined by test PCR and Bioanalyzer analysis. In the end, the final PCR was performed using 13 cycles. The amplified library was cleaned-up using magnetic SPRI beads at a ratio of 1.8x and further size selected with a SPRI beads ratio of 1x to remove primer dimers. The library was run on a NextSeq 2000 P3 with 50 bp single-end sequencing.

PRO-seq data pre-processing

For the F1 PRO-seq data generated in this study, raw sequencing reads were trimmed using Cutadapt (version 3.5)[4]. UMIs were detected and extracted using UMI-tools (version 1.1.2) [5]. To assign pre-processed reads to the correct allele of origin, SNVs annotation between the species of interest were fetched from the Mouse Genome Project portal[6]. SNVs were injected into the UCSC *Mus musculus* reference genome distributed through the Bioconductor [7,8] package BSgenome.Mmusculus.UCSC.mm10[9] using the function *qAlign* provided with the Bioconductor package *QuasR*[10]. This resulted in two separate genomes to which reads were competitively aligned using the *QuasR* function *qAlign* with default alignment parameters[10].

Aligned reads were deduplicated using UMI-tools. Reads aligned to rRNAs were removed.

Processing of publicly available ChIP-seq and ChIP-nexus datasets

Publicly available ChIP-seq and ChIP-nexus datasets were pre-processed using the nf-core pipeline nf-core/chipseq v.2.0.0 with arguments "--narrow_peak --read_length 50" [11]. For our H3K27ac and H3K4me3 ChIP-seq analysis, we calculated the coverage within 2kb windows using the *qCount* function of the *QuasR* package, focusing on Transcription Factor Binding Sites (TFBS) in genomic regions enriched during the SMF bait capture step. Based on the enrichment patterns of H3K27ac and H3K4me3, we categorized the regions into three classes: low, mid, and high enrichment. For H3K27ac classes are as follows: low (<100), mid (between 100 and 500) and high (>500). For H3K4me3 classes are as follows: low (<50), mid (between 50 and 300) and high (>300). See Extended Data Fig. 7a and 7b.

ChIP-seq upon p300 inhibition pre-processing and analysis

Illumina adaptors and low quality bases were trimmed using TrimGalore (v 0.6.7). Pre-processed reads were aligned to the mouse reference genome (BSgenome.Mmusculus.UCSC.mm10) using the R package *QuasR*, which uses *Bowtie* as aligner (alignment parameter: -m 1 -e 70 -X 1000 -k 2 -best -strata). Only uniquely aligned reads were kept and deduplicated using the tool *MarkDuplicates* from *Picard* (v 3.1.0). Normalized signal coverage bigwig files were obtained with *deepTools* bamCoverage (v 3.5.0) and a bin size of 50 bases. The H3K27ac ChIP-seq contained exogenous spike-in, for which reads were aligned independently to the Drosophila reference genome (BSgenome.Dmelanogaster.UCSC.dm6), and processed as described for the reads aligned to the mouse genome. The number of uniquely mapped reads aligning to major mouse and fly chromosomes, in both IP and input samples, was obtained and used to compute normalization

factors (using the formula: aligned reads * (target IP/spike-in IP) * (spike-in input/target input) [12]). The normalization factors were then applied with deepTools bamCoverage to generate scaled bigWig files.

The log2 fold change of H3K27ac and statistical testing were computed using the DESeq2 Bioconductor package[13], providing H3K27ac counts (calculated using the *qCount* function of the *QuasR* package, in 2kb windows covering the genomic regions enriched during the SMF bait capture step) in DMSO or p300i treatment replicates, after applying the normalization factors. The p-values were adjusted using the Benjamini-Hochberg method.

F1 ChIP-seq data pre-processing

AVITI and Illumina adapters were trimmed using TrimGalore! v0.6.7[14]. Trimmed reads were competitively aligned to the Bl6 and Spret genomes and filtered to discard reads with allelic mapping bias using a version of WASP extended to include indels[15,16]. Finally, duplicated reads were identified and discarded using the Picard tool MarkDuplicates v2.15.0[20]

F1 ATAC-seq data pre-processing

Publicly available ATAC-seq data produced using an analogous Bl6xCast F1 ESC line [17] and an analogous Bl6xSpret F1 ESC line[18] were fetched using the nf-core pipeline nf-core/fetchngs [11,19]. Sequencing reads were trimmed for Illumina adapters and low-quality bases using TrimGalore! v0.6.7 (Krueger et al., 2023).

Reads shorter than 20bp were discarded after trimming. To assign pre-processed reads to the correct allele of origin, SNVs annotations between mouse species were fetched from the Mouse Genome Project portal[6]. SNVs were injected into the UCSC *Mus musculus* reference genome distributed through the Bioconductor[7,8] package BSgenome.Mmusculus.UCSC.mm10[9] using the function *qAlign* provided with the Bioconductor package *QuasR*[10]. This resulted in two separate genomes to which reads were competitively aligned using the *QuasR* function *qAlign* with alignment parameters "-e 70 -X 1000 -k 2 -best -strata". The best alignment determined the allelic assignment for each read. Finally, duplicated reads were identified and discarded using the Picard tool MarkDuplicates v2.15.0[20].

SOX2 dTAG ATAC-seq data pre-processing

Publicly available SOX2 dTAG ATAC-seq data[21] was fetched using the nf-core pipeline nf-core/fetchngs and pre-processed using the nf-core pipeline nf-core/atacseq v.2.0 with arguments --read_length 150"[11,19]. We calculated the coverage within 500bp windows centered around bound SOX2 motifs using the *qCount* function of the *QuasR* package. We normalized the counts by sequencing depth across conditions by computing the counts per million (cpm).

Bait capture SMF data pre-processing

Bait capture SMF sequencing reads were trimmed for Illumina adapters and low-quality bases using *TrimGalore* v0.6.7. Reads shorter than 20bp after trimming were discarded.

For F1 SMF data, to assign pre-processed reads to the correct allele of origin, SNVs annotations between mouse species were fetched from the Mouse Genome Project portal[6]. SNVs were injected into the UCSC *Mus musculus* reference genome distributed through the Bioconductor [7,8] package BSgenome.Mmusculus.UCSC.mm10[9] using the function *qAlign* provided with the Bioconductor package *QuasR*[10]. This resulted in two separate genomes to

which reads were competitively aligned using the *QuasR* function *qAlign* with alignment parameters "-e 70 -X 1000 -k 2 -best -strata". The best alignment determined the allelic assignment for each read. Finally, duplicated reads were identified and discarded using the Picard tool MarkDuplicates v2.15.0[20].

Targeted SMF at the ectopic locus data pre-processing

Amplicon SMF sequencing reads were trimmed for Illumina adapters and low-quality bases using *TrimGalore* v0.6.7. Reads shorter than 20 bp after trimming were discarded. A custom R script was then used to remove the plasmid backbone from the reads. For libraries containing wild-type fragments only, pre-processed reads were mapped to the bisulfite-indexed *Mus musculus* reference genome using the *QuasR* function *qAlign* with alignment parameters "-e 70 -X 1000 -k 2 -best -strata". For the mutant libraries, pre-processed reads were mapped to a bisulfite-indexed custom BSgenome using the same method. Duplicate reads were identified based on the cytosine methylation pattern and removed using custom functions.

Annotation of enhancers and promoters

We classified bound TFBSs as a function of the type of CRE they are bound to. We used an existing annotation based on 12-state chromHMM to classify CREs as promoters or enhancers[22]. For simplicity, we selected and pooled states as CTCF (1_Insulator), intergenic (2_Intergenic), enhancer (4_Enhancer, 8_StrongEnhancer, 11_WeakEnhancer), promoter (7_ActivePromoter). Of note, this classification does not simply discriminate CREs based on TF identity, therefore the enhancer category includes 2,200 CTCF-bound loci. We then analysed chromatin accessibility at all enhancers and promoters covered in the bait-capture data Agilent (#931052). These baits cover 59.7% of open regions as detected by DNase sequencing, covering all annotated promoters and a majority of the enhancers active in mESCs [23,24].

PRO-seq statistical testing

For each TF motif and genotype, the PRO-seq signal covering the 200 bp surrounding the motif was quantified using the *qCount* function of the *QuasR* package. Statistical testing was performed using the *DESeq2* Bioconductor package [13].

References

1. Trovato, M. *et al.* Histone H3.3 lysine 9 and 27 control repressive chromatin at cryptic enhancers and bivalent promoters. *Nat Commun* **15**, 7557 (2024).

2. Judd, J. *et al. A Rapid, Sensitive, Scalable Method for Precision Run-On Sequencing (PRO-Seq)*. http://biorxiv.org/lookup/doi/10.1101/2020.05.18.102277 (2020) doi:10.1101/2020.05.18.102277.

3. Kwak, H., Fuda, N. J., Core, L. J. & Lis, J. T. Precise Maps of RNA Polymerase Reveal How Promoters Direct Initiation and Pausing. *Science* **339**, 950–953 (2013).
4. Martin, M. Cutadapt removes adapter sequences from high-throughput sequencing reads. *EMBnet.journal; Vol 17, No 1: Next Generation Sequencing Data Analysis; DOI: https://doi.org/10.14806/ej.17.1.200* https://journal.embnet.org/index.php/embnetjournal/article/view/200 (2011).
5. Smith, T., Heger, A. & Sudbery, I. UMI-tools: modeling sequencing errors in Unique Molecular Identifiers to improve quantification accuracy. *Genome Res.* **27**, 491–499 (2017).
6. Keane, T. M. *et al.* Mouse genomic variation and its effect on phenotypes and gene regulation. *Nature* **477**, 289–294 (2011).
7. Gentleman, R. C. *et al.* Bioconductor: open software development for computational biology and bioinformatics. *Genome Biol* **5**, R80 (2004).
8. Huber, W. *et al.* Orchestrating high-throughput genomic analysis with Bioconductor. *Nat Methods* **12**, 115–121 (2015).
9. Team, T. B. D. BSgenome.Mmusculus.UCSC.mm10. https://doi.org/10.18129/B9.BIOC.BSGENOME.MMUSCULUS.UCSC.MM10 (2017).
10. Gaidatzis, D., Lerch, A., Hahne, F. & Stadler, M. B. QuasR: quantification and annotation of short reads in R. *Bioinformatics* **31**, 1130–1132 (2015).
11. Ewels, P. A. *et al.* The nf-core framework for community-curated bioinformatics pipelines. *Nat Biotechnol* **38**, 276–278 (2020).
12. Patel, L. A., Cao, Y., Mendenhall, E. M., Benner, C. & Goren, A. The Wild West of spike-in normalization. *Nat Biotechnol* **42**, 1343–1349 (2024).
13. Love, M. I., Huber, W. & Anders, S. Moderated estimation of fold change and dispersion for RNA-seq data with DESeq2. *Genome Biol* **15**, 550 (2014).

14. Krueger, F. *et al.* FelixKrueger/TrimGalore: v0.6.10 - add default decompression path. Zenodo https://doi.org/10.5281/zenodo.7598955 (2023).
15. Sigalova, O. M. *et al.* Integrating genetic variation with deep learning provides context for variants impacting transcription factor binding during embryogenesis. *Genome Res.* **35**, 1138–1153 (2025).
16. van de Geijn, B., McVicker, G., Gilad, Y. & Pritchard, J. K. WASP: allele-specific software for robust molecular quantitative trait locus discovery. *Nat Methods* **12**, 1061–1063 (2015).
17. Xu, J. *et al.* Landscape of monoallelic DNA accessibility in mouse embryonic stem cells and neural progenitor cells. *Nat Genet* **49**, 377–386 (2017).
18. Liang, W. *et al.* Systematic Analysis of Monoallelic Gene Expression and Chromatin Accessibility Across Multiple Tissues in Hybrid Mice. *Front. Cell Dev. Biol.* **9**, (2021).
19. Patel, H. *et al.* nf-core/fetchngs: nf-core/fetchngs v1.12.0 - Titanium Platypus. Zenodo https://doi.org/10.5281/zenodo.10728509 (2024).
20. Picard Tools.
21. Maresca, M. *et al.* Pioneer activity distinguishes activating from non-activating SOX2 binding sites. *The EMBO Journal* **42**, e113150 (2023).
22. Pintacuda, G. *et al.* hnRNPK Recruits PCGF3/5-PRC1 to the Xist RNA B-Repeat to Establish Polycomb-Mediated Chromosomal Silencing. *Molecular Cell* **68**, 955-969.e10 (2017).
23. Kreibich, E., Kleinendorst, R., Barzaghi, G., Kaspar, S. & Krebs, A. R. Single-molecule footprinting identifies context-dependent regulation of enhancers by DNA methylation. *Mol Cell* S1097-2765(23)00040–0 (2023) doi:10.1016/j.molcel.2023.01.017.
24. Sönmezer, C. *et al.* Molecular Co-occupancy Identifies Transcription Factor Binding Cooperativity In Vivo. *Mol Cell* **81**, 255-267.e6 (2021).

Supplementary tables

CRE_type	n
bivalent_promoter	34
enhancer	274
heterochromatin	4
intergenic	11
promoter	200
repressed	18
transcription	17
CTCF	219
ambiguous	42
N.A.	12

Supplementary Table 2.
Composition of fragments with sufficient coverage retrieved from the library inserted at the ectopic locus.

Assay	Cell line
SMF-bait capture	F1 mESCs; SOX2-degron mESCs; 159 DNMT TKO mESCs (DMSO,p300 inhibition)
PRO-seq	F1 mESCs
targeted-SMF	TC-1 DNMT TKO mESCs (RMCE) (untreated, DMSO, p300 inhibition)
ChIP-seq	159 DNMT TKO mESCs (DMSO, p300 inhibition); F1 mESC

Supplementary Table 3.
Summary of the assays performed and the corresponding cell lines.
