# Supplementary material for: Cumulative transcription factor binding and p300-mediated histone acetylation drive enhancer activation frequency
Source: Nat Genet. 2026 Jul 31;58(8):1976–87. doi: 10.1038/s41588-026-02703-x (PMC13447113; doi:10.1038/s41588-026-02703-x)
Supplement: Supplementary file 2 [file 41588_2026_2703_MOESM2_ESM.pdf]

nature portfolio

Corresponding author(s): Arnaud R Krebs, Judith B Zaugg

Last updated by author(s): Jun 3, 2026

Reporting Summary

Nature Portfolio wishes to improve the reproducibility of the work that we publish. This form provides structure for consistency and transparency in reporting. For further information on Nature Portfolio policies, see our Editorial Policies and the Editorial Policy Checklist.

Statistics

For all statistical analyses, confirm that the following items are present in the figure legend, table legend, main text, or Methods section.

n/a	Confirmed	
☐	☒	The exact sample size (*n*) for each experimental group/condition, given as a discrete number and unit of measurement
☐	☒	A statement on whether measurements were taken from distinct samples or whether the same sample was measured repeatedly
☐	☒	The statistical test(s) used AND whether they are one- or two-sided<br>*Only common tests should be described solely by name; describe more complex techniques in the Methods section.*
☐	☒	A description of all covariates tested
☐	☒	A description of any assumptions or corrections, such as tests of normality and adjustment for multiple comparisons
☐	☒	A full description of the statistical parameters including central tendency (e.g. means) or other basic estimates (e.g. regression coefficient) AND variation (e.g. standard deviation) or associated estimates of uncertainty (e.g. confidence intervals)
☒	☐	For null hypothesis testing, the test statistic (e.g. *F*, *t*, *r*) with confidence intervals, effect sizes, degrees of freedom and *P* value noted<br>*Give P values as exact values whenever suitable.*
☒	☐	For Bayesian analysis, information on the choice of priors and Markov chain Monte Carlo settings
☒	☐	For hierarchical and complex designs, identification of the appropriate level for tests and full reporting of outcomes
☐	☒	Estimates of effect sizes (e.g. Cohen's *d*, Pearson's *r*), indicating how they were calculated

Our web collection on statistics for biologists contains articles on many of the points above.

Software and code

Policy information about availability of computer code

Data collection	FootprintCharter is distributed with the devel version of Bioconductor package SingleMoleculeFootprinting at 10.18129/B9.bioc.SingleMoleculeFootprinting.
Data analysis	The code used to produce the figures in this manuscript is available at https://github.com/Krebslabrep/TF-chromatin.git.<br>All the scripting and data analysis for this project was performed using R-4.2.2 (R Core Team, 2021) and RStudio Pro 2023.12.0+369.pro3 (RStudio Team, 2020). A pre-processing pipeline for SMF data was scripted using Nextflow-22.10.6 (Di Tommaso et al., 2017). All high-performance computing was performed using the SLURM workload manager (Yoo et al., 2003) either through direct bash scripting or through the R package rslurm (Read et al., 2021).

For manuscripts utilizing custom algorithms or software that are central to the research but not yet described in published literature, software must be made available to editors and reviewers. We strongly encourage code deposition in a community repository (e.g. GitHub). See the Nature Portfolio guidelines for submitting code & software for further information.

Data

Policy information about availability of data

All manuscripts must include a data availability statement. This statement should provide the following information, where applicable:

- Accession codes, unique identifiers, or web links for publicly available datasets
- A description of any restrictions on data availability
- For clinical datasets or third party data, please ensure that the statement adheres to our policy

Generated sequencing data reported in this paper has been deposited on ArrayExpress.

Research involving human participants, their data, or biological material

Policy information about studies with human participants or human data. See also policy information about sex, gender (identity/presentation), and sexual orientation and race, ethnicity and racism.

Reporting on sex and gender	*Use the terms sex (biological attribute) and gender (shaped by social and cultural circumstances) carefully in order to avoid confusing both terms. Indicate if findings apply to only one sex or gender; describe whether sex and gender were considered in study design; whether sex and/or gender was determined based on self-reporting or assigned and methods used. Provide in the source data disaggregated sex and gender data, where this information has been collected, and if consent has been obtained for sharing of individual-level data; provide overall numbers in this Reporting Summary. Please state if this information has not been collected. Report sex- and gender-based analyses where performed, justify reasons for lack of sex- and gender-based analysis.*
Reporting on race, ethnicity, or other socially relevant groupings	*Please specify the socially constructed or socially relevant categorization variable(s) used in your manuscript and explain why they were used. Please note that such variables should not be used as proxies for other socially constructed/relevant variables (for example, race or ethnicity should not be used as a proxy for socioeconomic status). Provide clear definitions of the relevant terms used, how they were provided (by the participants/respondents, the researchers, or third parties), and the method(s) used to classify people into the different categories (e.g. self-report, census or administrative data, social media data, etc.) Please provide details about how you controlled for confounding variables in your analyses.*
Population characteristics	*Describe the covariate-relevant population characteristics of the human research participants (e.g. age, genotypic information, past and current diagnosis and treatment categories). If you filled out the behavioural & social sciences study design questions and have nothing to add here, write "See above."*
Recruitment	*Describe how participants were recruited. Outline any potential self-selection bias or other biases that may be present and how these are likely to impact results.*
Ethics oversight	*Identify the organization(s) that approved the study protocol.*

Note that full information on the approval of the study protocol must also be provided in the manuscript.

Field-specific reporting

Please select the one below that is the best fit for your research. If you are not sure, read the appropriate sections before making your selection.

☒ Life sciences ☐ Behavioural & social sciences ☐ Ecological, evolutionary & environmental sciences

For a reference copy of the document with all sections, see nature.com/documents/nr-reporting-summary-flat.pdf

Life sciences study design

All studies must disclose on these points even when the disclosure is negative.

Sample size	Not applicable
Data exclusions	Not applicable
Replication	Not applicable
Randomization	Not applicable
Blinding	Not applicable

Reporting for specific materials, systems and methods

We require information from authors about some types of materials, experimental systems and methods used in many studies. Here, indicate whether each material, system or method listed is relevant to your study. If you are not sure if a list item applies to your research, read the appropriate section before selecting a response.

Materials & experimental systems

n/a	Involved in the study
☐	☒ Antibodies
☐	☒ Eukaryotic cell lines
☐	☐ Palaeontology and archaeology
☐	☐ Animals and other organisms
☐	☐ Clinical data
☐	☐ Dual use research of concern
☐	☐ Plants

Methods

n/a	Involved in the study
☐	☒ ChIP-seq
☐	☐ Flow cytometry
☐	☐ MRI-based neuroimaging

Antibodies

Antibodies used	anti-HA Biolegend, #901501; anti-H3 Cell signaling, #4499T; anti-H3K27Ac ab4729, Abcam; anti-KLF4 R&D System, #AF3158; anti-CTCF Merck Millipore, #07-729; anti-SOX2, R&D Systems, #AF2018
Validation	anti-HA: Use of gel electrophoresis to separate proteins by size, followed by detection with an antibody. Western blot of Clone 16B12 available here https://www.biolegend.com/en-us/products/purified-anti-ha-11-epitope-tag-antibody-11374. 636 product citations. anti-H3: Use of gel electrophoresis to separate proteins by size, followed by detection with an antibody. Western blot available here https://www.cellsignal.com/products/primary-antibodies/histone-h3-d1h2-xp-rabbit-mab/4499. 1862 product citations. anti-H3K27Ac: “ChIP was performed with human thyroid cancer cell lysate and 1 µg/µg of ab4729. Lysates were incubated with the primary antibody for 16 hours at 4°C. positive control promoter: GAPDH promoter; positive control enhancer: enhancer region of a published target (10.1093/nar/gkx802); negative control: published negative region (10.1093/nar/gkx802).” Relative figure here: https://www.abcam.com/en-us/products/primary-antibodies/histone-h3-acetyl-k27-antibody-chip-grade-ab4729#overlay=images. anti-KLF4: Mouse KLF4 detection by ChIP is supported indirectly by published SIM2 co-occupancy and co-immunoprecipitation data showing SIM2 association with key pluripotency factors, including KLF4, in mouse embryonic stem cells. Relative figure here: https://www.rndsystems.com/products/mouse-klf4-antibody_af3158?gclsrc=aw.ds&gad_source=1&gad_campaignid=20161432922&gclid=Cj0KCQjwof_QBhCgARIsADaMzOf6msto7f0Z1ZtT7zJXvpfX6qhnUgvDUx1xPGuYOFLffKb6G86BAtAaArjGEALw_wcB. anti-CTCF: “WB: HeLa nuclear extract was resolved by electrophoresis, transferred to nitrocellulose and probed with anti-CTCF (1:2000 dilution). Arrow indicates CTCF (~120kDa).” Relative figure here: https://www.merckmillipore.com/DE/de/product/Anti-CTCF-Antibody,MM_NF-07-729?ReferrerURL=https%3A%2F%2Fwww.google.com%2F. anti-SOX2: SOX2-regulated gene detection by ChIP was internally validated in BG01V human embryonic stem cells using R&D Systems SOX2 antibody AF2018. The assay immunoprecipitated SOX2/DNA complexes and confirmed enrichment at the NANOG promoter by standard PCR versus a control antibody. Relative figure here: https://www.rndsystems.com/products/human-mouse-rat-sox2-antibody_af2018?gclsrc=aw.ds&gad_source=1&gad_campaignid=22313265578&gclid=Cj0KCQjwof_QBhCgARIsADaMzOei9M9RqY-mqTRRPxNXqSmebCPJUUklkXswjV3d8C-p8cbIB31figwaAq1vEALw_wcB.

Eukaryotic cell lines

Policy information about cell lines and Sex and Gender in Research

Cell line source(s)	F1 hybrid cells Bl6xCast were derived at the DKFZ mouse facility (Odom lab). Bl6xSpretus F1 hybrid cells (Hochepied et al., 2004). TC-1 mESCs (background 129S6/SvEvTac, originally obtained from A. Dean at the National Institutes of Health). Sox2 degron mESCs (Liu et al., 2021). 159 DNMT TKO mESCs (Domcke et al., 2015). In the Krebs lab, Bl6xCast, Bl6xSpret and TC-1 cell lines were targeted for CRISPR-KO of the three DNA methyl transferases to generate Bl6xCast DNMT TKO and Bl6xSpret DNMT TKO and TC-1 TKO.
Authentication	For the cell lines targeted in this study for CRISPR-KO of the three DNA methyl transferases, the complete loss of DNA methylation was confirmed by DNA digestion with the DNA methylation-sensitive restriction enzyme HpaII. For the TC-1 cell line, targeted locus amplification coupled with next generation sequencing (Cergentis) was used to genotype the precise location of the landing pad.
Mycoplasma contamination	Not applicable
Commonly misidentified lines (See ICLAC register)	*Name any commonly misidentified cell lines used in the study and provide a rationale for their use.*

Palaeontology and Archaeology

Specimen provenance	*Provide provenance information for specimens and describe permits that were obtained for the work (including the name of the issuing authority, the date of issue, and any identifying information). Permits should encompass collection and, where applicable, export.*

Specimen deposition	*Indicate where the specimens have been deposited to permit free access by other researchers.*
Dating methods	*If new dates are provided, describe how they were obtained (e.g. collection, storage, sample pretreatment and measurement), where they were obtained (i.e. lab name), the calibration program and the protocol for quality assurance OR state that no new dates are provided.*

☐ Tick this box to confirm that the raw and calibrated dates are available in the paper or in Supplementary Information.

Ethics oversight	*Identify the organization(s) that approved or provided guidance on the study protocol, OR state that no ethical approval or guidance was required and explain why not.*

Note that full information on the approval of the study protocol must also be provided in the manuscript.

Animals and other research organisms

Policy information about studies involving animals; ARRIVE guidelines recommended for reporting animal research, and Sex and Gender in Research

Laboratory animals	*For laboratory animals, report species, strain and age OR state that the study did not involve laboratory animals.*
Wild animals	*Provide details on animals observed in or captured in the field; report species and age where possible. Describe how animals were caught and transported and what happened to captive animals after the study (if killed, explain why and describe method; if released, say where and when) OR state that the study did not involve wild animals.*
Reporting on sex	*Indicate if findings apply to only one sex; describe whether sex was considered in study design, methods used for assigning sex. Provide data disaggregated for sex where this information has been collected in the source data as appropriate; provide overall numbers in this Reporting Summary. Please state if this information has not been collected. Report sex-based analyses where performed, justify reasons for lack of sex-based analysis.*
Field-collected samples	*For laboratory work with field-collected samples, describe all relevant parameters such as housing, maintenance, temperature, photoperiod and end-of-experiment protocol OR state that the study did not involve samples collected from the field.*
Ethics oversight	*Identify the organization(s) that approved or provided guidance on the study protocol, OR state that no ethical approval or guidance was required and explain why not.*

Note that full information on the approval of the study protocol must also be provided in the manuscript.

Clinical data

Policy information about clinical studies

All manuscripts should comply with the ICMJE guidelines for publication of clinical research and a completed CONSORT checklist must be included with all submissions.

Clinical trial registration	*Provide the trial registration number from ClinicalTrials.gov or an equivalent agency.*
Study protocol	*Note where the full trial protocol can be accessed OR if not available, explain why.*
Data collection	*Describe the settings and locales of data collection, noting the time periods of recruitment and data collection.*
Outcomes	*Describe how you pre-defined primary and secondary outcome measures and how you assessed these measures.*

Dual use research of concern

Policy information about dual use research of concern

Hazards

Could the accidental, deliberate or reckless misuse of agents or technologies generated in the work, or the application of information presented in the manuscript, pose a threat to:

No	Yes	
☐	☐	Public health
☐	☐	National security
☐	☐	Crops and/or livestock
☐	☐	Ecosystems
☐	☐	Any other significant area

Experiments of concern

Does the work involve any of these experiments of concern:

No	Yes	
☐	☐	Demonstrate how to render a vaccine ineffective
☐	☐	Confer resistance to therapeutically useful antibiotics or antiviral agents
☐	☐	Enhance the virulence of a pathogen or render a nonpathogen virulent
☐	☐	Increase transmissibility of a pathogen
☐	☐	Alter the host range of a pathogen
☐	☐	Enable evasion of diagnostic/detection modalities
☐	☐	Enable the weaponization of a biological agent or toxin
☐	☐	Any other potentially harmful combination of experiments and agents

Plants

Seed stocks	*Report on the source of all seed stocks or other plant material used. If applicable, state the seed stock centre and catalogue number. If plant specimens were collected from the field, describe the collection location, date and sampling procedures.*
Novel plant genotypes	*Describe the methods by which all novel plant genotypes were produced. This includes those generated by transgenic approaches, gene editing, chemical/radiation-based mutagenesis and hybridization. For transgenic lines, describe the transformation method, the number of independent lines analyzed and the generation upon which experiments were performed. For gene-edited lines, describe the editor used, the endogenous sequence targeted for editing, the targeting guide RNA sequence (if applicable) and how the editor was applied.*
Authentication	*Describe any authentication procedures for each seed stock used or novel genotype generated. Describe any experiments used to assess the effect of a mutation and, where applicable, how potential secondary effects (e.g. second site T-DNA insertions, mosiacism, off-target gene editing) were examined.*

ChIP-seq

Data deposition

☒ Confirm that both raw and final processed data have been deposited in a public database such as GEO.

☐ Confirm that you have deposited or provided access to graph files (e.g. BED files) for the called peaks.

Data access links<br>*May remain private before publication.*	https://www.ebi.ac.uk/biostudies/arrayexpress/studies/E-MTAB-14916<br>https://www.ebi.ac.uk/biostudies/arrayexpress/studies/E-MTAB-16466
Files in database submission	ChIP_H3K27Ac_A485_R2, input_H3K27Ac_DMSO_R1, ChIP_H3K27Ac_DMSO_R2, input_H3K27Ac_A485_R1, input_H3K27Ac_DMSO_R2, ChIP_H3K27Ac_DMSO_R1, input_H3K27Ac_p300i_R2, ChIP_H3K27Ac_p300i_R1. F1mESC_ChIP_CTCF_R1, F1mESC_ChIP_CTCF_R2, F1mESC_ChIP_Input_R1, F1mESC_ChIP_Input_R2, F1mESC_ChIP_KLF4_R1, F1mESC_ChIP_KLF4_R2, F1mESC_ChIP_SOX2_R1, F1mESC_ChIP_SOX2_R2
Genome browser session<br>(e.g. UCSC)	*Provide a link to an anonymized genome browser session for "Initial submission" and "Revised version" documents only, to enable peer review. Write "no longer applicable" for "Final submission" documents.*

Methodology

Replicates	Two biological replicates were performed for both DMSO and p300 inhibition (p300i) treatment (with A-485, small molecule). Agreement between the DMSO replicates is R=0.98, agreement between the p300i replicates is R=0.95.<br>Two biological replicates were performed for each of the F1mESC datasets
Sequencing depth	Sequencing libraries were prepared using the NEBNext Ultra II DNA Library Preparation Kit (New England Biolabs) and sequenced on Aviti Cloudbreak Low 2x150 (250 M clusters/run). Alignment was performed using read1. Total number of reads: DMSO_R1=4.2e+07, DMSO_R2=8.86e+07, P300i_R1=3.13e+07, p300i_R2=5.92e+07, CTCF_r1 = 1.48e+08, CTCF_r2=9.89e+07, input_r1=1.27e+08, input_r2=9.16e+07, Klf4_r1=1.12e+08, Klf4_r2=1.12e+08, Sox2_r1=1.21+e+08, Sox2_r2=5.99+e07. Uniquely mapped reads: DMSO_R1=1.01e+06, DMSO_R2=1.08e+06, P300i_R1=1.02e+06, p300i_R2=1.11e+06, CTCF_r1=1.01+e=06, CTCF_r2=1.03e+06, input_r1=1.01e+06, input_r2=1.01e+06, Klf4_r1=1e+06, Klf4_r2=1.02e+06, Sox2_r1=1.01e+06, Sox2_r2=1.02e+06. Total number of reads input: DMSO_R1=9.63e+07, DMSO_R2=6.07e+07, P300i_R1=7.82e+07, p300i_R2=7.89e+07. Uniquely mapped reads input: DMSO_R1=total=1.07e+06, DMSO_R2=1.01e+06, P300i_R1=1.06e+06, p300i_R2=1.02e+06.
Antibodies	anti-H3K27Ac Abcam, ab4729. anti-CTCF - 07-729, Merck Millipore. anti-KLF4 - AF3158, R&D Systems. anti-SOX2 - AF2018, R&D Systems
Peak calling parameters	Not applicable. See "Software" below.

Data quality	Data quality control was performed at the fastq level and at the aligned file level using QuasR's QCReport function. Also the correlation between replicates was assessed.
Software	The log2 fold change of H3K27Ac and statistical testing were computed using the DESeq2 Bioconductor package (Love et al., 2014), providing H3K27Ac counts (calculated using the qCount function of the QuasR package, in 2kb windows covering the genomic regions enriched during the SMF bait capture step) in DMSO or p300i treatment replicates, after applying the normalization factors. The p-values were adjusted using the Benjamini-Hochberg method. Code availability: https://github.com/Krebslabrep/TF-chromatin.git.

Flow Cytometry

Plots

Confirm that:

- [ ] The axis labels state the marker and fluorochrome used (e.g. CD4-FITC).
- [ ] The axis scales are clearly visible. Include numbers along axes only for bottom left plot of group (a 'group' is an analysis of identical markers).
- [ ] All plots are contour plots with outliers or pseudocolor plots.
- [ ] A numerical value for number of cells or percentage (with statistics) is provided.

Methodology

Sample preparation	*Describe the sample preparation, detailing the biological source of the cells and any tissue processing steps used.*
Instrument	*Identify the instrument used for data collection, specifying make and model number.*
Software	*Describe the software used to collect and analyze the flow cytometry data. For custom code that has been deposited into a community repository, provide accession details.*
Cell population abundance	*Describe the abundance of the relevant cell populations within post-sort fractions, providing details on the purity of the samples and how it was determined.*
Gating strategy	*Describe the gating strategy used for all relevant experiments, specifying the preliminary FSC/SSC gates of the starting cell population, indicating where boundaries between "positive" and "negative" staining cell populations are defined.*

- [ ] Tick this box to confirm that a figure exemplifying the gating strategy is provided in the Supplementary Information.

Magnetic resonance imaging

Experimental design

Design type	*Indicate task or resting state; event-related or block design.*
Design specifications	*Specify the number of blocks, trials or experimental units per session and/or subject, and specify the length of each trial or block (if trials are blocked) and interval between trials.*
Behavioral performance measures	*State number and/or type of variables recorded (e.g. correct button press, response time) and what statistics were used to establish that the subjects were performing the task as expected (e.g. mean, range, and/or standard deviation across subjects).*

Acquisition

Imaging type(s)	*Specify: functional, structural, diffusion, perfusion.*
Field strength	*Specify in Tesla*
Sequence & imaging parameters	*Specify the pulse sequence type (gradient echo, spin echo, etc.), imaging type (EPI, spiral, etc.), field of view, matrix size, slice thickness, orientation and TE/TR/flip angle.*
Area of acquisition	*State whether a whole brain scan was used OR define the area of acquisition, describing how the region was determined.*

Diffusion MRI ☐ Used ☐ Not used

Preprocessing

Preprocessing software	*Provide detail on software version and revision number and on specific parameters (model/functions, brain extraction, segmentation, smoothing kernel size, etc.).*

Normalization | *If data were normalized/standardized, describe the approach(es): specify linear or non-linear and define image types used for transformation OR indicate that data were not normalized and explain rationale for lack of normalization.*

Normalization template | *Describe the template used for normalization/transformation, specifying subject space or group standardized space (e.g. original Talairach, MNI305, ICBM152) OR indicate that the data were not normalized.*

Noise and artifact removal | *Describe your procedure(s) for artifact and structured noise removal, specifying motion parameters, tissue signals and physiological signals (heart rate, respiration).*

Volume censoring | *Define your software and/or method and criteria for volume censoring, and state the extent of such censoring.*

Statistical modeling & inference

Model type and settings | *Specify type (mass univariate, multivariate, RSA, predictive, etc.) and describe essential details of the model at the first and second levels (e.g. fixed, random or mixed effects; drift or auto-correlation).*

Effect(s) tested | *Define precise effect in terms of the task or stimulus conditions instead of psychological concepts and indicate whether ANOVA or factorial designs were used.*

Specify type of analysis: ☐ Whole brain ☐ ROI-based ☐ Both

Statistic type for inference | *Specify voxel-wise or cluster-wise and report all relevant parameters for cluster-wise methods.*

(See Eklund et al. 2016)

Correction | *Describe the type of correction and how it is obtained for multiple comparisons (e.g. FWE, FDR, permutation or Monte Carlo).*

Models & analysis

n/a	Involved in the study
☐	☐ Functional and/or effective connectivity
☐	☐ Graph analysis
☐	☐ Multivariate modeling or predictive analysis

Functional and/or effective connectivity | *Report the measures of dependence used and the model details (e.g. Pearson correlation, partial correlation, mutual information).*

Graph analysis | *Report the dependent variable and connectivity measure, specifying weighted graph or binarized graph, subject- or group-level, and the global and/or node summaries used (e.g. clustering coefficient, efficiency, etc.).*

Multivariate modeling and predictive analysis | *Specify independent variables, features extraction and dimension reduction, model, training and evaluation metrics.*
