# Supplementary material for: Cumulative transcription factor binding and p300-mediated histone acetylation drive enhancer activation frequency
Source: Nat Genet. 2026 Jul 31;58(8):1976–87. doi: 10.1038/s41588-026-02703-x (PMC13447113; doi:10.1038/s41588-026-02703-x)
Supplement: Supplementary file 3 [file 41588_2026_2703_MOESM3_ESM.pdf]

nature portfolio

Peer Review File

Cumulative transcription factor binding and p300-mediated histone acetylation drive enhancer activation frequency

Corresponding Author: Dr Arnaud Krebs

Version 0:

Decision Letter:

16th Apr 2025

Hi Arnaud,

hope you're doing well. Apologize for the delay in providing these reports.

Your Article, "Cumulative TF binding and H3K27 Acetylation drive enhancer activation frequency" has now been seen by 2 referees. You will see from their comments copied below that while they find your work of considerable potential interest, they have raised some concerns that must be addressed. In light of these comments, we cannot accept the manuscript for publication, but would be very interested in considering a revised version that addresses these concerns.

Reviewer #1 thinks the paper is well-executed however, they have doubts on the main claims in your study, still providing specific guidance for improvement.

Reviewer #2 thinks this is a high-quality manuscript and particularly appreciates the pooled CRE reporter assay. They suggest to add some analyses and discuss some important points.

We hope you will find the referees' comments useful as you decide how to proceed. If you wish to submit a substantially revised manuscript, please bear in mind that we will be reluctant to approach the referees again in the absence of major revisions.

To guide the scope of the revisions, the editors discuss the referee reports in detail within the team, including with the chief editor, with a view to identifying key priorities that should be addressed in revision and sometimes overruling referee requests that are deemed beyond the scope of the current study. In this case, we please ask you to address Reviewers' points in full. We hope that you will find the prioritised set of referee points to be useful when revising your study. Please do not hesitate to get in touch if you would like to discuss these issues further.

If you choose to revise your manuscript taking into account all reviewer and editor comments, please highlight all changes in the manuscript text file. At this stage we will need you to upload a copy of the manuscript in MS Word .docx or similar editable format.

We are committed to providing a fair and constructive peer-review process. Do not hesitate to contact us if there are specific requests from the reviewers that you believe are technically impossible or unlikely to yield a meaningful outcome.

If revising your manuscript:

*1) Include a “Response to referees” document detailing, point-by-point, how you addressed each referee comment. If no action was taken to address a point, you must provide a compelling argument. This response will be sent back to the referees along with the revised manuscript.

*2) If you have not done so already please begin to revise your manuscript so that it conforms to our Article format instructions, available here.
Refer also to any guidelines provided in this letter.

*3) Include a revised version of any required Reporting Summary: https://www.nature.com/documents/nr-reporting-summary.pdf

It will be available to referees (and, potentially, statisticians) to aid in their evaluation if the manuscript goes back for peer review.
A revised checklist is essential for re-review of the paper.

Please be aware of our guidelines on digital image standards.

EXTENDED DATA FIGURES

When re-submitting your manuscript, please ensure that any supplementary figures and tables that are crucial to the manuscript's conclusions are converted into Extended Data figures and tables to increase visibility of these data. Extended Data figures and tables are online-only (present in the online PDF and full-text HTML versions of the paper), peer-reviewed display items that provide essential background to the article but are not included in the main article due to space constraints. A maximum of ten Extended Data display items (figures and tables) is permitted.

You may use the link below to submit your revised manuscript and related files:

Link Redacted

Note: This URL links to your confidential home page and associated information about manuscripts you may have submitted, or that you are reviewing for us. If you wish to forward this email to co-authors, please delete the link to your homepage.

If you wish to submit a suitably revised manuscript we would hope to receive it within 6 months. If you cannot send it within this time, please let us know. We will be happy to consider your revision so long as nothing similar has been accepted for publication at Nature Genetics or published elsewhere. Should your manuscript be substantially delayed without notifying us in advance and your article is eventually published, the received date would be that of the revised, not the original, version.

Please do not hesitate to contact me if you have any questions or would like to discuss the required revisions further.

Nature Genetics is committed to improving transparency in authorship. As part of our efforts in this direction, we are now requesting that all authors identified as 'corresponding author' on published papers create and link their Open Researcher and Contributor Identifier (ORCID) with their account on the Manuscript Tracking System (MTS), prior to acceptance. ORCID helps the scientific community achieve unambiguous attribution of all scholarly contributions. You can create and link your ORCID from the home page of the MTS by clicking on 'Modify my Springer Nature account'. For more information please visit please visit www.springernature.com/orcid.

Thank you for the opportunity to review your work.

Very best wishes,
Chiara

Chiara Anania, PhD
Associate Editor
Nature Genetics
https://orcid.org/0000-0003-1549-4157

Referee expertise:

Referee #1: gene regulation, TFs

Referee #2: single-molecule, genomics

Referee #3:

Reviewers' Comments:

Reviewer #1 (Remarks to the Author):

In this paper, Baderna et al combine single-molecule footprinting (SMF), perturbations to regulators, and pooled genomic assays to investigate the determinants of enhancer accessibility frequency in mouse embryonic stem cells. They first develop a computational method, FootprintCharter, to quantify the frequency of chromatin accessibility at individual enhancers, and apply it to find that chromatin is accessible in a minority of cells at active enhancers. They then perturb TFs using both F1 hybrid lines and rapid degradation experiments to show that individual TFs have small contributions to accessibility frequency. Finally, they developed a pooled SMF assay measure enhancer accessibility at an ectopic locus and combine it with p300 inhibition to find that the histone mark H3K27ac increases chromatin accessibility frequency.

Overall, I find the paper to be well-executed and utilize innovative techniques. However, I have some doubts about each of the major 3 claims, detailed below:

Claim 1: Active enhancers are accessible in a minority of cells
This claim depends heavily on the definition of an 'enhancer.' Here they analyze a previously generated bait-capture dataset, which looking back seems like it uses a kit to enrich active regulatory regions. It is not clear to me what exactly these regulatory regions are and how they were defined. It would be helpful if the authors could assess this more explicitly – how many of the assessed regions overlap with mESC hypersensitive sites/H3K27ac peaks etc?
The 'unbound' sites presumably mean distal regulatory regions that did not show binding of any of the 18 TFs for which ChIP-seq data were available. But this doesn't preclude them being enhancers/bound by other TFs.
The authors arrive at the main conclusion after separating CTCF-bound loci from the rest of enhancers. I am not sure of the rationale behind this, other than the fact that CTCF does indeed seem to have large effects on accessibility. Still, surely many CTCF-bound loci function as active enhancers, so I would guess that even if a few of those were included it would push the average chromatin accessibility frequency over 50%. While I think there is value in these types of quantitative estimates, it is challenging to boil them down to simple 'minority' or 'majority' when it depends heavily on how enhancers are defined.

Claim 2: TFs contribute 'cumulatively' and individually to a small degree to chromatin accessibility
The experiments that they claim to show this are overall solid, with two major caveats.
- For the F1 hybrid experiment, it is likely that the predicted alternate sites do have lower TF binding, but we do not know by how much. Knowing this is important to interpreting the data – if altered motifs only change the amount of TF binding by 50% instead of completely eliminating, that would suggest stronger individual TF contributions. The authors could perform ChIP seq/qPCR for a handful of TFs in the F1 hybrid lines to verify that the predicted motif affinity changes indeed result in strong (presumably near complete) loss of TF binding. While ChIP is not as quantitative as SMF, it should still allow for a reasonable estimation of the effect on binding.
- For the Sox2 degron experiment, the authors assessed accessibility at 2h post-degradation. While this is useful for looking at direct effects, it is likely that at least some enhancers have not reached a steady state by 2h, and would still continue to decrease in accessibility after that. Indeed Maresca et al 2023 show this explicitly with a 2 to 24h time course. This would result in Sox2 degradation having a larger effect if the authors waited longer. Waiting until 24h alone is complicated by indirect effects, but if the authors did a 24h experiment and used that to assess changes at only the direct target enhancers that change significantly at 2h, that could be one way around it.
With regards to the novelty of the claim, while I think there is value in quantitatively demonstrating cumulative TF contributions as the authors do, this idea has been put forward many times, in many different forms. See reviews by Jindal 2021, Spitz 2012, Long 2016, Kim 2023, etc. All of the major models of enhancer activity (except the pioneer model as authors allude to) incorporate some aspect of cumulative TF function.

Claim 3: H3K27ac deposition facilitates chromatin accessibility frequency
This claim relies on two main pieces of evidence:
- H3K27ac levels correlate with chromatin accessibility frequency only at endogenous and not ectopically inserted CREs.
o While the authors show that the ectopic locus without insertions is H3K27ac negative, an additional important control would be to show that it continues to be H3K27ac negative when CREs are inserted.
- Reducing H3K27ac deposition selectively effects endogenous and not ectopic loci.
o I find the evidence here to be rather weak. It mainly seems to rest on Figure 6c, and the fact that CREs that lose H3K27ac also lose accessibility at their endogenous but not ectopic location. However the distribution of the accessibility changes at the ectopic sites is still clearly below 0, suggesting that p300 inhibition is still having an effect on these ectopic sites. Perhaps it is because these ectopic sites have gained H3K27ac, or maybe there are indirect effects on accessibility through modifications in TF activity, but regardless it is weak support for their claim.
Finally, the authors should discuss their results in light of Doughty et al, Nature 2024, who show that p300 inhibition in a synthetic setting does not affect single-molecule nucleosome occupancy, and that inhibiting BAF is what affects occupancy.

Reviewer #1 (Remarks on code availability):

The approach described in FootprintCharter seems reasonable, a fairly straightforward clustering of smoothed binary methylation tracks. If the authors wish for this to be a useful standalone package for use by others, however, they should provide some more detail on the specific parameter choices used and how robust the outputs tend to be to those parameters. For instance why 40bp smoothing windows, and what is a typically value for n (user-defined minimum # molecules per cluster)?. The code has been made publicly available as part of the SingleMoleculeFootPrinting Bioconductor package. However, the vignette which presumably explains how to run FootprintCharter is empty.

Reviewer #2 (Remarks to the Author):

In their manuscript, "Cumulative TF binding and H3K27 Acetylation drive enhancer activation frequency," Baderna, Barzaghi et al perform single-molecule footprinting (GpC methylase footprinting) of mouse embryonic stem cell (mESC) chromatin, with the goal of absolutely quantifying the accessibility landscape of mouse enhancer sequences, conditioned on the binding of specific sets of transcription factors (TFs). This is a well-written and important submission that uses a variety of molecular and computational analyses to arrive at some important conclusions regarding mouse enhancers: (1) enhancers

are generally less accessible than promoters at the single-molecule level; (2) the cumulative action of multiple TFs instructs enhancer accessibility and activation; (3) H3K27ac specifically increases the frequency of enhancer accessibility, thus (presumably) facilitating TF dynamics and enhancer activation. We generally feel that this is a high-quality manuscript well-suited to the broad readership of Nature Genetics. We have a few comments that we think would strengthen an already excellent manuscript.

Major Comments
• In Figure 2, the authors demonstrate a correlation between chromatin accessibility and the number of TFs bound. This makes intuitive sense, but could be strengthened with more quantitative analysis. Specifically, can the authors examine how chromatin accessibility scales with different motif strengths for a specific TF, for instance CTCF, REST, or NRF1?

• In Figure 3f, the difference in the percentage of chromatin accessibility (%CA) upon Sox2-depletion appears to be clearest in the intermediate range (i.e. 25 – 60%), while motifs where %CA was high (i.e. > 80%) in untagged cells do not seem to be impacted. The authors should clarify what they believe might be occurring here. Is this a consequence of centering on SOX2-OCT4 composite motifs, or perhaps motifs with a specific surrounding sequence context? The authors might address this question by examining the trend when plotting %CA between Unt and Sox2-depletion conditions, scaled by Sox2 motif scores. Perhaps there are strong Sox2 binding sites (higher Sox2 motif scores) which continue to remain bound by Sox2 after 2h of depletion, which would be interesting to know and report.

• The pooled CRE reporter assay is a real source of novelty in this manuscript – this is an elegant experiment and the results are quite informative. However, the authors do not discuss whether there are elements that reduce accessibility “below” background. Are such effects “invisible” to this assay / analysis?

• Recent published work from Gerrard group (PMID38956169), & unpublished preprint from Ramani group (https://doi.org/10.1101/2025.01.17.633622) suggest that there may be a wide range of altered nucleosome structures including subnucleosomes at enhancers. Can the authors comment on the ability of GpC methylase footprinting to resolve these structures, or at least on the possibility of these structures existing at some high SMF binding sites? This seems important when considering the energy balance between TFs and nucleosomes – the energetic constraints required for nucleosome-mediated cooperativity may be different if TFs must outcompete a hexasome, or an altered nucleosomal structure (even in the absence of ATP-dependent chromatin remodeling per se).

Minor Comments
• Line 364 seems to have a typo: Sox2-depletion" vs. "Sox2-deletion?”

• Related to the point above about decreased chromatin accessibility relative to the mean, are there ectopic CRE that have no impact on accessibility? Are these CRE enriched for “off-cell-type” motifs?

• For Supplementary Figure 5b, are there any interesting properties to highlight of the 1,371 sites that actually increase in H3K27ac following p300i?

Version 1:

Decision Letter:

Our ref: NG-A68116R

15th Jan 2026

Dear Dr. Krebs,

Thank you for submitting your revised manuscript entitled "Cumulative TF binding and H3K27 Acetylation drive enhancer activation frequency" (NG-A68116R). It has now been seen by the original referees and their comments are below. The reviewers find that the paper has improved in revision, and therefore we'll be happy in principle to publish it in Nature Genetics, pending minor revisions to satisfy the referee's final requests and to comply with our editorial and formatting guidelines.

The current version of your manuscript is in a PDF format. Please email us (natgen@us.nature.com) a copy of the file in an editable format (Microsoft Word) - we can not proceed with PDFs at this stage.

We will then be performing detailed checks on your paper and will send you a checklist detailing our editorial and formatting requirements soon. Please do not upload the final materials and make any revisions until you receive this additional information from us.

Thank you again for your interest in Nature Genetics. Please do not hesitate to contact me if you have any questions.

Congratulations!

Sincerely,

Tiago

Tiago Faial, PhD
Chief Editor
Nature Genetics
https://orcid.org/0000-0003-0864-1200

Reviewer #1 (Remarks to the Author):

Overall, the authors have addressed the major concerns I raised. My remaining comments are as follows (organized by the claims I originally had concerns about):

Claim 1
I think that the authors should acknowledge that their measurement of “accessibility” is dependent on the amount of time the cells are incubated with the methyltransferase. The field seems to have coalesced around a 7.5 minute incubation period as sufficient for measuring a “snapshot,” which is fine, but it should be stated as such for the readers information.

Claim 2
Supplementary Figure 5a is confusing – y-axis is ref – alt but then there are separate boxplots for “R” and “A”? The p-values are from comparing both the ref and alt boxplots between the “conserved motifs” and the “allele-specific” or “loss of TF binding motifs” categories, but isn’t the point to compare the ref and alt alleles *within* the “allele-specific” or “loss of TF binding motifs” categories? I may be wrong but the figure is overall hard to interpret. Some more clarity in this important control would be appreciated.

Minor note in the discussion: The authors state “The residence time of TFs is typically in the range of a few seconds.” It would be good to have references for this, but also I note that the actual residence time of TFs at their cognate motifs is still very much under debate. While single-particle tracking experiments suggest ~seconds residence time, see Schaepe et al 2025 who demonstrate a > 1 min residence time for KLF4 in cells. I think the discussion would benefit from acknowledging this uncertainty.

Reviewer #2 (Remarks to the Author):

The authors have addressed all of our comments. We commend them on a fantastic piece of work that will be of great interest to Nature Genetics' readership.

Open Access This Peer Review File is licensed under a Creative Commons Attribution 4.0 International License, which

permits use, sharing, adaptation, distribution and reproduction in any medium or format, as long as you give appropriate credit to the original author(s) and the source, provide a link to the Creative Commons license, and indicate if changes were made.
In cases where reviewers are anonymous, credit should be given to 'Anonymous Referee' and the source.
The images or other third party material in this Peer Review File are included in the article's Creative Commons license, unless indicated otherwise in a credit line to the material. If material is not included in the article's Creative Commons license and your intended use is not permitted by statutory regulation or exceeds the permitted use, you will need to obtain permission directly from the copyright holder.
To view a copy of this license, visit https://creativecommons.org/licenses/by/4.0/

Reviewer comments:

Reviewer #1:

In this paper, Baderna et al combine single-molecule footprinting (SMF), perturbations to regulators, and pooled genomic assays to investigate the determinants of enhancer accessibility frequency in mouse embryonic stem cells. They first develop a computational method, FootprintCharter, to quantify the frequency of chromatin accessibility at individual enhancers, and apply it to find that chromatin is accessible in a minority of cells at active enhancers. They then perturb TFs using both F1 hybrid lines and rapid degradation experiments to show that individual TFs have small contributions to accessibility frequency. Finally, they developed a pooled SMF assay measure enhancer accessibility at an ectopic locus and combine it with p300 inhibition to find that the histone mark H3K27ac increases chromatin accessibility frequency. Overall, I find the paper to be well-executed and utilize innovative techniques. However, I have some doubts about each of the major 3 claims, detailed below:

We were glad to read that the reviewer finds our approach relevant and the paper to be technically sound and innovative. We would like to thank them for their constructive and the structured suggestions. We have performed several experiments and analyses to consolidate each of the 3 major claims of the paper.

1) **Refined annotation of enhancers and detailed analysis of well-characterized enhancers** to confirm that they are accessible in a fraction of the cells (**Claim1**) (Supplementary Figure 2).

2) **Allele-resolved ChIP-seq for 3 TFs (Sox2, Klf4 and CTCF)** to directly link the loss of binding on one allele, with the resulting changes in chromatin accessibility. This confirmed that individual TF binding events can only explain part of the chromatin accessibility observed at the enhancers they bind (**Claim2**) (Supplementary Figure 5).

3) **Measuring the chromatin accessibility of a new synthetic DNA library where TF binding sites were mutated individually or in combinations** for 2 individual CREs. This demonstrates the cumulative effect of TFs, using fully controlled variation of the same initial enhancer sequence. (**Claim 2**) (Figure 5).

4) We more than **doubled the number of CREs inserted at the ectopic site (now >800) and profiled their chromatin accessibility upon p300 inhibition (>200 retained for the analysis)**. This confirmed our initial observations that H3K27Ac contributes to chromatin accessibility at endogenous enhancers, but not at the ectopic sites. Adding more elements significantly strengthen the statistics, confirming the cumulative function between TF and histone acetylation in opening chromatin (**Claim3**) (Figure 6).

We hope that these additional experiments and analysis will convince the reviewer of the solidity of our main claims. Below is a point-by-point response to their comments:

Claim 1: Active enhancers are accessible in a minority of cells

This claim depends heavily on the definition of an 'enhancer.' Here they analyse a previously generated bait-capture dataset, which looking back seems like it uses a kit to enrich active regulatory regions. It is not clear to me **what exactly these regulatory regions** are and **how they were defined**. It would be helpful if the authors could assess this more explicitly.

We agree with the reviewer that the initial version of the manuscript did not provide with sufficient details on which part of the genome was captured by the baits and how we defined enhancers. In a nutshell, we used a commercial design of Agilent that was based on DNAse Hypersensitivity that captures >60% of the active regions in mESCs (enhancers/promoters) and used chromHMM to annotate enhancers and promoters based on chromatin marks. We confirmed that the annotated elements harbor canonical chromatin marks at enhancers (H3K4me1 and H3K27Ac) and promoters (H3K4me3 and H3K27Ac) (Supplementary Figure 2). We have now included explicit statements in the main text to describe how the capture was designed, a paragraph in the method section that detail how we annotated enhancers and a heatmap of the chromatin marks present at the studied loci.

Statement in the main text:

"**We used a previously generated bait-capture SMF dataset that profiles >60% of the DNAse-hypersensitive sites active in mESCs at a sufficient coverage for single molecule quantification** (Sönmezer et al., 2021) (Supplementary Figure 1b-c). We mapped all motif instances in the genome (Mathelier et al., 2016) and identified bound motifs using publicly available ChIP-seq and ChIP-nexus datasets as a reference for the binding of 18 TFs expressed in mESCs (Supplementary Table 1, see Methods). **We used an existing genome segmentation based on chromatin marks (chromHMM)** (Pintacuda et al., 2017) **to annotate TFBSs with the type of CRE they belong to (enhancer, promoter, CTCF) (see methods, Supplementary Figure 2a). While CTCF binds on its own at most loci, we noted <6% of its active motifs are at CREs categorised as enhancers. The depth of our data allowed to sample a large collection of CREs harbouring canonical combinations of chromatin marks found at enhancers (H3K4me1, H3K27Ac) and promoters (H3K4me3, H3K27Ac) (Supplementary Figure 2a, b).**"

Statement in the methods section:

"Annotation of enhancers and promoters.

We classified bound TFBS as a function of the type of CRE they are bound to. We used an existing annotation based on 12-states chromHMM to classify CREs as promoters or enhancers (Pintacuda et al., 2017). For simplicity, we selected and pooled states as CTCF (1_Insulator), intergenic (2_Intergenic), enhancer (4_Enhancer, 8_StrongEnhancer, 11_WeakEnhancer), promoter (7_ActivePromoter). Of note, this classification does not simply discriminate CREs based on TF identity, therefore the enhancer category includes 2,200 CTCF-bound loci. We then analysed chromatin accessibility at all enhancers and promoters covered in the bait-capture data Agilent (#931052). These baits cover 59.7% of open regions as detected by DNase sequencing, covering all annotated promoters and a majority of the enhancers active in mESCs (Kreibich et al., 2023; Sönmezer et al., 2021).

"

Moreover, to confirm that our conclusion on enhancer accessibility is not a function of our annotation, we measured the frequency of chromatin accessibility for the well characterized enhancers of the Sox2 Locus Control Region (Supplementary Figure 2c). We found that the median accessibility to be below 50% and observed significant levels of nucleosome occupancy at example loci (Supplementary Figure 2b). We described this additional evidence in the main text: 'Intermediate levels of chromatin accessibility could also be observed at well-characterized enhancers such as enhancers from the Sox2 locus-control region (Supplementary Figure 2b, c), confirming that partial chromatin accessibility is a general feature of enhancers.'

We hope that these clarifications and additional analyses will clarify this issue and convince the reviewer of the solidity of our claim on the heterogeneity of chromatin accessibility at enhancers.

– how many of the assessed regions overlap with mESC hypersensitive sites/H3K27ac peaks etc? The 'unbound' sites presumably mean distal regulatory regions that did not show binding of any of the 18 TFs for which ChIP-seq data were available. But this doesn't preclude them being enhancers/bound by other TFs.

We agree that we have not been entirely explicit on the definition of states, including the unbound state. To further characterize the loci included in our analysis, we plotted the distribution of chromatin marks around them (Supplementary Figure 2a). This shows that loci classified as enhancers and promoters are accessible by DHS and harbor histone modifications characteristic of promoters and enhancers; including high levels of H3K27Ac at most of them. This further shows that 'unbound' TFBS are mostly inaccessible and do not have chromatin marks of enhancers and promoters. Thus, while we agree that we cannot exclude the binding of other TFs not measured in this study, we believe that this strengthen our assumption that these sites are not active regulatory elements. We hope that this additional description of our classification and its rational will convinced the reviewer of the solidity of our conclusion on the heterogeneity of chromatin accessibility at enhancers.

The authors arrive at the main conclusion after separating CTCF-bound loci from the rest of enhancers. I am not sure of the rationale behind this, other than the fact that CTCF does indeed seem to have large effects on accessibility. Still, surely many CTCF-bound loci function as active enhancers, so I would guess that even if a few of those were included it would push the average chromatin accessibility frequency over 50%. While I think there is value in these types of quantitative estimates, it is challenging to boil them down to simple 'minority' or 'majority' when it depends heavily on how enhancers are defined.

We thank Reviewer #1 for the opportunity to clarify our treatment of CTCF-bound loci across our study. The enhancer and CTCF-bound loci were derived from an existing 12-states chromHMM (Pintacuda et al., 2017). In turn the "enhancers" category reported in Figure 1d is comprised of >37,000 distinct loci, that include 2,200 CTCF-bound enhancers. On the other hand, the "CTCF" category is comprised of CTCF-bound loci which are not otherwise enriched chromatin marks typical of other regulatory elements (H3K4me1/3 or H3K27Ac). We have clarified both aspects in the main text and added a heatmap that displays the chromatin states of each category (Supplementary Figure 2a).

We understand that the reviewer may have rather referred to Figure 3b, where we excluded CTCF-bound loci from the analysis of loss of accessibility across alleles. Our rational was to highlight the high level of heterogeneity in chromatin accessibility at enhancers compared to other regulatory elements (promoters/CTCF). However, motivated by this comment, we controlled whether our estimates would change in the presence of CTCF motifs. The new panel Supplementary Figure 4n shows that the inclusion of CTCF motifs in the analysis does not noticeably impact our estimate of chromatin accessibility contributed by single TF binding events. To clarify our treatment of CTCF-bound loci, we added the following statement to the main text.

Statement in the main text:
"We observed that most motif disruptions led to a moderate reduction in accessible molecules (<10% median, Supplementary Figure 4m), while complete loss of chromatin accessibility at CREs was rare (Figure 3b). **Including the CTCF-bound enhancers in this analysis did not change the general trend observed and the conclusion of this analysis (Supplementary Figure 4n).**

Claim 2: TFs contribute 'cumulatively' and individually to a small degree to chromatin accessibility
The experiments that they claim to show this are overall solid, with two major caveats.

We were glad to read that the reviewer felt the experiments supporting the cumulative hypothesis were solid. We have addressed both of his technical concerns below. Additionally, we added an experiment that test this hypothesis with an orthogonal approach. We synthetized 42 sequence variants that delete individual or combination of TF motifs at a promoter and an enhancer. We subsequently tested the effect of each mutation on chromatin accessibility using our reporter system (mCHIRA). The data presented in a new version of Figure 5 unambiguously confirm the partial contribution of individual TFs (Klf4, NRF1, Myc and Yy1), as well as their cumulative action to reach high levels of chromatin accessibility. This highly controlled experiment complements our genome-wide observations bringing a new line of evidence that considerably strengthen this claim.

- For the **F1 hybrid** experiment, it is likely that the predicted alternate sites do have lower TF binding, but we do not know by how much. Knowing this is important to interpreting the data – if altered motifs only change the amount of TF binding by 50% instead of completely eliminating, that would suggest stronger individual TF contributions. The authors could perform **ChIP seq/qPCR for a handful of TFs** in the F1 hybrid lines to verify that the predicted motif affinity changes indeed result in strong (presumably near complete) loss of TF binding. While ChIP is not as quantitative as SMF, it should still allow for a reasonable estimation of the effect on binding.

We thank the reviewer for suggesting this additional control to formally demonstrate that TF binding is lost at loci where a SNP alters the TF binding site. As suggested, we performed allele-resolved ChIP-seq for three TFs (Klf4, CTCF and Sox2), providing with an estimate of genome-wide differences in TF occupancy between alleles. These data confirmed the differences in TF occupancies between alleles at motifs instances that we classified to be of low affinity on the alternative allele. Moreover, we analyzed the changes in chromatin accessibility for motifs where we estimated that TF binding was completely lost by ChIP-seq (Supplementary figure 5a). Similar to our initial analysis, we observed only a partial loss in chromatin accessibility at these loci (Supplementary figure 5b). This control strengthens our analysis and confirms that most TFs have only partial contribution to the chromatin accessibility observed at CREs. We added the following statements to the main text.

Statement in the main text:
"To confirm the loss of TF occupancy at SNP-containing motifs, we performed ChIP-seq for CTCF, KLF4 and SOX2 in the Bl6xSpret F1 line. We observed a consistent loss of TF binding at motifs predicted to be significantly affected by SNPs on the alternative allele (Supplementary

Fig. 5a). When restricting our analysis on CREs showing complete loss of TF binding by ChIP-seq, we also observed partial loss of chromatin accessibility (Supplementary Fig. 5a,b)."

- For the **Sox2 degron** experiment, the authors assessed accessibility at 2h post-degradation. While this is useful for looking at direct effects, **it is likely that at least some enhancers have not reached a steady state by 2h**, and would still continue to decrease in accessibility after that. Indeed Maresca et al 2023 show this explicitly with a 2 to 24h time course. This would result in Sox2 degradation having a larger effect if the authors waited longer. Waiting until 24h alone is complicated by indirect effects, but if the authors did a 24h experiment and used that to assess changes at only the direct target enhancers that change significantly at 2h, that could be one way around it.

We followed the precise logic described by the reviewer when designing this experiment. We picked an early time point where the Sox2 is fully degraded (protein is mostly gone at 30 min already), but where secondary effects are still limited. Sox2 is essential for mESCs that start to differentiate rapidly upon degradation. 24h is longer than the average mESC cell cycle (~15h), thus many cells start to either differentiate or die. We had also re-analyzed the ATAC-seq data from Maresca et al, 2023, and found that most of the changes observed at 24h were already present at 2h. This guided our choice of time point for the experiment. We are now displaying the analysis of this time course data in Supplementary Figure 5g-i and hope the reviewer agrees on our rational for experimental design. We added the following statement to the main text:

"To confirm these observations with an orthogonal approach, we quantified the changes in chromatin accessibility frequency at SOX2-bound enhancers after 2h treatment using a degron system (Figure 3e, Supplementary Figure 5c, d). At this early time point, >90% of SOX2 is degraded (Supplementary Figure 5c), and re-analysis of existing ATAC-seq time course data shows that most of the changes in chromatin accessibility observed at SOX2 binding sites at 24h are already present (Supplementary Figure 5g-i)."

With regards to the novelty of the claim, while I think there is value in quantitatively demonstrating cumulative TF contributions as the authors do, this idea has been put forward many times, in many different forms. See reviews by Jindal 2021, Spitz 2012, Long 2016, Kim 2023, etc. All of the major models of enhancer activity (except the pioneer model as authors allude to) incorporate some aspect of cumulative TF function.

We agree that the idea that TFs cumulatively act at CREs is not new and that the model has been proposed in serval reviews, including those listed by the reviewer. There is nevertheless a fair amount of debate in the field since the concept of 'pioneer' TFs has been put forward on whether this function may be hold by a specific set of TFs.

Our work represents the first demonstration at scale that TFs act cumulatively to open chromatin at CREs, estimating the contribution of a broad range of TFs. Unambiguously demonstrating the cumulative hypothesis is very challenging when using bulk assays as they provide with relative measure that are difficult to compare across experiments. This was a big limitation so far. Moving in single molecule space allows now to directly compare effects within very different experiments that perturb TF function (F1, degrons, mCHIRA) and directly

measure how much each TF binding at a CRE contribute to chromatin accessibility. Our data indicate that even TFs that were described like secondary binders such as Myc, contribute to the chromatin accessibility at their binding sites (Figure 5). We thus believe that these conclusions are shifting our thinking of how TFs assemble in collectives and will have a strong impact on the field. We nevertheless agree with the reviewer that our introduction was insufficiently referencing reviews that discuss the cumulative concept. We now revised the respective paragraph and included the suggested references we could identify.

"Several mechanistic models have been proposed to explain how TFs combine their function to initiate and maintain chromatin accessibility. One model postulates that a specific class of TFs is specialized in opening chromatin (named 'pioneer'), while others depend on their prior binding to exert other functions such as transcription stimulation (Barral and Zaret, 2024; Reiter et al., 2017). In support of this concept, sequential binding, and functional dependency between TFs have been observed in the activation of lineage-specifying CREs in multiple developmental contexts (Barral and Zaret, 2024; Bulyk et al., 2023; Reiter et al., 2017). Additionally, only a fraction of the TFs are able to bind their motifs when DNA is wrapped around nucleosomes, while the majority of TFs are only able to engage with free DNA (Dodonova et al., 2020; Grand et al., 2024; Michael et al., 2023; Zhu et al., 2018). **An alternative, complementary model proposes that most TFs exert only low levels of chromatin opening activity when binding individually, and that the cumulative function of multiple TFs is required to open chromatin at sufficient levels to activate transcription** (Jindal and Farley, 2021; Kim and Wysocka, 2023; Long et al., 2016; Reiter et al., 2017; Spitz and Furlong, 2012)."

Claim 3: H3K27ac deposition facilitates chromatin accessibility frequency
This claim relies on two main pieces of evidence:

- H3K27ac levels correlate with chromatin accessibility frequency only at endogenous and not ectopically inserted CREs.
o While the authors show that the ectopic locus without insertions is H3K27ac negative, an additional important control would be to show that it continues to be H3K27ac negative when CREs are inserted.

Our assumptions in the primary version of the paper build on previous work suggesting that individual CREs tend to reflect aspects of the chromatin environment at their site of insertion (Hong and Cohen, 2022; Chen et al., 2013).

Measuring H3K27Ac in libraries inserted at the ectopic locus is challenging as the relative abundance of each fragment varies, and is thousand times less than endogenous genomic sequences. We previously tried to do ChIP followed by PCR to selectively amplify the ectopic site and sequencing. Unfortunately, the amplification of the inserts after ChIP-seq creates huge skew in the distribution of the fragments in both IP and input. This renders the relative quantification of abundance of fragments in IP and input very noisy, poorly sensitive and non-quantitative. We now tried to use H3K27Ac ChIP qPCR for a couple of CREs for which we observed significant differences of behaviour of ectopic and endogenous upon p300i. While we could detect reliable enrichment at the endogenous locus after 22-25 cycles of amplification, the signal was below the detection limit of qPCR for both fragments we tested at the ectopic site with Ct values >32Ct. Despite our efforts to increase the amount of material

used in the qPCR, we could not reliably quantify the H3K27Ac levels at the ectopic locus (see below figure). While the results suggest that the H3K27Ac are low, the technical variability is high and we decided not to include this panel in the manuscript. We however added a statement describing our rational and the relevant reference in the new version of the manuscript.

"This idea is supported by the fact that short CRE fragments were shown to adopt the chromatin state of the locus where they are inserted (Hong and Cohen, 2022; Chen et al., 2013). "

Additionally, to compensate for this limitation, we inserted a new library containing hundreds of new fragments, bringing the total number of H3K27Ac sensitive fragments from 13 to 98 individual measurements (see below). This increases the numbers of fragments where we see a disconnect between global loss of H3K27Ac and change in chromatin accessibility when inserted at the ectopic locus. It strongly suggests that this loss may be related to a reduction in the H3K27Ac, as this is the main feature that is consistently shared by these CREs.

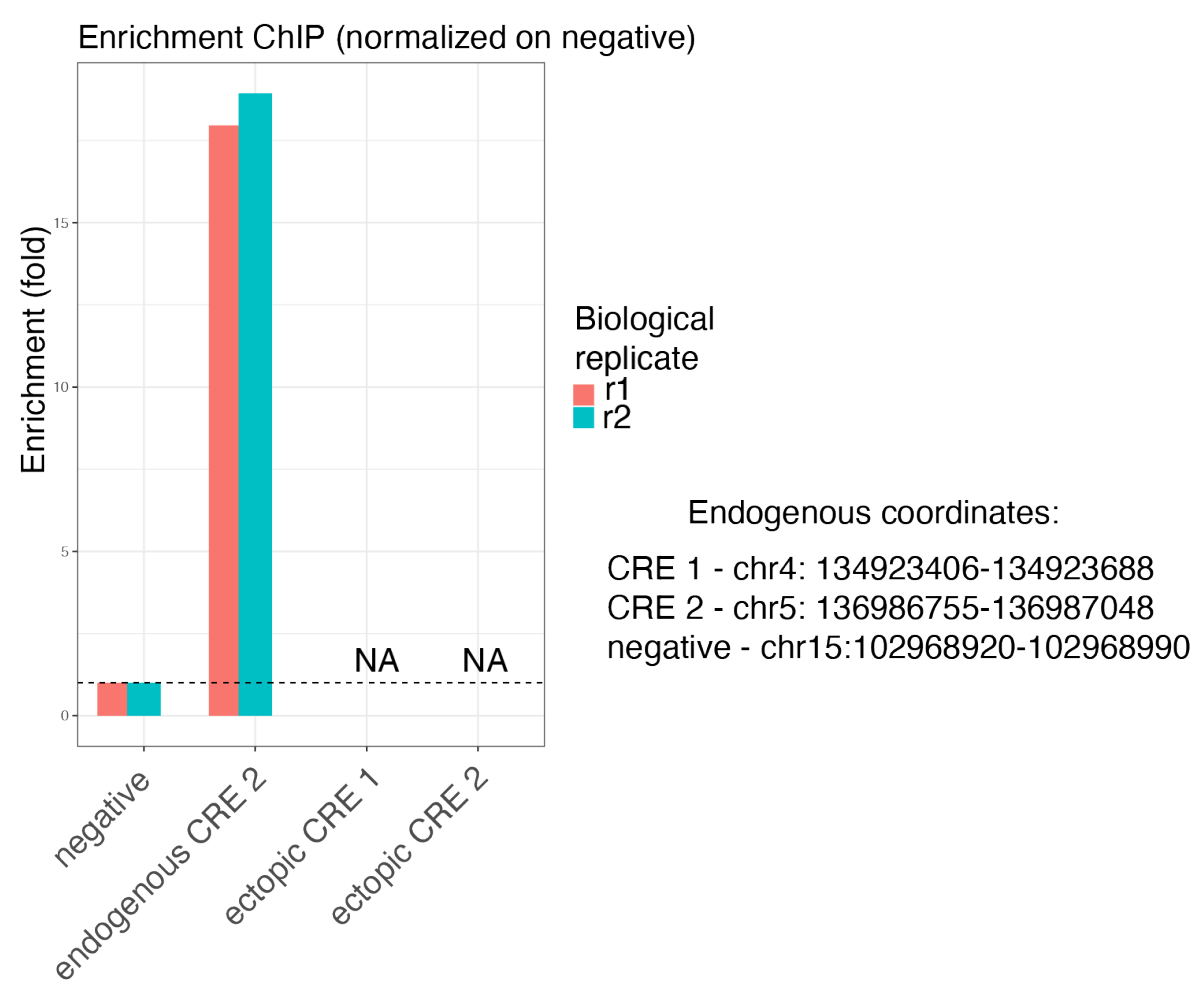

- Reducing H3K27ac deposition selectively effects endogenous and not ectopic loci.

o I find the evidence here to be rather weak. It mainly seems to rest on Figure 6c, and the fact that CREs that lose H3K27ac also lose accessibility at their endogenous but not ectopic location. However the distribution of the accessibility changes at the ectopic sites is still clearly below 0, suggesting that p300 inhibition is still having an effect on these ectopic sites. Perhaps it is because these ectopic sites have gained H3K27ac, or maybe there are indirect effects on accessibility through modifications in TF activity, but regardless it is weak support for their claim.

Our initial statement relied on the analysis of the behavior of a few dozen fragments of our mCHIRA library for which changes in H3K27Ac were observed at the endogenous locus upon p300i (Figure 6c of the initial submissions). While the trends were clear, we agree that the limited number of insertions limited the power of this analysis and the strength of the conclusion we made. In order to strengthen this point, we designed and inserted a new library of 200 CREs sampled to populate each of the categories made in this analysis, increasing the

number of fragments analyzed by a factor of 10. The difference of behavior between ectopic and endogenous fragments upon p300i is now striking (Figure 6f). We observed an increase in effect size and the number of fragments tested now significantly improved the significance of our statistical test. For convenience, we display below the initial and updated version of the figure side by side. We hope the reviewer agrees that this additional data considerably strengthen our claim that H3K27Ac additively contributes to chromatin accessibility.

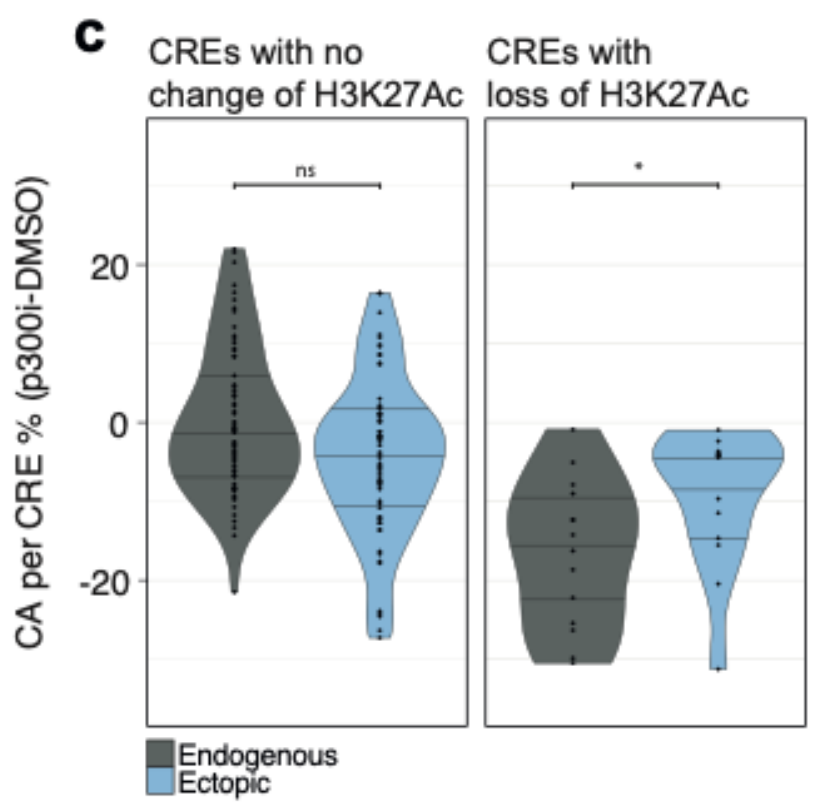

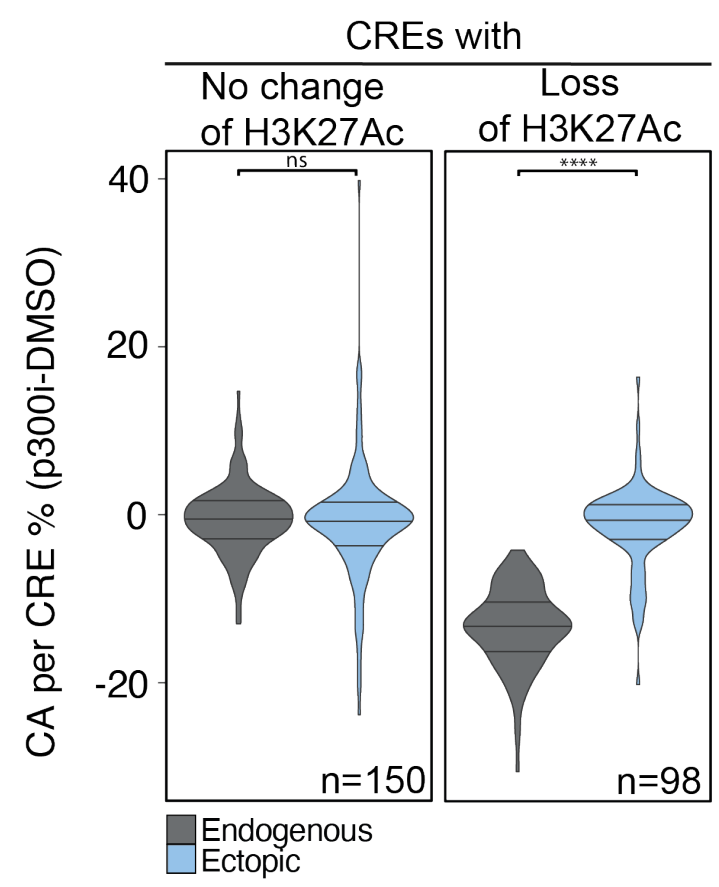

Finally, the authors should discuss their results in light of Doughty et al, Nature 2024, who show that p300 inhibition in a synthetic setting does not affect single-molecule nucleosome occupancy, and that inhibiting BAF is what affects occupancy.

We thank the reviewer for their perspective and the relevant suggestion. We have now included this in our discussion:

'Our data argue that TF binding can occur in absence of H3K27Ac, that can enhance their frequency of occupancy. This is consistent with recent findings that showed that the activity of p300, (but not that of the ATP-dependent chromatin remodeler BAF), is dispensable for synthetic TFs to open chromatin and activate transcription (Doughty et al., 2024).'

Remarks on code availability

The approach described in FootprintCharter seems reasonable, a fairly straightforward clustering of smoothed binary methylation tracks. If the authors wish for this to be a useful standalone package for use by others, however, they should provide some more detail on the specific parameter choices used and how robust the outputs tend to be to those parameters. For instance, why 40bp smoothing windows, and what is a typically value for n (user-defined minimum # molecules per cluster)?. The code has been made publicly available as part of the SingleMoleculeFootPrinting Bioconductor package. However, the vignette which presumably explains how to run FootprintCharter is empty.

We would like to thank Reviewer #1 for the remark and suggestions to improve the distribution of our computational tools. We incorporated FootprintCharter in the updated release of our R/Bioconductor SingleMoleculeFootprinting package available at https://bioconductor.org/packages/SingleMoleculeFootprinting. We apologies for the lack of information in the vignette that was due to a mistake in the update of the repository. An extensive vignette is now available at:

https://bioconductor.org/packages/release/bioc/vignettes/SingleMoleculeFootprinting/inst/doc/FootprintCharter.html.
Moreover, the pre-print has now been revised and published in Bioinformatics, adding further details on FootprintCharter's performance and provide guide on the choice of parameter for users: https://doi.org/10.1093/bioinformatics/btaf502

We hope this will promote the adoption of the tool in the community.

Reviewer #2:

In their manuscript, "Cumulative TF binding and H3K27 Acetylation drive enhancer activation frequency," Baderna, Barzaghi et al perform single-molecule footprinting (GpC methylase footprinting) of mouse embryonic stem cell (mESC) chromatin, with the goal of absolutely quantifying the accessibility landscape of mouse enhancer sequences, conditioned on the binding of specific sets of transcription factors (TFs). This is a well-written and important submission that uses a variety of molecular and computational analyses to arrive at some important conclusions regarding mouse enhancers: (1) enhancers are generally less accessible than promoters at the single-molecule level; (2) the cumulative action of multiple TFs instructs enhancer accessibility and activation; (3) H3K27ac specifically increases the frequency of enhancer accessibility, thus (presumably) facilitating TF dynamics and enhancer activation. We generally feel that this is a high-quality manuscript well-suited to the broad readership of Nature Genetics. We have a few comments that we think would strengthen an already excellent manuscript.

We thank the reviewer for their supportive statement and constructive comments. Although not directly requested, we would like to highlight additional analyses and experiments that further strengthened our conclusions:

- **Allele-resolved ChIP-seq for Sox2, Klf4, and CTCF** to directly link allele-specific loss of TF binding with corresponding changes in chromatin accessibility, confirming that single TF binding events account for only part of enhancer accessibility (Supplementary Figure 5a, b).
- **Chromatin accessibility measurements of a new synthetic DNA library** in which TF binding sites were mutated individually or in combination in two enhancers, demonstrating the cumulative contribution of TFs within a controlled sequence background (Figure 5).
- **Expansion of our ectopic CRE panel to over 800 elements** and profiling their accessibility after p300 inhibition (>200 retained for the analysis), reinforcing that H3K27ac supports accessibility at endogenous but not ectopic enhancers. The larger dataset provides stronger statistical support for the combined roles of TFs and histone acetylation in chromatin opening. (Figure 6)

Major comments

- In Figure 2, the authors demonstrate a correlation between chromatin accessibility and the number of TFs bound. This makes intuitive sense, but could be strengthened with more quantitative analysis. Specifically, can the authors examine how chromatin

accessibility scales with different motif strengths for a specific TF, for instance CTCF, REST, or NRF1?

We thank the reviewer for suggesting this additional analysis. To address this point, we ranked the TF motifs by strength and plotted the median frequency of chromatin accessibility (Supplementary Figure 3b). We observed that the frequency of chromatin accessibility scales as a function of motif strength for a small subset of TFs that include CTCF and REST and YY1. However, we did not observe such a straightforward relation for other TFs. This may be explained by the fact that for most TFs the frequency of accessibility at a locus may mainly relies on how many TFs bind rather than the strength of the binding site. Yet, we would not rule out that motif strength may have an effect that we cannot detect in this analysis.

The corresponding statement can be found in the main text:

'We asked if the strength of the binding motifs of individual TFs influence the frequency of chromatin accessibility (Supplementary Figure 3b). We observed a scaling between motif strength and chromatin accessibility for the factors creating high frequency of chromatin accessibility such as CTCF or REST. Yet, this relation did not hold for most other TFs, suggesting that binding strength of individual TFs is insufficient to explain the frequency of chromatin accessibility of their target CREs.'

- In Figure 3f, the difference in the percentage of chromatin accessibility (%CA) upon Sox2-depletion appears to be **clearest in the intermediate range (i.e. 25 – 60%), while motifs where %CA was high (i.e. > 80%) in untagged cells do not seem to be impacted**. The authors should clarify what they believe might be occurring here. Is this a consequence of centering on SOX2-OCT4 composite motifs, or perhaps motifs with a specific surrounding sequence context? The authors might address this question by examining the trend when plotting %CA between Unt and Sox2-depletion conditions, scaled by Sox2 motif scores. Perhaps there are strong Sox2 binding sites (higher Sox2 **motif scores**) which continue to remain bound by Sox2 after 2h of depletion, which would be interesting to know and report.

We read that comment in relation to the previous one linking the frequency of chromatin accessibility with motif strength. We agree on the observation that Sox2 depletion does not affect accessibility for highly accessible region (Figure 3f). Yet, in our answer of the previous comment, we showed that the frequency of chromatin accessibility at Sox2 binding sites is not scaling with the strength of the motif (Supplementary Figure 3b). Thus, high accessibility sites are not enriched for strong Sox2 binding motifs, and unlikely to explain the lack of response.
Instead, we speculate that may be due to the specific cis-environment of these TF binding sites. High accessibility sites (>70%) tend to be promoters (Figure 1d), where we found that accessibility is the result of the cumulative binding of many TFs, and also the action of epigenetics marks (such as H3K27Ac) (Figure 6). At these loci, the cumulation of these many effects are bringing accessibility to saturation and depletion of individual TFs such as Sox2 can be compensated by other effects -leading to no observable effects.

We made an explicit statement in the manuscript to clarify this issue: 'These effects are most visible for loci with intermediate chromatin accessibility, while highly accessible regions are less affected (Figure 3f). This may be explained by the existence of redundant mechanisms mediating chromatin accessibility at these highly accessible loci."

We also directly tested if the strength of the Sox2 motif could explain the difference in response by plotting the changes in chromatin accessibility for different motif strength categories (Supplementary Figure 5j). We did not see significant differences driven by the TF motif strength. The corresponding statement can be found in the main text:

”We asked whether differences in change amplitude were associated with SOX2 motif strength. We observed similar amplitudes across motifs, indicating that within this range, motifs of all strengths contribute similarly to chromatin accessibility (Supplementary Figure 5j).’

• The pooled CRE reporter assay is a real source of novelty in this manuscript – this is an elegant experiment and the results are quite informative. However, the authors do not discuss whether there are elements that reduce accessibility “below” background. Are such effects “invisible” to this assay / analysis?

We thank the reviewer for this interesting question. The baseline accessibility at regions that are inaccessible and without nucleosome is around 15% (Figure 1d). It is however the result of the movement of nucleosome arrays when nucleosomes are unphased. If there is phasing of the nucleosomes (i.e., upon CTCF binding), the accessibility will move close to 0% at the nucleosome position. This is detected by the assay and can be quantified (i.e. figure 4b, at the phased nucleosome position). Yet, we focused on TF binding sites that are typically not at the phased nucleosome position. One could imagine using these principles to detect repressive factors that act through increasing chromatin compaction. We did not detect such an effect in the current datasets that were biased towards active regions. Yet that would be an interesting future development to apply this assay to study mechanisms of repression.

- Recent published work from Gerrard group (PMID38956169), & unpublished preprint from Ramani group (https://doi.org/10.1101/2025.01.17.633622) suggest that there may be a wide range of altered nucleosome structures including subnucleosomes at enhancers. Can the authors comment on the ability of GpC methylase footprinting to resolve these structures, or at least on the possibility of these structures existing at some high SMF binding sites? This seems important when considering the energy balance between TFs and nucleosomes – the energetic constraints required for nucleosome-mediated cooperativity may be different if TFs must outcompete a hexasome, or an altered nucleosomal structure (even in the absence of ATP-dependent chromatin remodeling per se).

We agree with the reviewer that sub-nucleosomal particles at regulatory elements is a very interesting biological question, and the Ramani paper is a beautiful example of how single molecule methods can address this question. We did observe examples of particles that were smaller than canonical nucleosomes in our data (i.e. Figure 2b, f). Yet the read length of our data (<300bp) is limiting to confidently estimate the size of large particles at scale. We therefore decided not to investigate these more systematically in this study. We included an explicit statement in the revised version of the discussion:

“In this sequential model, the involvement of a broader set of TFs to maintain chromatin open could be facilitated by the formation of destabilised nucleosomes (Brahma and Henikoff, 2020; Yang et al., 2025). **These sub-nucleosomal particles are in principle detected by our assay. Yet, the read length used in this study makes it challenging to unambiguously**

distinguish these from canonical nucleosomes, as has been achieved with methods using longer reads (Yang et al., 2025)**.** "

Minor Comments

- Line 364 seems to have a typo: Sox2-depletion" vs. "Sox2-deletion?"

We would like to thank the reviewer for spotting the typo. We have corrected it.

- Related to the point above about decreased chromatin accessibility relative to the mean, are there ectopic CRE that have no impact on accessibility? Are these CRE enriched for "off-cell-type" motifs?

Almost all ectopic CREs that show the same chromatin accessibility at the ectopic site as the endogenous ones (i.e. 140/145) are bound by mESC TFs in their native genomic context, including KLF4, MYC, OCT4, SOX2, NANOG, ESRRB, indicating that most tested elements carry typical pluripotency TF binding sites. To test whether these are enriched for off–cell-type motifs, we ran AME analysis (https://meme-suite.org/meme/tools/ame) using these CREs as query and CREs that show less accessibility as background. In this comparison, the only motif with enrichment was RORA; its cognate transcription factor RORα is not expressed in mESC and the motif is present ~10% of the 145 sequences. Apart from this weak signal, motif composition is very similar across CRE with higher, similar, or lower accessibility, and we do not detect a clear off-cell-type motif signature in any group.

- For Supplementary Figure 5b, are there any interesting properties to highlight of the 1,371 sites that actually increase in H3K27ac following p300i?

We could not detect any specific properties. These are likely to be mostly secondary effects of the large gene de-regulation and potential alteration of cell cycle. These have been investigated in depth upon changes in acetylation after 24h (Cusack et al., 2020).

Barral, A., Zaret, K.S., 2024. Pioneer factors: roles and their regulation in development. Trends in Genetics 40, 134–148. https://doi.org/10.1016/j.tig.2023.10.007

Brahma, S., Henikoff, S., 2020. Epigenome Regulation by Dynamic Nucleosome Unwrapping. Trends Biochem Sci 45, 13–26. https://doi.org/10.1016/j.tibs.2019.09.003

Bulyk, M.L., Drouin, J., Harrison, M.M., Taipale, J., Zaret, K.S., 2023. Pioneer factors — key regulators of chromatin and gene expression. Nat Rev Genet 1–7. https://doi.org/10.1038/s41576-023-00648-z

Chen, M., Licon, K., Otsuka, R., Pillus, L., Ideker, T., 2013. Decoupling Epigenetic and Genetic Effects through Systematic Analysis of Gene Position. Cell Reports 3, 128–137. https://doi.org/10.1016/j.celrep.2012.12.003

Dodonova, S.O., Zhu, F., Dienemann, C., Taipale, J., Cramer, P., 2020. Nucleosome-bound SOX2 and SOX11 structures elucidate pioneer factor function. Nature 580, 669–672. https://doi.org/10.1038/s41586-020-2195-y

Doughty, B.R., Hinks, M.M., Schaepe, J.M., Marinov, G.K., Thurm, A.R., Rios-Martinez, C., Parks, B.E., Tan, Y., Marklund, E., Dubocanin, D., Bintu, L., Greenleaf, W.J., 2024.

Single-molecule states link transcription factor binding to gene expression. Nature 636, 745–754. https://doi.org/10.1038/s41586-024-08219-w
Grand, R.S., Pregnolato, M., Baumgartner, L., Hoerner, L., Burger, L., Schübeler, D., 2024. Genome access is transcription factor-specific and defined by nucleosome position. Molecular Cell 0. https://doi.org/10.1016/j.molcel.2024.08.009
Hong, C.K.Y., Cohen, B.A., 2022. Genomic environments scale the activities of diverse core promoters. Genome Res. 32, 85–96. https://doi.org/10.1101/gr.276025.121
Jindal, G.A., Farley, E.K., 2021. Enhancer grammar in development, evolution, and disease: dependencies and interplay. Developmental Cell 56, 575–587. https://doi.org/10.1016/j.devcel.2021.02.016
Kim, S., Wysocka, J., 2023. Deciphering the multi-scale, quantitative cis-regulatory code. Molecular Cell. https://doi.org/10.1016/j.molcel.2022.12.032
Kreibich, E., Kleinendorst, R., Barzaghi, G., Kaspar, S., Krebs, A.R., 2023. Single-molecule footprinting identifies context-dependent regulation of enhancers by DNA methylation. Molecular Cell 0. https://doi.org/10.1016/j.molcel.2023.01.017
Long, H.K., Prescott, S.L., Wysocka, J., 2016. Ever-Changing Landscapes: Transcriptional Enhancers in Development and Evolution. Cell 167, 1170–1187. https://doi.org/10.1016/j.cell.2016.09.018
Mathelier, A., Fornes, O., Arenillas, D.J., Chen, C., Denay, G., Lee, J., Shi, W., Shyr, C., Tan, G., Worsley-Hunt, R., Zhang, A.W., Parcy, F., Lenhard, B., Sandelin, A., Wasserman, W.W., 2016. JASPAR 2016: a major expansion and update of the open-access database of transcription factor binding profiles. Nucleic Acids Res 44, D110–D115. https://doi.org/10.1093/nar/gkv1176
Michael, A.K., Stoos, L., Crosby, P., Eggers, N., Nie, X.Y., Makasheva, K., Minnich, M., Healy, K.L., Weiss, J., Kempf, G., Cavadini, S., Kater, L., Seebacher, J., Vecchia, L., Chakraborty, D., Isbel, L., Grand, R.S., Andersch, F., Fribourgh, J.L., Schübeler, D., Zuber, J., Liu, A.C., Becker, P.B., Fierz, B., Partch, C.L., Menet, J.S., Thomä, N.H., 2023. Cooperation between bHLH transcription factors and histones for DNA access. Nature 619, 385–393. https://doi.org/10.1038/s41586-023-06282-3
Pintacuda, G., Wei, G., Roustan, C., Kirmizitas, B.A., Solcan, N., Cerase, A., Castello, A., Mohammed, S., Moindrot, B., Nesterova, T.B., Brockdorff, N., 2017. hnRNPK Recruits PCGF3/5-PRC1 to the Xist RNA B-Repeat to Establish Polycomb-Mediated Chromosomal Silencing. Molecular Cell 68, 955-969.e10. https://doi.org/10.1016/j.molcel.2017.11.013
Reiter, F., Wienerroither, S., Stark, A., 2017. Combinatorial function of transcription factors and cofactors. Current Opinion in Genetics & Development, Genome architecture and expression 43, 73–81. https://doi.org/10.1016/j.gde.2016.12.007
Sönmezer, C., Kleinendorst, R., Imanci, D., Barzaghi, G., Villacorta, L., Schübeler, D., Benes, V., Molina, N., Krebs, A.R., 2021. Molecular Co-occupancy Identifies Transcription Factor Binding Cooperativity In Vivo. Mol Cell 81, 255-267.e6. https://doi.org/10.1016/j.molcel.2020.11.015
Spitz, F., Furlong, E.E.M., 2012. Transcription factors: from enhancer binding to developmental control. Nat Rev Genet 13, 613–626. https://doi.org/10.1038/nrg3207
Yang, M.G., Richter, H.J., Wang, S., McNally, C.P., Harris, N., Dhillon, S., Maresca, M., Wit, E. de, Willenbring, H., Maher, J., Goodarzi, H., Ramani, V., 2025. Pervasive and programmed nucleosome distortion patterns on single mammalian chromatin fibers. https://doi.org/10.1101/2025.01.17.633622

Zhu, F., Farnung, L., Kaasinen, E., Sahu, B., Yin, Y., Wei, B., Dodonova, S.O., Nitta, K.R., Morgunova, E., Taipale, M., Cramer, P., Taipale, J., 2018. The interaction landscape between transcription factors and the nucleosome. Nature 562, 76–81. https://doi.org/10.1038/s41586-018-0549-5
